# Comparison of sparse biclustering algorithms for gene expression datasets

**DOI:** 10.1093/bib/bbab140

**Published:** 2021-05-06

**Authors:** Kath Nicholls, Chris Wallace

**Affiliations:** Cambridge Institute for Therapeutic Immunology and Infectious Disease, University of Cambridge, Cambridge, CB2 0AW, UK; Cambridge Institute for Therapeutic Immunology and Infectious Disease, University of Cambridge, Cambridge, CB2 0AW, UK; MRC Biostatistics Unit, Cambridge Biomedical Campus, Forvie Site, Robinson Way, Cambridge, CB2 0SR, UK

**Keywords:** biclustering, clustering, gene expression, multi-tissue

## Abstract

**Motivation:**

Gene clustering and sample clustering are commonly used to find patterns in gene expression datasets. However, genes may cluster differently in heterogeneous samples (e.g. different tissues or disease states), whilst traditional methods assume that clusters are consistent across samples. Biclustering algorithms aim to solve this issue by performing sample clustering and gene clustering simultaneously. Existing reviews of biclustering algorithms have yet to include a number of more recent algorithms and have based comparisons on simplistic simulated datasets without specific evaluation of biclusters in real datasets, using less robust metrics.

**Results:**

We compared four classes of sparse biclustering algorithms on a range of simulated and real datasets. All algorithms generally struggled on simulated datasets with a large number of genes or implanted biclusters. We found that Bayesian algorithms with strict sparsity constraints had high accuracy on the simulated datasets and did not require any post-processing, but were considerably slower than other algorithm classes. We found that non-negative matrix factorisation algorithms performed poorly, but could be re-purposed for biclustering through a sparsity-inducing post-processing procedure we introduce; one such algorithm was one of the most highly ranked on real datasets. In a multi-tissue knockout mouse RNA-seq dataset, the algorithms rarely returned clusters containing samples from multiple different tissues, whilst such clusters were identified in a human dataset of more closely related cell types (sorted blood cell subsets). This highlights the need for further thought in the design and analysis of multi-tissue studies to avoid differences between tissues dominating the analysis.

**Availability:**

Code to run the analysis is available at https://github.com/nichollskc/biclust_comp, including wrappers for each algorithm, implementations of evaluation metrics, and code to simulate datasets and perform pre- and post-processing. The full tables of results are available at https://doi.org/10.5281/zenodo.4581206.

## 1 Introduction

Clustering can be used in two main ways to analyse gene expression datasets [17]. The first is to cluster the samples, finding groups of samples that have similar expression in all genes. This can be used, for example, to find subgroups of disease [9]. The second is to cluster the genes, finding groups of genes that have similar expression across all samples. Finding such groups of genes has many useful applications such as inferring function using guilt by association and inferring regulatory relationships [30].

Instead of clustering only samples or only genes, biclustering algorithms find groups of samples that have similar expression in some subset of the genes, effectively clustering both genes and samples simultaneously. Such a group is called a ‘bicluster’, and we say that the bicluster consists of a set of samples and a set of genes. Biclustering has three main advantages over normal clustering. Firstly, it can discover meaningful groups that would not be detected using normal clustering; in complex datasets, many interesting groupings of genes will not hold across all samples. For example, we might expect some genes to cluster differently in different cell types, whilst other clusters may be shared across cell types. Traditional methods of clustering cannot account for this because the models expect clusters to exist across all samples. Secondly, biclustering provides a link between sets of genes and sample traits such as disease or sex. For example, if a biclustering algorithm returns a bicluster consisting of all the samples from patients with a given disease and a small set of genes, then we can hypothesise that the set of genes might have biological importance for the disease. Finally, biclustering algorithms reconstruct the gene expression matrix as the sum of effects from each bicluster, allowing the algorithm to learn biclusters corresponding to confounders, such as batch or sex, and adjust for these confounders whilst simultaneously extracting biologically interesting biclusters. In this study, we have focused on identifying algorithms that should be able to identify sparse biclusters in a complex bulk RNA-seq dataset, such as one including samples from multiple cell types.

There exist previous reviews of biclustering algorithms [4, 6, 26, 29], but we hope to improve on them in the following ways. First, we include new classes of algorithm yet to be considered in independent comparison studies. In particular, we include non-negative matrix factorisation (NMF) algorithms, which we believe can be re-purposed for biclustering, tensor factorisation algorithms, which aim to improve performance by sharing information across tissues, and two Bayesian algorithms that allow for a mixture of sparse and dense biclusters. Second, we use more robust metrics. Horta and Campello investigated metrics used to evaluate similarity between biclusterings, and found problems with many of the metrics used by previous comparison papers [14]. In this study, we use one of the two metrics recommended by Horta and Campello, which was shown to satisfy all but one of their criteria. Third, we narrow the gap between real and simulated datasets. Previous reviews have often used unrealistically simplistic simulated datasets, such as using only $K=1,2,3,4,5$ biclusters, leading to discrepancies between the conclusions they draw on simulated and on real datasets [26]. In this study, we simulate datasets from a wider range of complexities, including datasets closer in complexity to real datasets than those included in previous reviews. A final key flaw of existing comparison studies is the lack of evaluation of biclustering ability on real datasets. In the absence of known structure in the real gene expression datasets used for evaluation, previous reviews have evaluated sample clustering ability and gene clustering ability separately. We carefully chose a knockout mouse RNA-seq dataset that allows linked analysis of sample clustering and gene clustering, thus allowing direct evaluation of biclustering on real datasets.

## 2 Methods

Here, we discuss the algorithms compared, the datasets they are tested on and the evaluation metrics used to score their performance. Similar to previous reviews, we use a mixture of simulated and real datasets. Simulated data are important, as they allow more precise evaluation of performance, since the true structure of the data is known. However, it is difficult to exactly mimic the noise and structure of real gene expression datasets, so it is also important to see whether the algorithms can handle the noise structure of real datasets.

### 2.1 Algorithms compared

We chose most promising algorithms from four classes of algorithm, focusing on sparse algorithms (Table 1).

**Table 1:** Summary of algorithms included in comparison. Algorithms are listed in groups: popular algorithms which have been included in previous reviews (Popular), NMF algorithms, tensor factorisation algorithms (Tensor) and Bayesian algorithms allowing for a mixture of sparse and dense biclusters, with strength of sparsity constraints adapting to the bicluster (Adaptive). With the exception of Plaid (Section S1.1), algorithms either factorise the gene expression matrix $Y$ as $Y = XB^T + \varepsilon $ (matrix factorisation), where $X$ is the samples loading matrix, and $B$ is the gene loading matrix, or write it as a tensor product $Y = \sum _k a_k \otimes b_k \otimes z_k + \varepsilon $ (tensor product), where $a_k$ gives the loadings for bicluster $k$ for the individuals, $b_k$ gives the loadings for genes and $z_k$ gives the loadings for the tissues. Some algorithms use one language to implement the algorithm and provide a ‘wrapper’ in another language. Where this occurs, the language given in the ‘Version’ column is the language used to interact with the algorithm (the wrapper), rather than the language that the implementation uses

Class	Name	Model	Sparsity	Version	References
**Popular**	FABIA	Matrix factorisation	Laplacian prior	pyfabia::2016.8 *(Python)*	[12]
	Plaid	Plaid		biclust::2.0.2 *(R)*	[21, 31]
**NMF**	nsNMF	Matrix factorisation	smoothing matrix and non-negativity of $X, B$	nimfa::1.4.0 *(Python)*	[27]
	SNMF	Matrix factorisation	non-negativity of gene loadings matrix $B$	nimfa::1.4.0 *(Python)*	[18]
**Tensor**	MultiCluster	Tensor product	Tissue components $z_k$ non-negative	MultiCluster 15-08-2018 *(MATLAB)*	[32]
	SDA	Tensor product	Spike-and-slab prior on gene components $b_k$	SDA 02-05-2016	[13]
**Adaptive**	BicMix	Matrix factorisation	Three Parameter Beta prior	BicMix 03-08-2019 *(R)*	[8]
	SSLB	Matrix factorisation	Mixture of Laplacian prior	SSLB 24-04-2020 *(R)*	[23]

We define a matrix $Y \in \mathbb{R}^{n \times p}$ where entry $Y_{ij}$ gives the expression of gene $j$ in sample $i$. This can either be the raw read count from an RNA-seq experiment or a normalised count, which has been adjusted for sample-specific effects such as library size, or gene-specific effects such as mean expression level. The typical approach to biclustering is to factorise this matrix as a product of two sparse matrices $X \in \mathbb R ^ {n \times K}$, which we call the sample loadings matrix, and $B \in \mathbb R ^ {p \times K}$, which we call the gene loadings matrix, with error matrix $\varepsilon $:

(1) \begin{align*} Y = XB^T + \varepsilon \end{align*}

The individual algorithms are described in detail in Section S1 along with an explanation of the two main mechanisms used to induce sparsity: NMF and sparsity-inducing priors. Here, we discuss why each algorithm was chosen for inclusion in this study and group the algorithms as ‘Popular’, ‘Adaptive’, ‘NMF’ and ‘Tensor’.

#### 2.1.1 Popular algorithms

We include two algorithms that have been included in previous reviews, which we use as a baseline to allow relative performance to be related to other comparison studies. Factor Analysis for Bicluster Acquisition (FABIA) [12] is a Bayesian algorithm using sparsity-inducing priors, included in a number of previous comparisons [6, 10, 26, 30]. Although our study focuses on sparse biclustering algorithms, we chose to also include Plaid [21, 31] even though it does not enforce sparsity, as it has often appeared as one of the better performing algorithms in other studies [6, 26] and its inclusion thus provides a helpful link to these studies.

#### 2.1.2 Adaptive Bayesian algorithms

Like FABIA, BicMix [8] and Spike-and-Slab Lasso Biclustering (SSLB) [23] use sparsity-inducing priors. The key difference with BicMix and SSLB is that they allow for both sparse and dense biclusters, and adapt the sparsity constraints to each bicluster. Neither has been included in previous comparisons but they have been compared against each other and against FABIA in the paper introducing SSLB, where both achieved much greater sparsity and accuracy than FABIA.

#### 2.1.3 Non-negative matrix factorisation

NMF algorithms in general are not designed for biclustering, but since biclustering can be described as sparse matrix factorisation, NMF algorithms can recover biclusters if they use sufficiently strong sparsity constraints. We chose to include two examples of such algorithms: Sparse non-negative matrix factorisation (SNMF) [18] and non-smooth non-negative matrix factorisation (nsNMF) [27]. The main advantage we expect these algorithms will have is speed, as they are computationally much simpler than many of the others included in this study.

#### 2.1.4 Tensor algorithms

When applying an algorithm to data from multiple cell types, a natural extension to the two-dimensional algorithms presented so far is a three-dimensional algorithm which exploits similarity between corresponding samples in different cell types. This approach is also known as triclustering [11]. We chose to include two algorithms that attempt this: Sparse Decomposition of Arrays (SDA) [13] and MultiCluster [32]. These algorithms recover triclusters, which contain a subset of the individuals, a subset of the tissues and a subset of the genes. Every tricluster can be flattened into a bicluster and, although general biclusters cannot always be converted into a tricluster, all the biclusters in our simulated study are generated as flattened triclusters so they can all be detected by these algorithms. Additionally, we have carefully chosen real datasets that have tricluster structure, to allow triclustering algorithms to be evaluated against biclustering algorithms without disadvantage.

### 2.2 Algorithm parameters

The algorithms evaluated here, outlined in Table 1, have many parameters that can be tuned. Before running the full analysis, we conducted a parameter sweep (Section S2) to see if there were any parameter values that consistently improved the score relative to that when the default values were used. For most algorithm parameters, there was either no clear optimal value, or the default value was optimal. Thus, for most algorithms, we used the default parameters throughout this study. One key exception was BicMix, which has a parameter determining whether or not each gene gets transformed to a Gaussian distribution before the algorithm runs. Changing this parameter had a dramatic but inconsistent effect, so we decided to use two versions of BicMix: BicMix, using default behaviour of not transforming genes, and BicMix-Q, which does apply the Gaussian transformation before analysis. Full discussion of our investigation of parameter sensitivity is given in Section S2.

### 2.3 Simulated datasets

**Figure 1:**
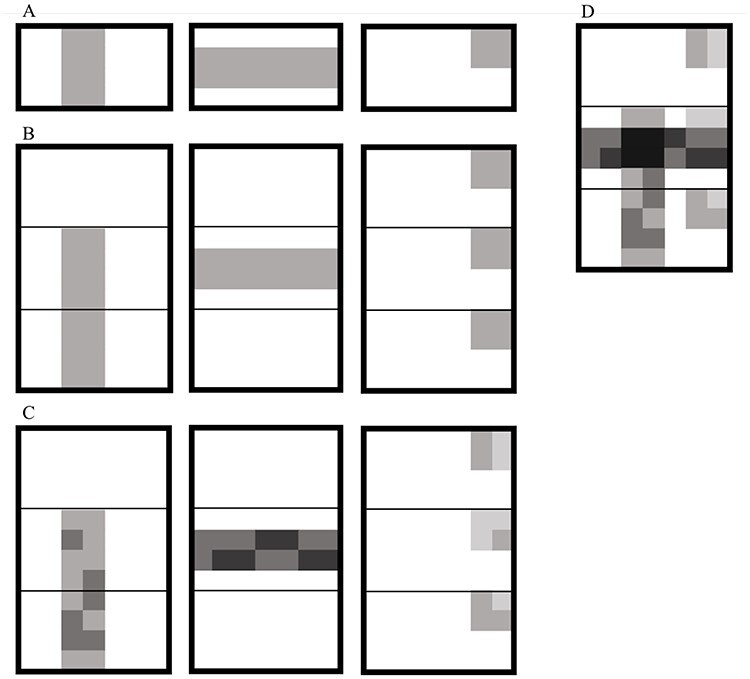
Illustration of process for simulating gene expression datasets with implanted biclusters. In this diagram (A) shows steps 1.a-1.b where membership for genes (columns) and individuals (rows) are sampled for three biclusters, (B) shows step 1.c for the three biclusters, where we extend the biclusters from size $(m_k, g_k)$ to size $(m_kt_k, g_k)$ by sampling membership for tissues, (C) shows steps 1.d and 1.e where values for the bicluster members are sampled, with bicluster-specific means $\mu _k$, (D) shows step 2 where the effects from the three biclusters are added together.

We simulated individual gene expression data for each gene as a sum across biclusters of negative binomial counts. Our base model for generating a gene expression dataset with $p$ genes, $m$ individuals and $t$ tissues and with $K$ potentially overlapping biclusters is illustrated in Figure 1 and described below:

1. For each bicluster $k=1, \dots , K$:
  Select genes to include in bicluster $k$: first draw number of genes $g_k$ uniformly from the set $\{\frac{p}{100}, \frac{p}{10}, \frac{p}{5}, \frac{p}{2}, p\}$ and then pick a random sample of $g_k$ genes.
  Select individuals to include in bicluster $k$: first draw number of individuals $m_k$ uniformly from the set $\{\frac{m}{100}, \frac{m}{10}, \frac{m}{5}, \frac{m}{2}, m\}$ and then pick a random sample of $m_k$ individuals.
  Select tissues to include in bicluster $k$: first draw number of tissues $t_k$ uniformly from the set $\{1, 2, \dots , t\}$ and then pick a random sample of $t_k$ tissues.
  Sample bicluster-specific mean $\mu _k \sim \textrm{Gamma} (\alpha , \beta )$ using $\alpha =2, \beta =\frac{1}{600}$. These parameters give a mean of 1200 and standard deviation of 849. This gives a standard deviation of $63.2$ when the bicluster-specific mean is 1200. We found that this choice of mean distribution gave a good range of scenarios, ranging from biclusters which were easily distinguishable by eye to harder scenarios where biclusters had more similar means (Figure S11).
  Sample values in bicluster using negative binomial distribution with mean $\mu _k$, shared parameter $p=0.3$. This gives a standard deviation of 63.2 when the bicluster-specific mean is 1200.
2. Add together values from all biclusters
3. Add background noise using negative binomial distribution

Formally the base model is:

(2) \begin{align*}& \begin{aligned} Y_{ijl} &= \sum_k \delta_{ik} \gamma_{jk} \tau_{lk} E_{ijl}^{(k)} + B_{ijl} \\ E_{ijl}^{(k)} & \sim \textrm{NegBin} (n_k, p) \\ B_{ijl} & \sim \textrm{NegBin} (1, p) \\ n_k &= \frac{\mu_k p}{1 - p} \\ \mu_k & \sim \textrm{Gamma} (\alpha, \beta) \\ \end{aligned} \end{align*}

where $\delta _{ik}$, $\gamma _{jk}$ and $\tau _{lk}$ are binary indicators of membership of individual $i$, gene $j$ and tissue $l$ to bicluster $k$, respectively, $E_{ijl}^{(k)}$ is the increase in expression of gene $i$ in tissue $l$ in individual $i$ due to bicluster $k$ and $B_{ijl}$ is background noise. We chose to force the genes chosen in a bicluster to belong to a contiguous block rather than allowing genes from a bicluster to be scattered freely throughout the matrix and did the same for the tissues and individuals chosen in a bicluster. This arrangement has little impact on the generality of the data but makes it easier to visualise the datasets.

We vary the size of the dataset, the number of biclusters and the size of biclusters. Although we do not explicitly vary the overlap between biclusters, this varies between datasets, driven mainly by the number of biclusters (Figure S12). For datasets with ‘sparse’ biclusters, in step 1.a the number of genes $g_k$ is chosen uniformly from the set $\{\frac{p}{20}, \frac{2p}{20}, \frac{3p}{20}\}$ instead of the default list and similarly in step 1.b the number of individuals $m_k$ is chosen uniformly from $\{\frac{m}{20}, \frac{2m}{20}, \frac{3m}{20}\}$. For datasets with ‘dense’ biclusters, in step 1.a the number of genes $g_k$ is chosen uniformly from the set $\{\frac{3p}{10}, \frac{5p}{10}, \frac{9p}{10}\}$ instead of the default list and similarly in step 1.b the number of individuals $m_k$ is chosen uniformly from $\{\frac{3m}{10}, \frac{5m}{10}, \frac{9m}{10}\}$. Bicluster size is called ‘square’ if, for each bicluster, the proportion of genes included and proportion of samples included is the same.

We also introduce diversity by using different noise distributions. For Gaussian noise we use $E_{ijl}^{(k)} \sim \mathcal{N} (\mu _k, \sigma ^2)$ and for noiseless datasets we use $E_{ijl}^{(k)}=\mu _k, B_{ijl}=0$.

Previous reviews have also varied simulation parameters but have often used very small ranges such as $K=1,2,3,4,5$ [26]. Real gene expression datasets are likely to be more complex than this, so we have used larger values of $K$: Most of our simulated datasets have $K=20$ but we consider values from $K=5$ to $K=400$.

The ‘Tensor’ algorithms require an explicit breakdown of the samples into tissues. By listing the samples from each tissue in turn, with individuals in the same order within each tissue, we are able to use the ‘Tensor’ algorithms on the same datasets as the remaining algorithms, allowing direct comparison between the classes of algorithm.

#### 2.3.1 Shift-scale datasets

In addition to the datasets described thus far, whose properties are summarised in Table 2, we include datasets which sample bicluster values using shifting and scaling patterns. The shift-scale model of bicluster values is described in [1] and has been considered in previous comparison studies [4, 6, 26] and can be written as:

(3) \begin{align*}& E_{ijl}^{(k)} = \alpha_j \times \pi_{il} + \beta_j \end{align*}

where $\alpha _j$ is the gene-specific scale parameter, $\beta _j$ is the gene-specific shift parameter and $\pi _{il}$ is the base value for the sample from individual $i$ and tissue $l$. Note that previous studies have sampled $\beta _j$ and $\alpha _j$ and $\pi _{il}$ either uniformly from the range $[0,1]$ [4] or from $ \mathcal{N}(0,1)$ [6, 26]. We sample values as follows:

(4) \begin{align*}& \begin{aligned} \pi_{il} &\sim \textrm{Exponential}(1)\\ \alpha_{j} &\sim \textrm{Exponential}(2) + 1/2\\ \beta{j} &\sim \textrm{Exponential}(1)\\ \end{aligned} \end{align*}

so that base values $\pi _{il}$ and shift parameters $\beta _{j}$ have mean 1 and mode 0 but scale parameters $\alpha _{j}$ have a mean of 1 and a mode of $1/2$. This different distribution for $\alpha _{j}$ avoids having scale parameters too close to 0, which would flatten the signal. Note that using the Exponential distribution avoids negative values, allowing the datasets to be used with the ‘NMF’ algorithms. As in previous studies, we also consider a ‘scale’ model that has $\beta _{j}=0$ and a ‘shift’ model that has $\alpha _{j}=1$. We also consider a model we call ‘constant-samples’ that has $\alpha _{j}=1, \beta _{j}=0$. In all these datasets ‘constant-samples’, ‘scale’, ‘shift’ and ‘shift-scale’, we use no noise i.e. $B_{ijl}=0$.

**Table 2:** Summary of simulated datasets. The attributes of the datasets are displayed in bold if they differ from the base dataset. N is the number of individuals, T the number of tissues and G the number of genes in the dataset

Name	N	T	G	Bicluster sizes	K	Noise
Base	10	10	1000	Mixed	20	$\textrm{NB}(n_{k}, 0.3)$
N50-T2	**50**	**2**	1000	Mixed	20	$\textrm{NB}(n_{k}, 0.3)$
N10-T20	10	**20**	1000	Mixed	20	$\textrm{NB}(n_{k}, 0.3)$
N100-T10	**100**	10	1000	Mixed	20	$\textrm{NB}(n_{k}, 0.3)$
N500-T10	**500**	10	1000	Mixed	20	$\textrm{NB}(n_{k}, 0.3)$
G100	10	10	**100**	Mixed	20	$\textrm{NB}(n_{k}, 0.3)$
G5000	10	10	**5000**	Mixed	20	$\textrm{NB}(n_{k}, 0.3)$
Large-K20	**300**	**20**	**10 000**	Mixed	20	$\textrm{NB}(n_{k}, 0.3)$
Negbin-medium	10	10	1000	Mixed	20	$\textrm{NB}(n_{k}, \textbf{0.1})$
Negbin-high	10	10	1000	Mixed	20	$\textrm{NB}(n_{k}, \textbf{0.01})$
Gaussian	10	10	1000	Mixed	20	$\boldsymbol{\mathcal{N}(\mu _{k}, 20^2)}$
Gaussian-medium	10	10	1000	Mixed	20	$\boldsymbol{\mathcal{N}(\mu _{k}, 100^2)}$
Gaussian-high	10	10	1000	Mixed	20	$\boldsymbol{\mathcal{N}(\mu _{k}, 300^2)}$
Noiseless	10	10	1000	Mixed	20	**No noise**
Sparse	10	10	1000	**Sparse**	20	$\textrm{NB}(n_{k}, 0.3)$
Dense	10	10	1000	**Dense**	20	$\textrm{NB}(n_{k}, 0.3)$
Sparse-square	10	10	1000	**Sparse, square**	20	$\textrm{NB}(n_{k}, 0.3)$
Dense-square	10	10	1000	**Dense, square**	20	$\textrm{NB}(n_{k}, 0.3)$
K5	10	10	1000	Mixed	**5**	$\textrm{NB}(n_{k}, 0.3)$
K10	10	10	1000	Mixed	**10**	$\textrm{NB}(n_{k}, 0.3)$
K50	10	10	1000	Mixed	**50**	$\textrm{NB}(n_{k}, 0.3)$
K70	10	10	1000	Mixed	**70**	$\textrm{NB}(n_{k}, 0.3)$
Large-K100	**300**	**20**	**10 000**	Mixed	**100**	$\textrm{NB}(n_{k}, 0.3)$
Large-K400	**300**	**20**	**10 000**	Mixed	**400**	$\textrm{NB}(n_{k}, 0.3)$

### 2.4 Real datasets

A key limitation of the existing reviews of biclustering algorithms is their inability to assess ‘simultaneous’ clustering of samples and genes on real datasets, due to the absence of known biclusters in the data. In order to have predictable bicluster structure in a real dataset, we chose to use a knockout mouse dataset [19, 33]. We proposed that a successful algorithm would recover, for each of the 106 knockout genes, a bicluster containing the roughly 20 samples where the gene was knocked out and enriched for genes that share a pathway with the knocked-out gene. Thus this dataset allows us to have some sense of its true bicluster structure.

#### 2.4.1 International Mouse Phenotyping Consortium dataset

We use the RNA-seq dataset available on ArrayExpress under accession number E-MTAB-5131, part of the International Mouse Phenotyping Consortium (IMPC) [19, 33]. It consists of 106 knockout genotypes, from each of which are available roughly three replicates in each of up to seven tissues. There are also samples from wild-type mice.

To make the study feasible for multiple algorithms in terms of computational time, we chose to restrict to a subset of genes. We restricted to the 4444 genes which share a Reactome pathway with at least one of the 106 knockout genes, found by searching the Reactome pathways [7, 16] using Mouse Mine [24]. We apply three different normalisation methods to the data: (1) library size adjustment using DESeq’s median of ratios normalisation method, (2) the log transform $x \to \log{(x + 1)}$, which is commonly used in analysis of gene expression data and (3) Gaussian quantile normalisation so that each gene has approximate $N(0, 1)$ distribution.

#### 2.4.2 Tensor structure

The ‘Tensor’ algorithms require the dataset to have three dimensions i.e. $m$ individuals, $t$ tissues, $p$ genes rather than just $n=m \times t$ samples and $p$ genes. We chose the 3 tissues with the most samples (liver, lung and cardiac ventricle), and the 64 genotypes with at least one sample in each of these tissues. Unfortunately we were unable to find information detailing which samples came from which specific mouse replicate so could not simply include a row for each individual, a column for each gene and a layer for each tissue. Instead we pooled the samples from each genotype for each tissue individually by taking the mean of the replicates. Thus, we had $m=64,t=3$ with a total of $n=192$ samples.

This type of dataset, which we call the ‘Tensor’ dataset, can be used by all the algorithms, whereas the ‘non-tensor’ dataset, which simply uses all $n=1143$ samples, can not be used by the ‘Tensor’ algorithms.

#### 2.4.3 Benaroya sorted blood cell dataset

We use the RNA-seq dataset available on ArrayExpress under accession number E-GEOD-60424, collected by the Benaroya Research Institute [22]. We use a subset consisting of 20 samples from each of six immune cell types, including subjects with amyotrophic lateral sclerosis, multiple sclerosis, type 1 diabetes and sepsis, along with healthy controls (Table S4). For the subjects with multiple sclerosis, samples were taken before and after treatment with IFN-beta. As with the IMPC dataset, we tried both log transformation and DESeq’s median of ratios size factor normalisation method. We restrict to the 17 069 protein-coding genes that are expressed in at least one cell type (i.e. with median expression greater than $0$ in at least one cell type). This dataset has tensor structure, so can be used by all the algorithms.

### 2.5 Evaluation metrics

We use a range of metrics to evaluate performance of the biclustering algorithms (Table 3). In particular, we made use of an extensive study of biclustering accuracy metrics [14] to choose the *clustering error* (CE) metric [14, 28] to evaluate biclustering accuracy. Horta and Campello defined eight desirable properties, such as penalising the omission of biclusters, penalising the inclusion of extra copies of biclusters and penalising the merging of biclusters. The CE metric was shown to satisfy all of these eight properties except ‘homogeneity’. This is a great improvement on the consensus score and recovery and relevance scores commonly used to evaluate biclustering similarity. Notably, these commonly used metrics would not penalise a algorithm for returning extra copies of a bicluster.

**Table 3:** Metrics used to evaluate the biclustering algorithms, which are described more fully in Methods Section ‘Evaluation metrics’. For each metric we note what information about the dataset it requires, the best and worst scores theoretically possible and briefly describe what it measures

Name	Requires	Best	Worst	Measures
CE	Entire bicluster structure	1	0	Biclustering accuracy
NRE	–	0	1	Reconstruction error
MBR	–	0	1	Similarity between biclusters within a run
Similarity between runs	Multiple runs	1	0	Similarity between runs
Sample clustering	Sample clusters	1	0	Sample clustering
Pathway enrichment	Pathway database	1	0	Gene clustering
Relevant pathway enrichment	Known link between pathways and sample clusters	1	0	Biclustering

Most metrics used by previous reviews, including the CE metric that we intend to use for evaluation of performance on simulated datasets, cannot be used on real datasets, as they require knowledge of the entire biclustering structure of the dataset. We introduce two metrics that can be used even when nothing is known about the structure of the dataset: Normalised Reconstruction Error (NRE) and Mean Biclustering Redundancy (MBR).

#### 2.5.1 Clustering error

The CE [14, 28] is defined as:

(5) \begin{align*}& \textrm{CE}(A, \hat{A}):=\frac{d_{\mathrm{max}}}{|U|} \end{align*}

where $d_{\mathrm{max}}$ measures how much the biclusterings intersect and $|U|$ measures the total space collectively covered by the biclusterings, taking overlaps into account (Section S3). The Hungarian algorithm [25] is used to find a pairing of biclusters from the two sets such that the sum of the intersections between pairs is maximised. Despite its name, CE is a measure of similarity between biclusters rather than dissimilarity.

#### 2.5.2 Normalised reconstruction error

Most metrics used by previous reviews, including the ‘CE’ metric that we intend to use, can only be used on simulated datasets. We introduce two metrics that can be used even when nothing is known about the structure of the dataset. The first uses the error matrix $\varepsilon = Y - \hat{X}\hat{B}^T = Y - \hat{Y}$ to see how similar the recovered factorisation is to the original matrix.

Given original matrix $Y$, and factorisation $\hat{Y} = \hat{X}\hat{B}^T$ returned by the algorithm, we define the NRE as:

(6) \begin{align*}& \textrm{NRE}(Y, \hat{Y}):= \frac{\| Y-\hat{Y} \|_{F}}{\| Y \|_{F}+ \| \hat{Y} \|_{F}} \end{align*}

where $\| A \|_{F}$ denotes the Frobenius norm $\sqrt{\sum _{ij} A_{ij}^2}$.

A score of 0 indicates perfect reconstruction. The maximum score is 1, indicating a large error relative to the true matrix $Y$ and the recovered matrix $\hat{Y}$. One big advantage of this metric is that it can be used on real datasets too, since all that is needed is the original matrix $Y$. It should be noted that this measure may reward algorithms or parameter settings which are overly complex and are thus able to overfit to the data. To avoid overfitting, we recommend avoiding direct optimisation with respect to this measure, unless it is used in combination with a measure that penalises model complexity.

It should be noted that this metric requires the algorithm to factorise the raw matrix as the product of two matrices, and to return the raw values from this factorisation. Plaid is the only algorithm we consider which does not perform explicit factorisation, however BicMix-Q, MultiCluster and FABIA all apply transformations to the raw matrix before factorisation, so also cannot be evaluated using this metric.

**Figure 2:**
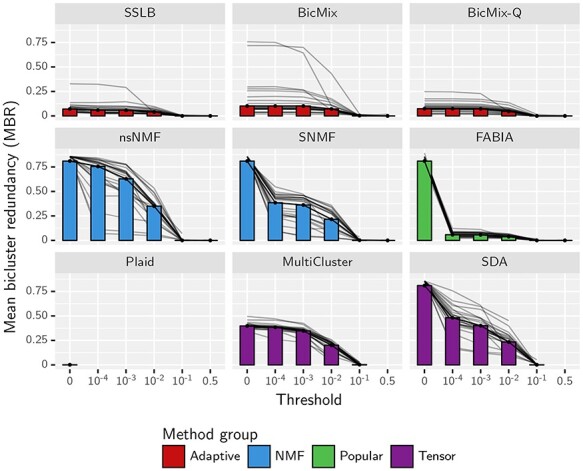
MBR within a run plotted against the threshold for inclusion in bicluster, for simulated datasets. MBR (Methods Section ‘Mean Bicluster Redundancy’) is in the range $[0,1]$, and a lower value is preferred as it suggests that the algorithm is not returning biclusters that are very similar to each other. The thresholding process is described in Section S6. The median of this measure across all simulated datasets and all values of $K_{\textit{init}}$ is shown by the bars. The grey lines show the median for each dataset type. Note that without thresholding (threshold 0) the biclusters within each run by FABIA, SDA, SNMF and nsNMF are almost all identical.

#### 2.5.3 Mean Bicluster Redundancy

Another metric we introduce that can be used without knowledge of the structure of the dataset is the MBR, which measures how similar the biclusters returned in a single run are to each other. When running biclustering algorithms on large datasets, it can be difficult to interpret the results if the algorithms return many copies of the same bicluster. The perfect score of 0 indicates that the biclusters do not overlap at all, and the worst score of 1 indicates that all biclusters are identical.

The Jaccard index [15] measures how closely two sets match, comparing their intersection to their union. We construct a matrix $J$ using the Jaccard index between each pair of biclusters:

(7) \begin{align*}& J_{k l}:= \frac{\left| A_{k} \cap A_{l} \right|}{\left| A_{k} \cup A_{l} \right|} \end{align*}

and then take the mean of the off-diagonal entries:

(8) \begin{align*}& \textrm{MBR}(A):= \frac{2}{K(K-1)} \sum_{k=1}^{K-1} \sum_{l=k+1}^K J_{k l} \end{align*}

#### 2.5.4 Sample clustering in real datasets

The samples in the IMPC dataset can be clustered in two main ways: by tissue or by genotype. We refer to these as ‘sample traits’. We chose to measure how well a bicluster matches a given sample trait using the $F_1$ score [5], which balances reward for containing ‘only’ elements with the sample trait (precision) and reward for containing ‘all’ the elements with that sample trait (recall). It is defined as:

(9) \begin{align*}& F_{1} \textrm{ score} = 2 \times \frac{\textrm{ Precision} \times \textrm{ Recall}} {\textrm{ Precision }+\textrm{ Recall }} \end{align*}

where precision and recall are defined in terms of the set $S$ of samples with the trait and set $F$ of samples contained in the bicluster:

(10) \begin{align*}& \begin{aligned} \textrm{ Precision } &= \frac{|S \cap F |}{|F|} \\ \textrm{ Recall } &= \frac{|S \cap F |}{|S|} \end{aligned} \end{align*}

For each trait, we find the best $F_1$ score across all biclusters, and then take the mean of these maximum $F_1$ scores across sample traits. We call the mean of this maximum across all traits the sample clustering ability, the mean across only tissue traits we call the tissue clustering ability and the mean across only genotype traits we call the genotype clustering ability.

**Figure 3:**
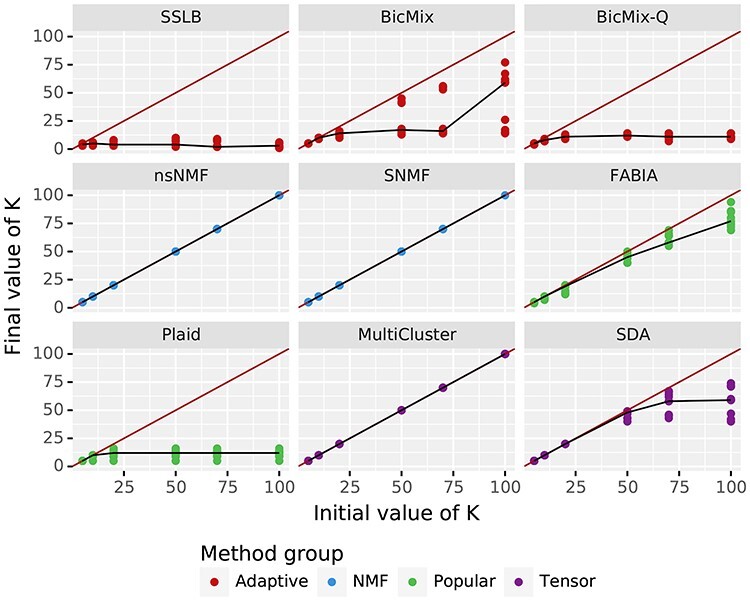
Robustness to choice of $K_{\textit{init}}$, shown by final value of K (after post-processing) plotted against initial value of K. There is a point for each of the three seeds of the ‘base’ dataset, which has $K=20$. The red line shows the limit, where the final value of K is the same as the initial value of K. The black line joins the median $\hat{K}$ across all seeds of the ‘base’ dataset. The ideal behaviour is for the line to be flat once $K_{\textit{init}}$ exceeds the true K (for this dataset $K=20$), showing that the algorithm converges to the same value of K regardless of the initial value given, as long as $K_{\textit{init}}$ is sufficiently large. MultiCluster and ‘NMF’ algorithms always returned the same number of biclusters as they started with. Most algorithms show a similar pattern in terms of number of biclusters returned, regardless of the true number of biclusters (Figure S23).

#### 2.5.5 Gene clustering in real datasets

The typical approach to evaluate gene clustering in real datasets is to look at what proportion of biclusters are enriched for at least one pathway in some pathway database such as GO, Reactome or KEGG [4, 6, 12, 26, 29, 34]. We look at what proportion of the biclusters returned by each algorithm are enriched for at least one Reactome pathway [7, 16] (restricted to Reactome pathways containing at least one of the 106 genes knocked out in this experiment), measured by Fisher’s one-tailed hypergeometric test, with *P* values adjusted for multiple testing by the Benjamini–Yekutieli adjustment [3].

#### 2.5.6 Biclustering in real datasets

We identify, for each knocked-out gene, the bicluster that a algorithm has recovered, which most closely matches the samples from that knockout genotype, using the $F_1$ score. Then we look at whether this bicluster is also enriched for genes that share a pathway with the knocked-out gene, using Fisher’s hypergeometric test. This evaluation of simultaneous sample clustering and gene clustering is one aspect of our study that is unique among comparisons of biclustering algorithms, and we think it is a very important inclusion.

**Table 4:** Summary of results, with the best score for each measure underlined and in bold and scores close to the best score underlined and in italics. Unless otherwise stated, the measures are given for all runs using $K_{\textit{init}}$ as described in Methods Section ‘Choice of $K_{\textit{init}}$’ and after the standard thresholding has been applied. Runs that failed (Table S5 and Table S6) are discarded in the analysis. ‘Tensor’ algorithms could not be run on non-tensor datasets so these entries are marked ‘N/A’. (A) Clustering Error (CE) across all simulated datasets. (B) Reconstruction error (NRE) across all simulated datasets. Algorithms which did not return the raw values required to calculate this measure are marked with ‘^*^’. (C-D) Average similarity between recovered biclusters (MBR) before (raw) and after (thresholded) the standard thresholding has been applied. (E) Correlation between $K_{\textit{init}}$ and CE, best score is 0, indicating that score was unaffected by $K_{\textit{init}}$. (F) Correlation between $K_{\textit{init}}$ and $K_{\textrm{recovered}}$, best score is 0, indicating that the algorithm converged to the same number of biclusters regardless of $K_{\textit{init}}$. (G) Correlation between $K_{\textrm{recovered}}$ and $K_{\textrm{true}}$ when $K_{\textit{init}}=100$, an overestimate of $K_{\textrm{true}}$. The best score is 1. Entries marked ‘^*^’ correspond to algorithms that returned $K_{\textrm{recovered}}=K_{\textit{init}}$ for all runs, for which correlation could not be calculated. (H) Similarity between all ten runs on each IMPC dataset. (I-L) Performance on IMPC datasets with tensor structure with $K_{\textit{init}}=50$, using measures described in Methods Section ‘Evaluation metrics’. (M-P) Same as H-K but for IMPC datasets with non-tensor structure. Note that the ‘Tensor’ algorithms were not run on the non-tensor datasets (Q-R) Time to run in seconds on the datasets of type *K5*, *K10*, *base*, *K50* and *K70*, with $K_{\textit{init}}=20$ and $K_{\textit{init}}=100$, respectively. (S) Time to run in seconds for the largest simulated dataset *large-K400*, when the algorithms were run with a small number of biclusters ($K_{\textit{init}}=20$ for all algorithms except SSLB, BicMix, BicMix-Q which used $K_{\textit{init}}=25$). Plaid failed to complete any runs on this largest dataset and is marked as ‘^*^’. (T-U) for the largest IMPC datasets, where the algorithms used a large number of biclusters ($K_{\textit{init}}=200$). Note that the ‘Tensor’ algorithms were not run on the non-tensor datasets

		*Adaptive*			*NMF*		*Popular*		*Tensor*	
		SSLB	BicMix	BicMix-Q	nsNMF	SNMF	FABIA	Plaid	MultiCluster	SDA
	**Simulated datasets**									
(A)	Biclustering accuracy (CE)	**0.366**	*0.3*	0.261	0.165	0.239	0.21	0.145	0.173	0.173
(B)	Reconstruction error (NRE)	**0.0683**	**0.0739**	^*^	0.137	0.18	^*^	^*^	^*^	*0.0939*
	**Ease of use**									
(C)	Redundancy (MBR - thresholded)	*0.0618*	0.134	*0.0692*	0.34	0.239	**0.0441**	**0.00321**	0.192	0.259
(D)	Redundancy (MBR - raw)	*0.129*	0.249	*0.124*	0.914	0.912	0.901	0.00321	0.439	0.913
(E)	Robustness to $K_{\textit{init}}$ (CE)	**−0.0651**	*−0.269*	**−0.008**	−0.774	−0.748	*−0.16*	**0.0202**	−0.66	−0.392
(F)	Robustness to $K_{\textit{init}}$ (Recovered K)	*0.321*	0.782	*0.445*	1	1	0.962	**0.201**	1	0.955
(G)	Recovery of $K_{\textrm{true}}$	−0.484	0.0289	**0.836**	^*^	^*^	0.432	0.0483	^*^	0.0421
(H)	Similarity between runs	0.283	0.342	0.405	0.521	0.606	0.248	0.602	**1**	0.394
	**Tensor IMPC datasets**									
(I)	Tissue clustering (tensor)	0.822	0.754	0.579	*0.934*	0.752	0.658	0.666	**0.994**	**0.966**
(J)	Genotype clustering (tensor)	*0.167*	0.123	0.129	0.045	0.0406	**0.253**	0.0493	0.0538	0.0507
(K)	Gene clustering (tensor)	0.771	0.68	0.408	**0.997**	0.647	0.834	**0.977**	*0.94*	0.801
(L)	Relevant pathway clustering (tensor)	0.255	0.12	0.0981	**0.53**	0.3	0.242	0.328	0.35	0.24
	**Non-tensor IMPC datasets**									
(M)	Tissue clustering (non-tensor)	**0.951**	0.708	0.521	0.671	*0.871*	0.787	0.641	N/A	N/A
(N)	Genotype clustering (non-tensor)	*0.088*	0.0648	0.0521	0.0355	0.0483	**0.122**	0.0406	N/A	N/A
(O)	Gene clustering (non-tensor)	*0.956*	0.762	0.691	**1**	0.915	*0.969*	**1**	N/A	N/A
(P)	Relevant pathway clustering (non-tensor)	0.36	0.221	0.177	**0.483**	*0.445*	0.325	*0.433*	N/A	N/A
	**Time**									
(Q)	Time ($K_{\textit{init}}=20$)	10.1	52.1	12.6	**1.49**	11.9	5.41	4.75	14.8	45.9
(R)	Time ($K_{\textit{init}}=100$)	101	666.7	*22.7*	**1.93**	513.6	39	*7.3*	*17.3*	612.8
(S)	Time (large-K400 dataset)	6801.9	11250.6	29587.7	*263*	**146.4**	1459.4	^*^	*696.1*	4746.9
(T)	Time (tensor IMPC datasets)	3904.3	354.2	837.9	**6.85**	29107.7	749	*90.7*	*40.7*	3330.3
(U)	Time (non-tensor IMPC datasets)	19579.8	8631.4	10134.4	**25.7**	27194.5	5119.7	*383.1*	N/A	N/A

**Figure 4:**
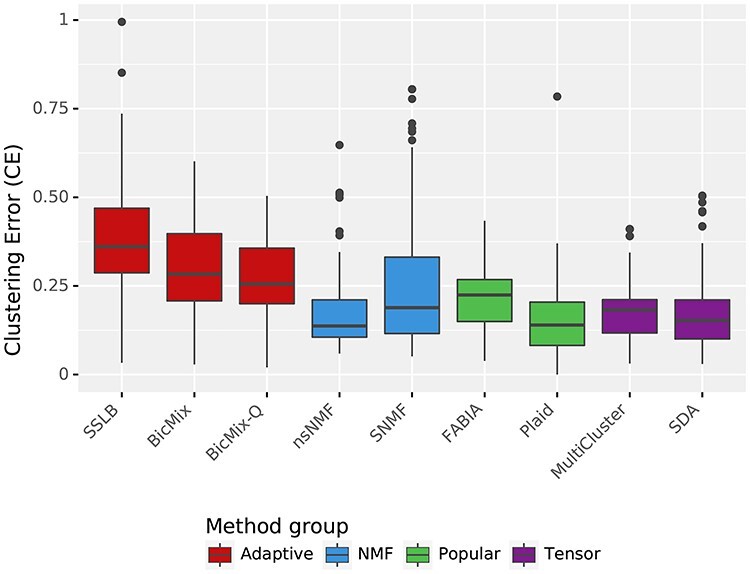
CE across all simulated datasets. The score is in the range $[0,1]$ with larger values preferred. Datasets used are described in Table 2. $K_{\textit{init}}$ is as described in Methods Section ‘Choice of $K_{\textit{init}}$’ and standard thresholding has been applied. Runs that failed (Table S5) are discarded in the analysis.

### 2.6 Post-processing

After looking at the raw output, we decided that we would first need to apply some post-processing steps in order to allow meaningful comparison of the algorithms. The process is described fully in Section S6 and summarised here. The ‘Tensor’ algorithms, ‘NMF’ algorithms and FABIA returned many biclusters containing all genes and all samples (Figure S13 and Figure S14). We found that removing elements in the matrices below a certain threshold, a process we call ‘thresholding’, helped to reveal the biclusters within the noisy raw output. Without thresholding, the biclusters returned by FABIA, SDA and the ‘NMF’ algorithms were highly redundant but this redundancy was reduced by thresholding with a threshold of 0.01 (Figure 2). The optimal threshold is similar for most algorithms, both on simulated datasets (Figure S15) and real datasets (Figure S16), and is largely independent of the metric used to select the threshold.

In particular, we note that we could have chosen a suitable threshold using only measures available for real datasets, such as MBR and NRE. The MBR improves considerably for each increase in threshold, particularly for the ‘NMF’ algorithms and ‘Tensor’ algorithms (Figure 2). Combined with the fact that NRE drastically deteriorates for thresholds above $0.01$ (Figure S17), we can again conclude that a threshold of $0.01$ is ideal, using only measures available on real datasets.

After this careful analysis on real and simulated datasets, we chose to use a threshold of 0.01 for the remainder of the study. We found this value to be robust across datasets and algorithms, although we note there is potential for further fine-tuning.

It is worth highlighting that Plaid and the ‘Adaptive’ algorithms did not require this post-processing step, but in the interests of avoiding bias and unnecessary complications in the analysis we apply the same post-processing steps for every algorithm. The one exception is Plaid, whose implementation returns only the binary membership variables so thresholding cannot be applied. The fact that these algorithms perform well without need for post-processing is a key advantage in terms of ease of use.

### 2.7 Choice of $K_{\textit{init}}$

Overall, algorithms were poor at accurately recovering the right number of biclusters (Figure S21), with only FABIA and BicMix-Q showing any positive correlation between the true K and recovered K (BicMix-Q had correlation of 0.836 between true K and recovered K). Ideally we would simply use a large value of $K_{\textit{init}}$ for all algorithms, as this is what we would do in practice on a real dataset with unknown structure. However, only Plaid, SDA and the ‘Adaptive’ algorithms have shown that they would effectively learn the number of biclusters to include, although it should be noted that all recovered fewer biclusters than the true number of biclusters except SDA which recovered more than the true number of biclusters. The remaining algorithms consistently returned the same number of biclusters as they started with, so did not ‘learn’ K at all (Figure 3). The ‘Adaptive’ algorithms achieve better performance when started with an overestimate of the number of biclusters (Figure S22). Thus, we use $K_{\textit{init}}=K$, the true number of biclusters for all algorithms, except for the ‘Adaptive’ algorithms for which we use a slight overestimate of $K_{\textit{init}}$ ($K_{\textit{init}}=K + 10$, except for when $K=20$, when we use $K_{\textit{init}}=25$.). Note that our way of choosing $K_{\textit{init}}$ is dependent on knowing the true number of biclusters, so it gives the algorithms an advantage they would not have on real datasets. However, it allows us to compare the ‘ideal’ behaviour of each algorithm. For the real datasets we use $K_{\textit{init}}=50, 200$ for all algorithms.

### 2.8 Reproducibility

The datasets used in this study are publicly available at ArrayExpress under accession numbers E-GEOD-60424 and E-MTAB-5131. Additionally, all the code used to run the analysis is available on GitHub (https://github.com/nichollskc/biclust_comp). This includes wrappers for each algorithm, implementations of the evaluation metrics, code used to simulate datasets, and to perform pre- and post-processing. We used random seeds to ensure the analysis would be reproducible. To make the code easier to understand and run, we organised the workflow using the bioinformatics pipeline tool snakemake [20] and kept track of dependencies using the open source package management tool conda [2]. Additionally, we have saved the results files at https://doi.org/10.5281/zenodo.4581206 to allow further investigation of the results without the need to re-run the entire workflow.

## 3 Results

### 3.1 Results on simulated datasets

The results are summarised in Table 4. Figure 4 shows the biclustering accuracy of the algorithms across all the simulated datasets. The ‘Adaptive’ algorithms performed best, with SSLB having the best overall accuracy on simulated datasets (0.336). SNMF has the best accuracy of the non-Bayesian algorithms (0.239).

The accuracy of the algorithms generally decreases as the size of the dataset increases (Figure S24), and as the number of biclusters increases (Figure S25). We had expected algorithms to perform better when there were fewer biclusters, which is the case for SSLB and the ‘NMF’ algorithms. However, FABIA, BicMix and BicMix-Q have poor accuracy on the datasets with small number of biclusters. We measured the amount of overlap between biclusters in the simulated datasets and found that SSLB, Plaid and the ‘NMF’ algorithms performed worse when overlap was higher, with the remaining algorithms showing little change, or improved performance (Figure S12). For the very largest datasets (large-K100 and large-K400) many algorithms took a long time to run and only MultiCluster and nsNMF completed runs within 12 h when using $K_{\textit{init}}$ close to 400 (Table S7 shows failure counts across all runs, and Table S5 shows failure counts restricted to the value of $K_{\textit{init}}$ chosen for analysis).

**Figure 5:**
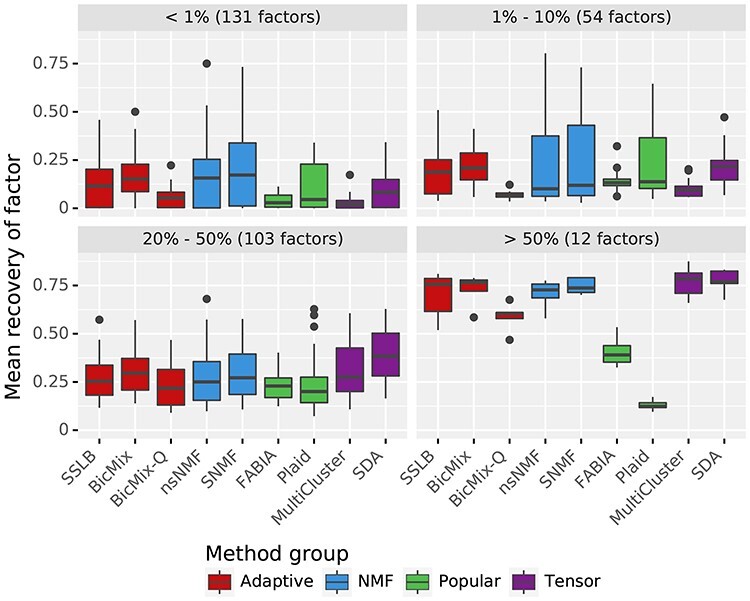
Mean recovery of true biclusters, grouped by size of true biclusters (fraction of total matrix area taken up by true bicluster). We restrict to the datasets ‘base’, ‘sparse’, ‘dense’, ‘sparse-square’ and ‘dense-square’. For each true bicluster in these datasets and for each algorithm, we find the recovered bicluster achieving maximum Jaccard index with the true bicluster. We call this the recovery score for that true bicluster and that algorithm, which is a measure of how well the algorithm has recovered a particular true bicluster. This plot shows the spread of recovery scores for each algorithm, grouped by the proportion of the total area of the dataset taken up by the true bicluster. Recovery scores are generally better for denser biclusters, though Plaid has notably lower recovery scores for the densest biclusters compared to other algorithms.

Changing the sparsity of the biclusters in the simulated datasets had a large effect on accuracy. We had expected that on the datasets with only very sparse biclusters, the ‘Adaptive’ algorithms would have best accuracy as they have the strongest sparsity constraints but in fact the ‘NMF’ algorithms performed best on these datasets (Figure S26). We looked at how the recovery of true biclusters was affected by the sparsity of the bicluster and found that most algorithms achieved better recovery scores for denser biclusters (Figure 5). Since they are smaller, sparse biclusters naturally contain less signal than dense biclusters, so it is expected that they are harder to detect than dense biclusters. Although average recovery for sparse biclusters is poor, some sparse biclusters are recovered perfectly, most notably by Plaid and the ‘NMF’ algorithms (Figure S27). As well as considering how well the algorithms recover the true biclusters, we can look at how relevant the biclusters they recover are. An algorithm that perfectly recovered only one of the 20 true biclusters would have relevant biclusters but poor overall recovery. We found that the algorithms returned many biclusters that did not closely match any true bicluster, including low relevance scores for returned biclusters that are sparse (Figure S28). The ‘NMF’ algorithms and ‘SDA’ returned fewer sparse biclusters with very low relevance, and indeed fewer biclusters in general with low relevance.

**Figure 6:**
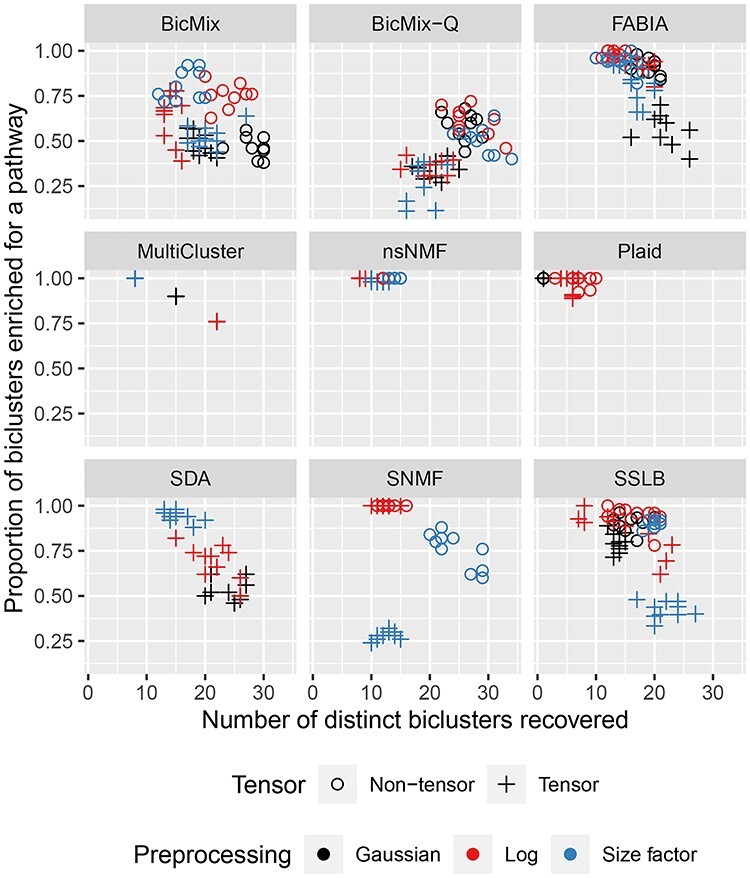
Gene clustering ability, measured by the mean proportion of recovered biclusters that are enriched for at least one pathway against number of distinct biclusters recovered. Pathway enrichment is measured using the one-tailed hypergeometric test adjusted for multiple testing using the Benjamini–Yekutieli correction, we use a significance threshold of $P<0.01$. The median of this measure is shown for each algorithm, with a point for each version of the IMPC dataset. The number of distinct biclusters recovered is measured as the number of pathways that had highest enrichment. Thus an algorithm that recovered only one bicluster and an algorithm with all 20 biclusters all enriched for the same pathway would both show as having only discovered one distinct bicluster. Pathway enrichment is fairly high for all algorithms, especially on the non-tensor datasets. Whilst nsNMF has high pathway enrichment, it recovers fewer distinct biclusters than SSLB or FABIA. Runs that failed (Table S6) are discarded in the analysis.

The algorithms were fairly robust to noise, with little difference in performance between datasets using Negative Binomial noise, Gaussian noise and no noise and only Plaid showing significant decrease in accuracy as noise was increased (Figure S29).

Consistent with the findings in previous studies, the performance of algorithms varies considerably between bicluster models (Figure S30). SSLB has best performance on all shift-scale models except ‘scale’ and stands out from the other algorithms in models involving shift. It should be noted that, as with previous reviews, the ‘scale’ model will flatten some of the signal, marking structure harder for algorithms to discover. Additionally, we note that the scale model can be transformed to a shift model by applying a log transformation.

The algorithms were poor at recovering the correct number of biclusters (Figure S21) and, when started with approximately the right number of biclusters (as described in Section Choice of $K_{\textit{init}}$), Plaid and the ‘Adaptive’ algorithms returned fewer than the true number of biclusters (Figure S31). Although the remaining algorithms recovered the correct number of biclusters in the main study, this is not a significant result, as these algorithms were found to return $K_{\textit{init}}$ biclusters regardless of the true number of biclusters (Figure S21).

### 3.2 Results on IMPC dataset

The algorithms performed well at finding biclusters corresponding to tissues, with many achieving near perfect performance (Table 4, Figure S32). The algorithms were less effective at finding biclusters corresponding to genotypes, with FABIA and SSLB the top two algorithms (Figure S33). This poor clustering of samples from the same genotype might be due to the fact that the algorithms did not return many biclusters containing samples from multiple tissue types (Figure S34), suggesting that between-tissue differences are dominating over between-genotype differences.

Many algorithms also achieved good clustering of genes, as measured by enrichment of biclusters for Reactome pathways (Figure 6 and Figure S35). Since we pre-selected the genes to include in the IMPC dataset using the pathway database, the enrichment scores should be higher than expected on a normal dataset. Thus it is the relative performance between algorithms and normalisation methods rather than absolute performance that is of interest. FABIA, SSLB, nsNMF, MultiCluster and Plaid achieved near perfect scores on multiple versions of the dataset. However, this performance should be considered alongside the fact that Plaid returned on average only four biclusters and that nsNMF returned biclusters with high similarity to each other (Figure S16) and thus many of nsNMF’s biclusters may be enriched for the same small set of Reactome pathways (Figure 6). For example, in a run with $K_{\textit{init}}=200$, $145$ of the $200$ biclusters recovered by nsNMF were enriched for the ‘Metabolism’ pathway ($q < 0.05$) compared to only $39$ of the $188$ biclusters recovered by an SSLB run on the same version of the IMPC dataset.

**Figure 7:**
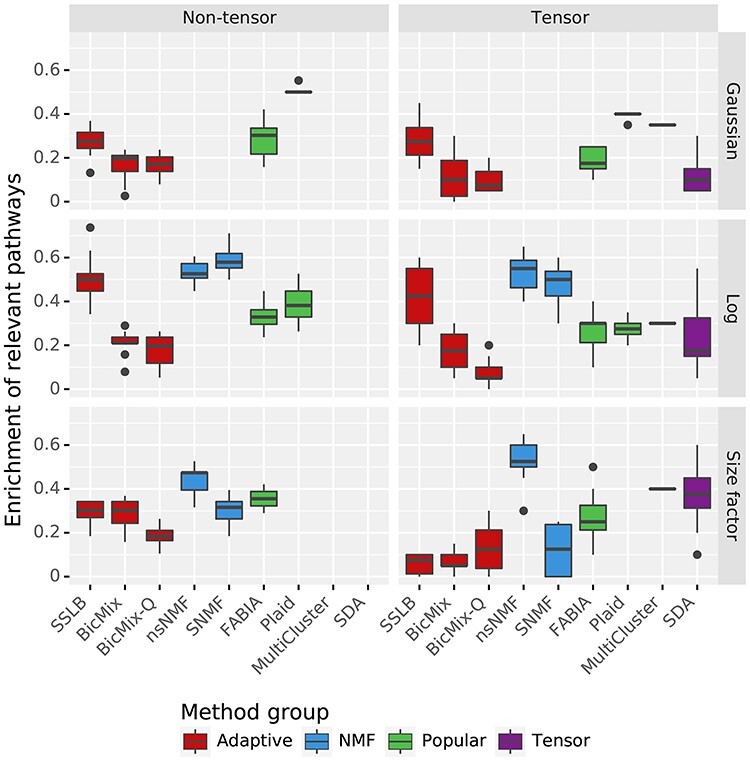
Biclustering ability on IMPC datasets, measured by the mean proportion of knocked-out genes for which the bicluster best matching the samples where the gene was knocked out is enriched for at least one pathway containing the knocked-out gene. This measure is presented for both the tensor and non-tensor form of the dataset, and for the three different normalisation methods described in Section IMPC dataset. Enrichment is measured using the one-tailed hypergeometric test adjusted for multiple testing using the Benjamini–Yekutieli correction, using a threshold for significance of $0.05$. Standard thresholding is applied and $K_{\textit{init}}=50$. Results for $K_{\textit{init}}=200$ are in Figure S36. Note that ‘Tensor’ algorithms could not be run on the non-tensor datasets, ‘NMF’ algorithms could not run on datasets that used quantile normalisation and Plaid failed to run on the dataset that used DESeq’s size factor normalisation (Table S6).

The unifying test on IMPC data is the biclustering ability. We proposed that for each gene that was knocked out, the algorithms should recover a bicluster that contains the samples where this gene was knocked out and that is enriched for the pathways containing this gene. We can thus measure biclustering ability in this dataset by considering the proportion of knockout genotypes for which the algorithm has recovered such a bicluster. We consider the proportion of knockout genotypes for which the bicluster best recovering the samples is enriched for at least one pathway containing the knocked-out gene (Figure 7). To achieve a high score, an algorithm needs to (1) cluster samples well by genotype, (2) cluster genes well by pathway and (3) return biclusters where there is a link between the samples selected and the genes selected. The ‘NMF’ algorithms, Plaid and SSLB did best according to this metric, achieving enrichment of relevant pathways for approximately 45% of the knockout genotypes in multiple versions of the IMPC dataset. Most algorithms had worse performance when using $K_{\textit{init}}=200$ (Figure S36), particularly SNMF that failed on 34 of the 40 runs using $K_{\textit{init}}=200$ (Table S8). Plaid was the only algorithm to fail on a higher percentage of all runs (54 out of 120) but had similar failure rates when using $K_{\textit{init}}=50$ and $K_{\textit{init}}=200$.

**Figure 8:**
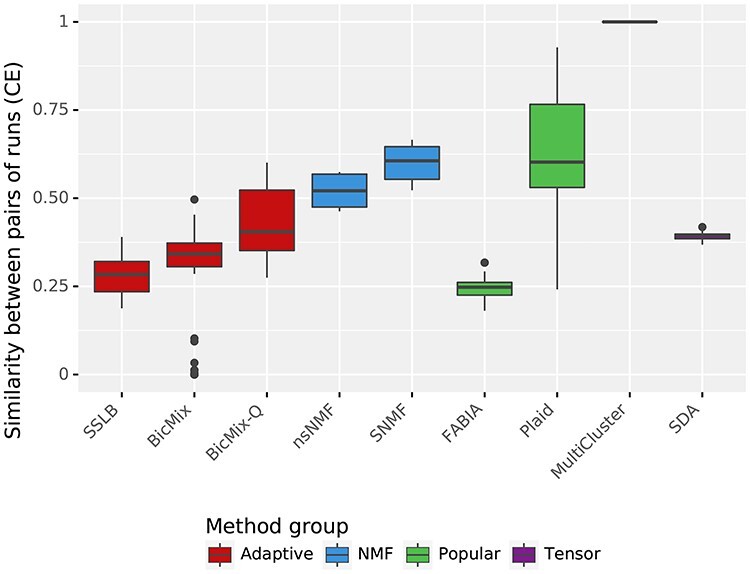
Robustness across random seeds of biclusters recovered by the algorithms. For each algorithm, we consider pairs of runs that differ only in the random seed used and for each such pair calculate the similarity between the set of biclusters recovered in one run and the set of biclusters recovered in the other run. We measure similarity between the sets of biclusters using CE. This boxplot shows the spread of similarity scores achieved by each algorithm. Higher scores are preferred, as they indicate that the algorithm recovers similar biclusters on each re-run. MultiCluster is deterministic, so achieves maximal score of 1. Plaid and the ‘NMF’ algorithms all achieve relatively high scores.

Strikingly, the reconstruction error (NRE) on the IMPC datasets is much worse (higher) than on the simulated datasets (Figure S37), except for nsNMF. This demonstrates the additional complexity in the real datasets compared to the simulated datasets.

### 3.3 Results on sorted blood cell dataset

To investigate whether between-tissue differences always dominate the analysis, we used the Benaroya dataset. This dataset has six cell types that are closely related - they are expected to have gene pathways in common that biclustering methods should detect. Moreover, these six cell types can be grouped into the broader classes of myeloid cells and lymphoid cells (Table S4). We looked in detail at one of the 10 runs by each algorithm on each version of the dataset (Figures S35–S54). We found that most biclusters recovered on the log-transformed dataset were not enriched for any cell type, cell type group, disease state or treatment. However, on the size factor normalised dataset, all algorithms except BicMix-Q and the ‘Popular’ algorithms recovered biclusters that contained mostly lymphoid cell types, or mostly myeloid cell types (Table S9), showing that cell type is still important in blood cell datasets, but that the algorithms can cluster together samples from similar cell types. All algorithms returned multiple biclusters including samples from every cell type, again showing that cell type does not dominate every bicluster in this dataset that has cell types that are more similar to each other.

SSLB, BicMix, nsNMF, MultiCluster and Plaid returned biclusters specific to individual cell types (Table S9 and Figures S46, S47, S48, S49, S54). SSLB, nsNMF and BicMix-Q each recovered a bicluster containing mainly samples from subjects with multiple sclerosis after treatment with IFN-beta and these biclusters all contained samples from multiple cell types (Figures S46, S48 and S50). Aside from a few biclusters containing mainly myeloid samples from subjects with sepsis recovered by SSLB, these IFN-beta biclusters were the only recovered biclusters with clear biological meaning beyond cell type. These biologically interesting biclusters were found only in size factor normalised datasets.

Overall, more interesting biclusters were recovered on the dataset that used size factor normalisation than on the log-transformed dataset. The ‘NMF’ and ‘Adaptive’ algorithms seem to have best found biologically interesting biclusters in this sorted blood cell dataset.

### 3.4 Robustness between runs

With the exception of MultiCluster, the algorithms compared here are stochastic and thus may produce different results each time they are run. If a similar set of biclusters is recovered by repeated runs of an algorithm, this can give confidence that the bicluster decomposition is meaningful and that the algorithm is robust. Thus we felt it important to quantify how similar the results from a run were to the results when using a different random seed, as a measure of robustness of an algorithm. For each algorithm, in turn, we considered pairs of runs on the same dataset that differed only in random seed used (i.e. they ran on the same form of the IMPC dataset, with the same parameters and using the same value of $K_{\textit{init}}$) and calculated the similarity between the two biclusterings recovered. We measured similarity between the two biclusterings using CE. As MultiCluster is deterministic, it achieves a perfect score of $1$ in this test. Of the remaining algorithms, Plaid and the ‘NMF’ algorithms are the only ones to have a median similarity score between runs of over $0.5$ (Figure 8).

### 3.5 Computational time

It is important to evalute the time taken for the algorithms to run, and how this scales with the size and complexity of the dataset, as this can restrict the datasets that an algorithm is able to process. The slowest algorithm on the IMPC datasets was SNMF, which took 8 h to run on the tensor log-transformed dataset, compared to the 7 s taken by nsNMF (Table 4). Figure S55 shows runtime with small value of $K_{\textit{init}}$, Figure S56, and Figure S57 show, for simulated datasets and IMPC datasets respectively, that runtime for the ‘Adaptive’ algorithms, SDA, SNMF and FABIA changed drastically with $K_{\textit{init}}$.

## 4 Discussion

We highlight two key contributions we hope will be of use to future comparison studies and users of biclustering algorithms. Firstly, we introduced a thresholding procedure that enhances the sparsity of returned biclusters, aiding analysis. The raw output of algorithms such as nsNMF was difficult to analyse, but after applying the thresholding procedure nsNMF was one of the top-ranking algorithms. Secondly, we introduced two metrics, MBR and NRE, which can be used to judge the results of algorithms on datasets where the truth is not known.

On simulated datasets ‘Adaptive’ algorithms had the best overall performance. We investigated the limitations of biclustering algorithms by varying dataset complexity. All algorithms were relatively unaffected by increasing noise in simulated datasets, but performance decreased when dataset size and number of biclusters were increased. Dense biclusters were generally recovered better than sparse biclusters. From a biological perspective, we expect the dense biclusters to correspond to confounding variables such as sex and age and the sparse biclusters to be more biologically interesting, so this behaviour is not ideal, although it is to be expected since sparse biclusters contain less signal than dense biclusters.

Like previous studies, we found that algorithms achieved good enrichment of biclusters for gene pathways in real datasets. We carefully chose a knockout mouse dataset to allow evaluation of biclustering on real datasets, a task that has eluded previous studies, and found that ‘NMF’ algorithms, SSLB and Plaid performed best at recovering biclusters. In the sorted blood cell dataset, the ‘Adaptive’ algorithms and nsNMF best recovered biologically relevant biclusters.

In terms of ease of use, ‘Adaptive’ algorithms and Plaid are the only algorithms well suited to use without tuning of $K_{\textit{init}}$, and also did not require post-processing. ‘NMF’ algorithms and Plaid had the most robust results, with different runs having on average a similarity of $0.5$ to each other, as measured by CE. Plaid and nsNMF were the fastest, with nsNMF taking 7 seconds on the largest IMPC dataset, compared to the 8 h taken by the slowest algorithm (SNMF). We found that most algorithms performed well with default parameters, with few parameter values that showed consistent and significant improvement over default values during our parameter sweep.

We found that normalisation method of real data had a large impact on the performance of algorithms. On the Benaroya dataset, more biologically interesting biclusters were found when using size factor normalisation and no method performed better on the log-transformed dataset. On the IMPC dataset, the relative ranking of normalisation methods varied considerably between algorithms. When we compared the performance of algorithms on simulated datasets that used shift-scale patterns, we found that SSLB in particular performed better on ‘shift’ dataset than the ‘scale’ dataset. Thus, if a dataset is believed to be driven by scale patterns, we recommend applying a log transformation to transform the pattern to a shift pattern.

Overall, many algorithms performed better than the ‘Popular’ algorithms that had performed best in previous reviews, showing the need for continued comparison studies as biclustering algorithms develop further. ‘NMF’ algorithms had poor raw output but nsNMF was one of the top-ranking algorithms after using the sparsity-inducing thresholding procedure we introduce. ‘Tensor’ algorithms did not perform better than other algorithm types, despite both real and simulated datasets having tensor structure, with all expected factors having tricluster structure. ‘Adaptive’ algorithms performed particularly well on the simulated datasets, and SSLB also had good performance on the real datasets.

**Key Points**

- We introduce a promising thresholding procedure to enhance sparsity of the returned biclusters, essential for FABIA, SDA and ‘NMF’ algorithms that otherwise returned only biclusters containing every gene and every sample.
- We introduce the MBR metric for redundancy within a run, and NRE metric for measuring reconstruction error, which can be used even when the true structure of the dataset is not known.
- We have shown the potential for re-purposing of ‘NMF’ algorithms to the task of biclustering. The nsNMF algorithm was orders of magnitude faster than the more complex algorithms and had good performance, particularly on the real datasets.
- For datasets with unknown structure we recommend SSLB. If a fast algorithm is needed and the number of biclusters is known, or if metrics are available to aid the choice of $K_{\textit{init}}$, then we recommend nsNMF.

## Supplementary Material

Supplementary_bbab140

## Notes

**Chris Wallace** is a Director of Research at the Cambridge Institute of Therapeutic Immunology and Infectious Disease and the MRC Biostatistics Unit, University of Cambridge. She works on integrative analysis of genomic data.

**Kath Nicholls** is a PhD student in Mathematics Genomics and Medicine at the University of Cambridge. She is interested in genomic analysis of the immune system.
