# Supplementary material for: Comparison of sparse biclustering algorithms for gene expression datasets
Source: Brief Bioinform. 2021 May 6;22(6):bbab140. doi: 10.1093/bib/bbab140 (PMC8574648; doi:10.1093/bib/bbab140)
Supplement: Supplementary_bbab140 [file supplementary_bbab140.zip › Supplementary.pdf]

Contents

S1 Algorithms compared **5**
S1.1 Plaid . . . 5
S1.2 Sparsity-inducing priors . . . 5
S1.2.1 FABIA . . . 6
S1.2.2 BicMix . . . 6
S1.2.3 SSLB . . . 7
S1.2.4 SDA . . . 8
S1.3 Non-negative matrix factorisation . . . 8
S1.3.1 SNMF . . . 8
S1.3.2 nsNMF . . . 9
S1.4 MultiCluster . . . 9

S2 Parameter sweep **10**
S2.1 BicMix . . . 10
S2.1.1 Prior . . . 10
S2.1.2 Pre-processing . . . 12
S2.2 FABIA . . . 13
S2.2.1 Sparsity . . . 13
S2.2.2 Rescaling during fitting . . . 13
S2.2.3 Bicluster membership . . . 13
S2.3 Plaid . . . 14
S2.4 MultiCluster . . . 14
S2.5 SDA . . . 14
S2.5.1 Prior . . . 14
S2.5.2 Convergence . . . 16
S2.6 nsNMF . . . 16
S2.7 SNMF . . . 16
S2.8 SSLB . . . 20
S2.8.1 Prior for B . . . 20
S2.8.2 Prior for X . . . 20

S3 Evaluation Metrics **22**
S3.1 Accuracy on simulated datasets . . . 22
S3.1.1 Metrics comparing two biclusterings . . . 22
S3.1.2 Clustering error . . . 23

S4 Simulated datasets **24**

S5 Benaroya dataset **27**

S6 Post-processing **27**

S7 Extra results **37**

List of Tables

S1 Datasets used in the parameter sweep. 10
S2 Effect of parameters on Clustering Error (CE). 11
S3 Summary of simulated datasets. The attributes of the datasets are displayed in bold if they differ from the base dataset. N is the number of samples, T the number of tissues and G the number of genes in the dataset. 25
S4 Number of samples in Benaroya RNA-seq dataset for each cell type and disease. 27
S5 Number of failed runs by each algorithm on each simulated dataset, restricted to runs with the value of K_{init} chosen for analysis. 38
S6 Number of failed runs by each algorithm on each IMPC dataset, restricted to runs with $K_{init} = 50$. 39
S7 Number of failed runs by each algorithm on each simulated dataset. 40
S8 Number of failed runs by each algorithm on each IMPC dataset. 41
S9 Summary of results on the Benaroya RNA-seq dataset by algorithm and normalisation method. 60

List of Figures

S1 Parameter sweep for BicMix. 12
S2 Parameter sweep for BicMix without qnorm. 13
S3 Parameter sweep for FABIA. 14
S4 Parameter sweep for FABIA with $spz = 1.5$. 15
S5 Parameter sweep for Plaid. 15
S6 Parameter sweep for SDA. 16
S7 Running time of SDA with different *conv_crit* values. 17
S8 Parameter sweep for nsNMF. 18
S9 Parameter sweep for SNMF. 19
S10 Parameter sweep for SSLB. 21
S11 Illustration of simulated datasets. 24
S12 Overlap between biclusters in simulated datasets and the effect this overlap has on performance. 26
S13 Number of genes contained in biclusters, with no thresholding applied. . . . 29
S14 Number of samples contained in biclusters, with no thresholding applied. . . 30
S15 Biclustering accuracy (CE) against the threshold for inclusion in bicluster. . 31
S16 Average similarity of biclusters (MBR) within a run plotted against the threshold for inclusion in bicluster, for IMPC datasets. 32
S17 Normalised reconstruction error on simulated datasets, plotted against the threshold for inclusion in bicluster. 33
S18 Biclustering performance on IMPC datasets, measured by the mean proportion of knocked-out genes for which the biclusters best matching the samples where the gene was knocked out is enriched for at least one pathway containing the knocked-out gene. 34

S19 Number of genes contained in biclusters, with thresholding applied as specified in Section S6. . . . 35
S20 Number of samples contained in biclusters, with thresholding applied as specified in Section S6. . . . 36
S21 Recovery of K. . . . 42
S22 Biclustering accuracy (CE) of algorithms plotted against K_{init}. . . . 43
S23 Robustness to choice of K_{init}, shown by final value of K (after post-processing) plotted against initial value of K. . . . 44
S24 Biclustering accuracy (CE) of algorithms across datasets with a range of sizes. 45
S25 Biclustering accuracy (CE) of algorithms across datasets with different numbers of biclusters. . . . 46
S26 Biclustering accuracy (CE) of algorithms across datasets with different sparsity of biclusters. . . . 47
S27 Recovery of true biclusters, grouped by size of true bicluster (proportion of total area taken up by true bicluster). . . . 48
S28 Relevance of returned biclusters, grouped by size of returned bicluster (proportion of total area taken up by returned bicluster). . . . 49
S29 Biclustering accuracy (CE) of algorithms across datasets with different noise distribution. . . . 50
S30 Biclustering accuracy (CE) of algorithms across datasets using different models for bicluster values. . . . 51
S31 Recovery of K when algorithms are started with the value of K_{init} chosen for the study ($K_{\text{init}} = K$ for all algorithms except the *Adaptive* algorithms which use $K_{init} = K + 10$). . . . 52
S32 Sample clustering ability on IMPC datasets, measured by the mean over the tissue traits of the maximum F_1 score achieved by a bicluster. . . . 53
S33 Sample clustering ability on IMPC datasets, measured by the mean over the genotype traits of the maximum F_1 score achieved by a bicluster. . . . 54
S34 Proportion of bicluster taken up by the largest tissue contained in the bicluster, indicating how frequently the algorithms return biclusters containing multiple tissues. . . . 55
S35 Gene clustering ability on IMPC datasets. . . . 56
S36 Biclustering ability on IMPC datasets. . . . 57
S37 Normalised reconstruction error (NRE) across all datasets. . . . 58
S38 Benaroya summary - SSLB, **log** transformation. . . . 59
S39 Benaroya summary - BicMix, **log** transformation. . . . 61
S40 Benaroya summary - nsNMF, **log** transformation. . . . 62
S41 Benaroya summary - MultiCluster, **log** transformation. . . . 63
S42 Benaroya summary - SDA, **log** transformation. . . . 64
S43 Benaroya summary - SNMF, **log** transformation. . . . 65
S44 Benaroya summary - FABIA, **log** transformation. . . . 66
S45 Benaroya summary - Plaid, **log** transformation. . . . 67
S46 Benaroya summary - SSLB, **size factor** normalisation. . . . 68
S47 Benaroya summary - BicMix, **size factor** normalisation. . . . 69
S48 Benaroya summary - nsNMF, **size factor** normalisation. . . . 70

S49 Benaroya summary - MultiCluster, **size factor** normalisation. 71
S50 Benaroya summary - BicMix-Q, **size factor** normalisation. 72
S51 Benaroya summary - SDA, **size factor** normalisation. 73
S52 Benaroya summary - SNMF, **size factor** normalisation. 74
S53 Benaroya summary - FABIA, **size factor** normalisation. 75
S54 Benaroya summary - Plaid, **size factor** normalisation. 76
S55 Mean run time across large datasets (log scale). 77
S56 Effect of K_{init} on run time. 78
S57 Effect of K_{init} on run time. 79

S1 Algorithms compared

Most algorithms included in this comparison use the same form, differing primarily in whether they explicitly allow sharing of information across cell types and in which approach they use to induce sparsity in the biclusters. We define a matrix $Y \in \mathbb{R}^{n \times p}$ where entry Y_{ij} gives the expression of gene j in sample i. This can either be raw read counts from an RNA-seq experiment, or normalised counts which have been adjusted for sample-specific effects such as library size, or gene-specific effects such as mean expression level. The typical approach to biclustering is to factorise this matrix as a product of two matrices $X \in \mathbb{R}^{n \times K}$, which we call the sample loadings matrix, and $B \in \mathbb{R}^{p \times K}$, which we call the gene loadings matrix, with error matrix ε:

$$Y = XB^T + \varepsilon \tag{1}$$

S1.1 Plaid

The general Plaid model is:

$$Y_{ij} = \mu_0 + \sum_{k=1}^{K} \left(\mu_k + \alpha_{jk} + \beta_{ik}\right) \rho_{jk} \kappa_{ik} + \varepsilon_{ij} \tag{2}$$

The gene expression matrix is written as a sum of K layers, each of which is a sum of a layer effect μ_k, gene-specific effects α_{jk} for each gene and sample-specific effects β_{ik} for each sample. The membership of genes and samples to each layer is given by binary variables ρ_{jk} and κ_{ik} respectively. Finally, there is a background layer with constant value μ_0. The error for each entry is given by ε_{ij}. Layers are added one-at-a-time and parameters are chosen by alternating ordinary least squares.

S1.2 Sparsity-inducing priors

Sparse matrix factorisation algorithms are a type of regularised optimisation problem: we want to minimise the reconstruction error $(Y - XB)$ whilst also restricting the number of non-zero elements in the matrices. We explain how to see this problem in a Bayesian framework through two simpler examples of regularised optimisation, which each estimate a vector of coefficients β instead of a matrix B. The first is linear regression with L_1-penalty (called the *LASSO*):

$$\hat{\beta} = \arg\min_{\beta} \left\{ \|\mathbf{X}\beta - \mathbf{Y}\|_2^2 + \lambda \|\beta\|_1 \right\} \tag{3}$$

and the second is linear regression with L_2-penalty (called *ridge regression*):

$$\hat{\beta} = \arg\min_{\beta} \left\{ \|\mathbf{X}\beta - \mathbf{Y}\|_2^2 + \lambda \|\beta\|_2^2 \right\} \tag{4}$$

where the L_1-norm is $\|Z\|_1 = \sum_i |Z_i|$, the L_2-norm is $\|Z\|_2^2 = \sum_i Z_i^2$, and λ determines the strength of the sparsity constraint. In both cases, larger λ will lead to a sparser model.

Such regularised optimisation problems can often be written in a Bayesian framework. For example, the ridge regression estimator is the maximum a posteriori (MAP) estimate

of β when the data are assumed to be generated with Gaussian noise around $\mathbf{X}\beta$ and a Gaussian prior $\mathcal{N}(\mathbf{0}, \lambda I)$ is assumed for β:

$$p(\beta|\lambda) = \frac{1}{\sqrt{(2\pi\lambda)^k}} \exp\left(-\frac{\|\beta\|_2^2}{2\lambda}\right) \tag{5}$$

The LASSO estimator is similar, with a Laplacian prior instead:

$$p(\beta|\lambda) = \prod_{j=1}^{p} \frac{\lambda}{2} \exp\left\{-\lambda\left|\beta_j\right|\right\} = \left(\frac{\lambda}{2}\right)^k \exp\left\{-\lambda\|\beta\|_1\right\} \tag{6}$$

Many of the algorithms included in this study use this Bayesian framework and use a prior on the entries of the matrices X and B. The priors used often encourage many entries of these matrices to be 0 and so they are called sparsity-inducing priors.

S1.2.1 FABIA

Factor Analysis for Bicluster Acquisition (FABIA) [1] is a sparse factor analysis model using the model given in Equation 1. FABIA induces sparsity by using a Laplacian prior on all entries of X and B. The same prior is used for all entries in the matrix.

In the parameter sweep conducted before full analysis, we found that using higher sparsity by setting spz = 1.5 greatly improved FABIA's performance.

S1.2.2 BicMix

BicMix [2] uses the model given in Equation 1 and allows each bicluster to be either *'sparse'* or *'dense'*. On all 'sparse' biclusters, BicMix encourages sparsity through three layers of shrinkage, each of which uses a three parameter beta (TPB) prior [3].

One parameter that drastically affected performance was whether the data was quantile-normalised before BicMix ran. Since the effect was substantial but inconsistent, we ran BicMix twice on each dataset: once with quantile-normalisation (BicMix-Q) and once without (BicMix).

The full BicMix model for the gene loading matrix $B \in \mathbb{R}^{GxK}$ uses three levels of shrinkage using Three Parameter Beta distributions:

$$\begin{aligned} \rho &\sim \mathcal{TPB}(e, f, \nu) \\ \zeta_k &\sim \mathcal{TPB}\left(c, d, \frac{1}{\rho} - 1\right) \\ \varphi_{j,k} &\sim \mathcal{TPB}\left(a, b, \frac{1}{\zeta_k} - 1\right) \\ B_{j,k} &\sim \mathcal{N}\left(0, \frac{1}{\varphi_{j,k}} - 1\right) \end{aligned} \tag{7}$$

where the parameters are usually set to $a = b = c = d = e = f = 0.5, \nu = 1$. A similar model is used for the entries of X.

The Three Parameter Beta distribution [3] is a generalised version of a Beta distribution, and certain parameterisations yield named priors such as the Horseshoe prior ($a = b = 0.5, \phi = 1$) [4]. The probability density function is:

$$f(x : a, b, \phi) = \frac{\Gamma(a+b)}{\Gamma(a)\Gamma(b)} \phi^b x^{b-1} (1-x)^{a-1} \{1 + (\phi - 1)x\}^{-(a+b)} \tag{8}$$

S1.2.3 SSLB

Spike-and-Slab Lasso Biclustering (SSLB) [5] is another Bayesian biclustering algorithm using the model given in Equation 1. SSLB uses the Spike-and-Slab Lasso prior [6] on both the factor matrix X and the loadings matrix B. The Spike-and-Slab Lasso prior is a mixture of two Laplacians, one of which has weak regularisation (the *slab*) and one has strong regularisation (the *spike*). When fitting a model, stronger regularisation will result in a sparser result but may come at the expense of accuracy. Using this prior allows much stronger regularisation on the coefficients that are near-zero (those in the spike) in order to achieve sparsity, whilst having weaker regularisation on the larger coefficients (those in the slab) to avoid loss of accuracy. Moran et al. claim that their algorithm produces sparser matrices than FABIA and BicMix.

Another advantage of the SSLB prior compared to FABIA and BicMix is that it allows different levels of sparsity for each bicluster. FABIA uses the same prior for all biclusters, and BicMix only allows for two different levels of sparsity ('sparse' or 'dense'). SSLB allows each bicluster to have a different sparsity parameter.

Model for B_{jk} The basic model for B_{jk} is:

$$\begin{aligned} p(B_{jk}) &= 1(\text{In slab}) \quad p_{slab}(B_{jk}) + 1(\text{In spike}) \quad p_{spike}(B_{jk}) \\ &= \gamma_{jk} \qquad\qquad \psi(B_{jk}|\lambda_1) + (1 - \gamma_{jk}) \qquad \psi(B_{jk}|\lambda_0) \end{aligned} \tag{9}$$

Here γ_{jk} indicates whether or not variable j is contained in bicluster k. If so, $\gamma_{jk} = 1$ and the factor value B_{jk} is chosen from the *slab* which is a Laplace(λ_1) distribution. If not, $\gamma_{jk} = 0$ and the factor value is chosen from the *spike* which is a Laplace(λ_0) distribution.

The Laplacian distribution is $\psi(x|\lambda) = \frac{\lambda}{2} e^{-\lambda|x|}$.

The final layer of complexity is that the cluster membership variables γ_{jk} are modelled using a Beta-Binomial prior:

$$\begin{aligned} \gamma_{jk}|\theta_k &\sim \text{Bernoulli}\,(\theta_k) \\ \theta_k &\sim \text{Beta}(a, b) \end{aligned} \tag{10}$$

Model for X_{ik} The model for X_{ik} is more complicated, with three different priors for the sparsity parameters $\tilde{\theta}_k$ possible.

The simplest prior is the same as that used for B_{jk}:

$$\tilde{\theta}_k \sim \text{Beta}(\tilde{a}, \tilde{b}) \tag{11}$$

The default prior is the Indian Buffet Process (IBP) prior [7, 8] which forces each bicluster to contain fewer samples than the previous bicluster. The SSLB implementation also allows

for use of the Pitman-Yor extension to the IBP prior, which forces the sparsity weights $\tilde{\theta}_k$ to shrink more drastically[1]. In the parameter sweep S2, we considered the effect of changing the prior and found that it did not have significant impact on performance.

S1.2.4 SDA

In contrast to the matrix algorithms seen so far, Sparse Decomposition of Arrays (SDA) [9] rearranges the gene expression matrix to be a 3D tensor with dimensions $m \times p \times t$ where the total number of samples $n = mt$ is the product of the number of individuals m and number of tissues t. It decomposes the gene expression matrix Y as a tensor product of three matrices A, B, Z corresponding to individual, gene and tissue components respectively:

$$Y = \sum_k a_k \otimes b_k \otimes z_k + \varepsilon \tag{12}$$

This enables information to be shared between different tissues, which the other algorithms are unable to do. This decomposition can be written as a sum of contributions from each bicluster:

$$Y_{ijl} = \sum_k A_{ik} B_{jk} Z_{lk} + \varepsilon_{ijl} \tag{13}$$

SDA encourages sparsity in the gene components by using a prior similar to that of SSLB. It uses the Gaussian Spike-and-Slab prior described in [10], which consists of a point-mass at 0 (the *spike*) and a Gaussian distribution (the *slab*). It does not encourage sparsity in either sample components or tissue components.

The implementation of SDA that we use provides an alternative model for when $t = 1$, but we have chosen not to include it in this comparison as the tensor factorisation seems the most promising.

S1.3 Non-negative matrix factorisation

Non-negative matrix factorisation algorithms factorise the original matrix as $Y = XB$, where the entries of X and of B are non-negative. Enforcing non-negativity of the matrices involved in a product $Y = XB$ has two main advantages. Firstly, it makes this product easier to interpret as the effects from different biclusters are simply added together, with no possibility for the effects to cancel each other out. Secondly, it naturally encourages sparsity as it restricts the number of biclusters that can have a large contribution to each value in the matrix. This effect on sparsity isn't that substantial, so many of the algorithms use additional techniques to encourage sparsity.

S1.3.1 SNMF

Sparse non-negative matrix factorisation (SNMF) [11] factorises the gene expression matrix Y as a product of two non-negative matrices X and B. Non-negativity makes the biclusters easier to interpret, and naturally encourages sparsity. SNMF uses an L_1 penalty on the

[1] Formally the expected values of these parameters are forced to follow a $O\left(k^{-1/d}\right)$ power law

elements of the gene loadings matrix B to further encourage sparsity of this matrix. The paper also includes a algorithm that uses an L_1 penalty on the sample loadings matrix instead, but we chose to use the version with sparsity on the gene loadings matrix as one of the main aims is to find interesting groups of genes, and these are easier to interpret if they are sparser.

S1.3.2 nsNMF

Like SNMF, the non-smooth non-negative matrix factorisation (nsNMF) model [12] factorises the gene expression matrix as a product of non-negative matrices:

$$Y = XSB \tag{14}$$

The key difference is the inclusion of a third matrix, called the *smoothing matrix*:

$$\mathbf{S} = (1-\theta)\mathbf{I} + \frac{\theta}{K}\mathbf{1}\mathbf{1}^T \tag{15}$$

When $\theta = 0$, we have $S = I$ which makes nsNMF equivalent to normal non-negative matrix factorisation. However, for larger θ, the matrix S helps to increase sparsity without increasing error. Without it, increasing sparsity of samples loading matrix X would encourage the genes loading matrix B to be denser in order for the product XB to be close to the original matrix Y. Thus if we want sparsity in both X and B, we will suffer an increase in error. With nsNMF, $Y = (XS)B$ so in order to allow B to be sparse, we must make sure that XS is not too sparse. When a matrix is multiplied by S, it becomes less sparse. Thus we can encourage X to be sparse, and XS will not be that sparse, meaning that B will not be forced to be denser. Thus, with nsNMF we can apply sparsity constraints to B as well as to X without a large increase in the error.

S1.4 MultiCluster

MultiCluster [13], like SDA, rearranges the gene expression matrix into a 3D tensor and decomposes it as:

$$Y = \sum_k a_k \otimes b_k \otimes z_k + \varepsilon \tag{16}$$

To aid interpretability and encourage sparsity, the tissue components z_k are constrained to be non-negative. MultiCluster uses a deterministic algorithm to find optimal components a_k, b_k, z_k for each k in turn.

Table S1: Datasets used in the parameter sweep.

Name	Individuals	Tissues	Genes	Bicluster sizes	K	Noise
negbin_K20	10	10	1000	mixed	20	NegBin
gaussian_K50	10	10	1000	small	50	Gaussian
no_noise_K10	10	10	1000	large	10	No noise

S2 Parameter sweep

Complex algorithms such as those included in this review can be very sensitive to choice of parameters. This has two consequences for this review: firstly we need to decide how to set the parameters during the review, and secondly we should consider the robustness of algorithms to parameter choice in itself as a criterion by which to judge the algorithms.

Many previous reviews of biclustering algorithms have used a single set of parameters throughout the study [14, 15]. By taking this approach, the comparison study naturally favours the algorithms which have good default parameters, or which do not vary much depending on the parameter choice. Another approach is to try many different combinations of parameters for each algorithm on each dataset. In a review of module detection algorithms, Saelens et al. performed a grid search for each algorithm on each dataset to find an optimal set of parameters [16] for each dataset. This approach of finding optimal parameter sets can be computationally expensive and also does not extend well to real datasets, where calculating a score for results is more difficult, as the true structure of the dataset is unknown.

We conducted a investigation of the effect of changing key parameters for each algorithm, looking for parameter values that consistently improved performance over the default parameters on three distinct simulated datasets. Overall we found that most parameters had little effect on performance as measured by clustering error (CE) and for most parameters, there was either no clear optimal parameter, or the default parameter was clearly optimal. The results are summarised in Table S2.

We simulated three very differently structured datasets (Table S1), and ran each algorithm with five random seeds using $K_{init} = 20$ and five seeds using $K_{init} = 50$ for each set of parameter values on each dataset. For each parameter (except K) we chose approximately five different values, including the default value. We then ran the algorithm changing each parameter in turn, keeping the other parameters at their default value, running 5 times with $K = 20$ and 5 times with $K = 50$. Below we discuss for each algorithm how we chose the parameters to change, and show the effect of changing each parameter whilst keeping the other parameters at their default value.

S2.1 BicMix

S2.1.1 Prior

BicMix uses a Three Parameter Beta prior to induce sparsity in the factor values. There are two key parameters that control this prior, with default values $a = b = 0.5$. This choice of a, b is called the Horseshoe Prior. [3] discusses hyper-parameters for the Three Parameter Beta distribution in sparse models, and note that it is necessary to have $a < 1$ in order to

Table S2: Effect of parameters on Clustering Error (CE). For each parameter we consider if changing it had an effect on performance and if so whether the effect was consistent and substantial. Finally we note the action we decided to take, leaving this column blank if the default value is used.

Method	Parameter	Effect?	Consistent?	Substantial?	Action
BicMix	a	**Yes**	No	No	
	b	**Yes**	No	No	
	qnorm	**Yes**	No	**Yes**	Run with both
FABIA	alpha	No			
	spz	**Yes**	**Yes**	**Yes**	Use spz=1.5
	spl	No			
	eps	**Yes**	**Yes**	**Yes**	None, fixed with spz=1.5
	rescale_l	**Yes** (failure)			
Plaid	row_release	**Yes**	No	**Yes**	
	col_release	**Yes**	No	No	
SDA	num_dense	No			
	step_size	**Yes**	**Yes**	No	
	conv_crit	No			
nsNMF	θ	**Yes**	**Yes**	No	
SNMF	β	**Yes**	No	No	
SSLB	d	No			
	IBP	No			
	alpha	No			
	a	No			
	b	No			

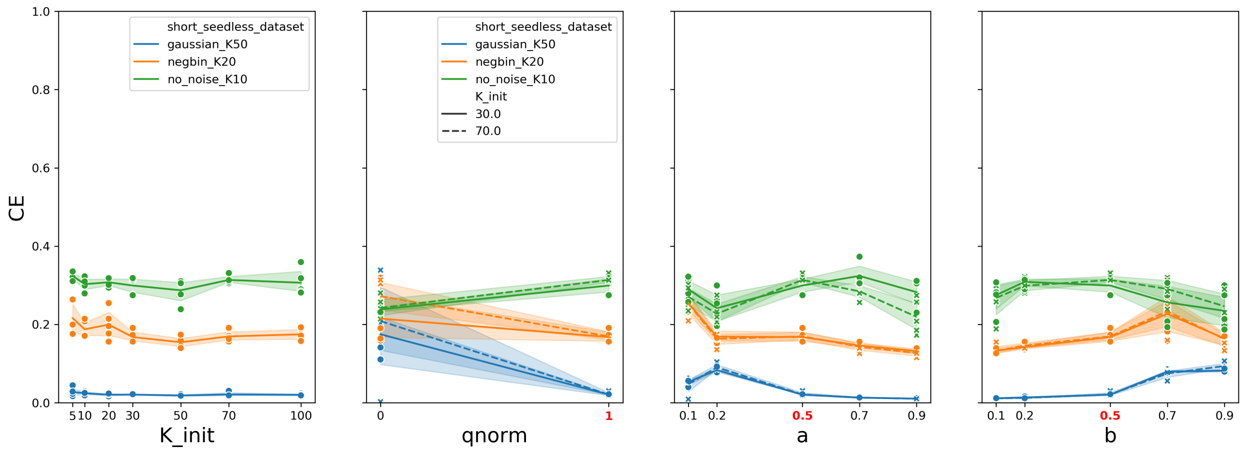

Figure S1: Parameter sweep for BicMix. Each plot shows the effect on the clustering error measure (CE) when a single parameter is varied, while the other parameters are kept at their default values. The default value for a parameter is highlighted in red on the x-axis. Higher clustering error values are better.

achieve sparse solutions, and suggest using $b < 1$ too. We used this to guide our choice of values to try for a, b.

The performance is affected by these parameters, but the optimal parameter choice isn't the same for all datasets (Figure S1).

S2.1.2 Pre-processing

The other important parameter is *qnorm*, which determines whether or not a Gaussian rank transformation is applied to each gene in the data matrix before BicMix runs.

Gaussian rank transformation transforms each variable (gene) so that the resulting distribution is exactly Gaussian. The values from the original variable are ranked with ties broken at random, and the value at position k in the list of n values will be mapped to the $\frac{k}{n+1}$ quantile of the standard Gaussian $N(0,1)$. A key consequence of this is that the many 0s will get mapped to *different* values, so this introduces noise to the data.

Changing *qnorm* has a dramatic effect on performance (Figure S1). Most notably, the performance on the dataset *gaussian_K50* is near 0 when the data are quantile-normalised.

Due to the large changes in performance with quantile-normalisation, we ran two versions of BicMix: one with quantile-normalisation and one without. To check that the default prior parameters are sufficient when quantile-normalisation is not used, we performed another parameter sweep with $qnorm = 0$ and found that there is no parameter choice that shows consistent improvement over the default choice $a = b = 0.5$ (Figure S2).

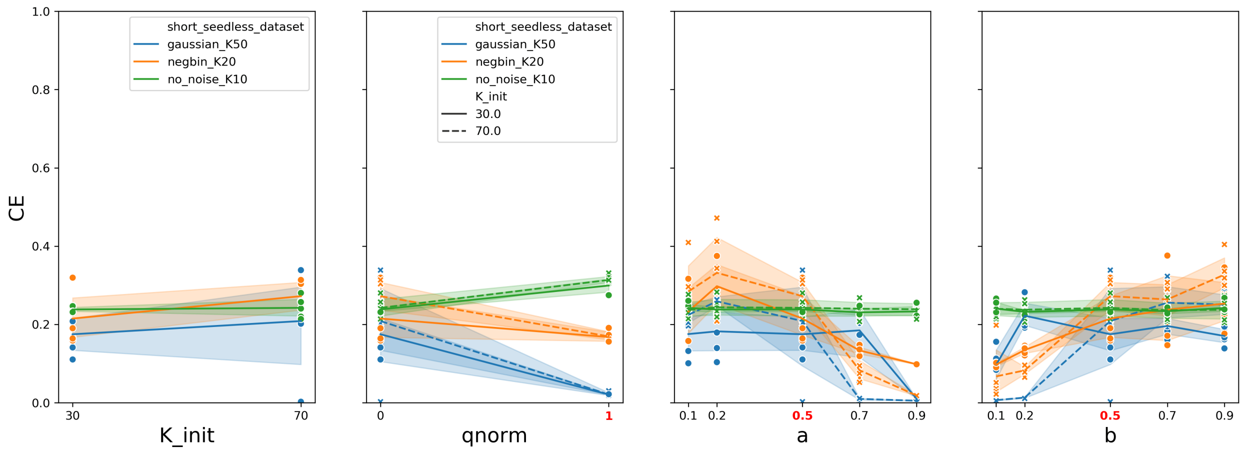

Figure S2: Parameter sweep for BicMix without qnorm. Each plot shows the effect on the clustering error measure (CE) when a single parameter is varied, while the other parameters are kept at their default values (with the exception of qnorm, which is kept at 0 for the parameter sweeps for all other parameters). The default value for a parameter is highlighted in red on the x-axis. Higher clustering error values are better.

S2.2 FABIA

S2.2.1 Sparsity

FABIA controls the sparsity of the factor matrix X using *spz*. The sparsity of the loadings matrix B is controlled using α and *spl*. For all these parameters, higher values increase sparsity. The parameter *eps* determines a threshold under which entries in either X or B will be treated as 0, so higher values increase sparsity of both X and B. The *pyfabia* implementation gives ranges for each value, which we used to guide our choice of values.

The two that seem to have the biggest effect are *eps* and *spz* (Figure S3). The best performance was achieved with $spz = 1.5$ for all datasets. Since the change is substantial and consistent, we chose to use this value of the parameter for the rest of the study. We investigated the effect of changing *eps* when $spz = 1.5$ and found that it still had an effect, but it was not as great as when $spz = 0.5$ (Figure S4).

S2.2.2 Rescaling during fitting

If *rescale_l* is *True* then FABIA rescales each B_k to have unit variance each iteration. This is not the default behaviour, but the *pyfabia* documentation claims this can help convergence, but we found that many runs using this rescaling actually failed to return a result.

S2.2.3 Bicluster membership

After fitting the model, both the *pyfabia* and R FABIA implementations provide functions to determine which rows and columns belong to each bicluster. Values under the threshold are set to 0, essentially removing this element from the bicluster. The threshold is *thresZ* for X and *thresL* for B. These are the only two parameters varied in [16], a review of

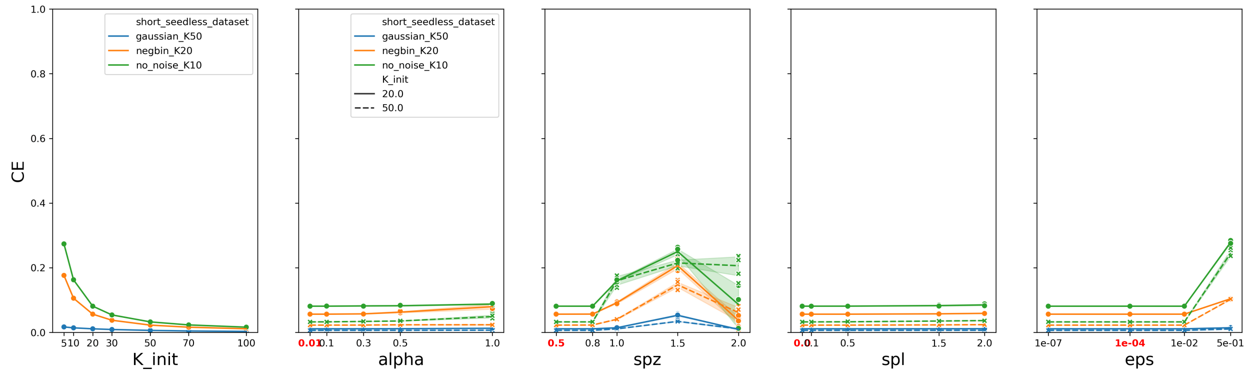

Figure S3: Parameter sweep for FABIA. Each plot shows the effect on the clustering error measure (CE) when a single parameter is varied, while the other parameters are kept at their default values. The default value for a parameter is highlighted in red on the x-axis. Higher clustering error values are better.

module detection algorithms. This thresholding is a post-processing step which we apply to all algorithms, so we ignored this parameter.

S2.3 Plaid

In a Bacherlor's thesis focusing on the Plaid algorithm, Pfundstein notes that the two key paramters to tune are *row_release* and *col_release*, which determine when a row or column is removed from a bicluster [17]. These are also the only two parameters altered in the module detection review [16]. A small recommended range of $[0.5, 0.7]$ is given in the documentation of the *biclust* package that contains the Plaid implementation we used.

Changing these parameters has some effect, but the effect differs for each dataset (Figure S5). Since we are primarily using Plaid as a benchmark to allow results in this study to be related to other reviews of biclustering algorithms, and since these reviews mostly used the default parameters, we did the same.

S2.4 MultiCluster

The implementation of MultiCluster that we use doesn't have any parameters that can be changed.

S2.5 SDA

S2.5.1 Prior

The parameters determining the priors are set up so that the priors are uninformative, so we didn't alter them.

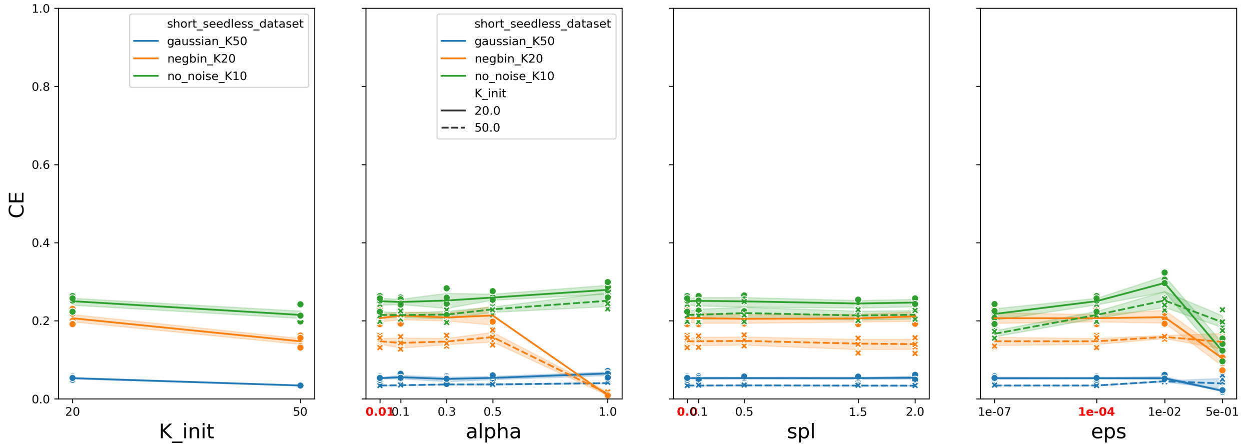

Figure S4: Parameter sweep for FABIA with $spz = 1.5$. Each plot shows the effect on the clustering error measure (CE) when a single parameter is varied, while the other parameters are kept at their default values. The default value for a parameter is highlighted in red on the x-axis. Higher clustering error values are better.

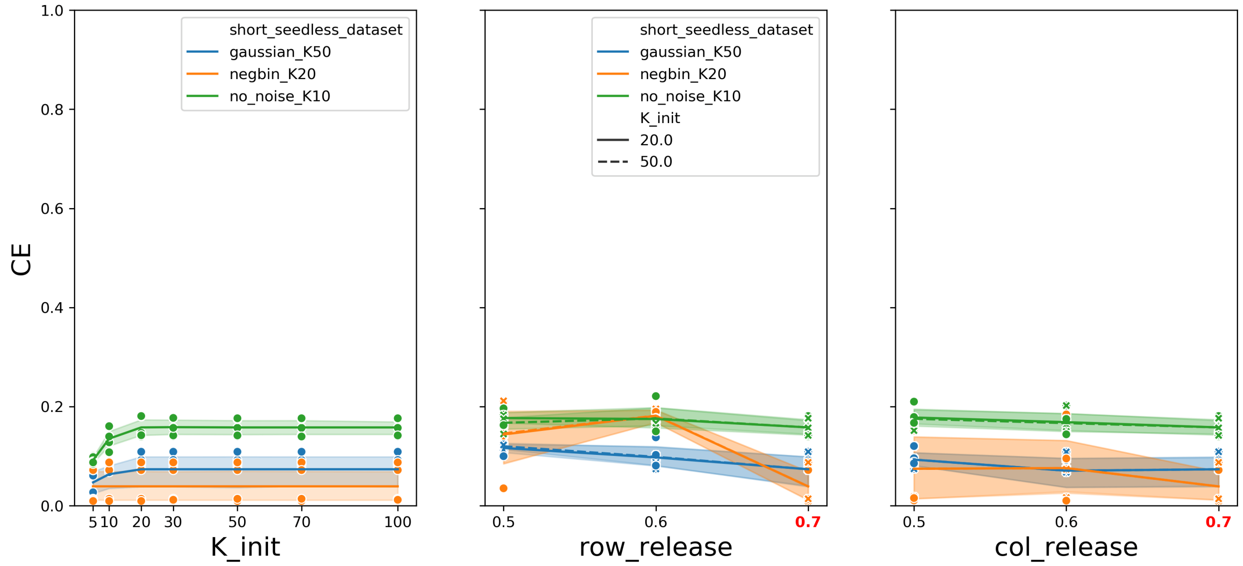

Figure S5: Parameter sweep for Plaid. Each plot shows the effect on the clustering error measure (CE) when a single parameter is varied, while the other parameters are kept at their default values. The default value for a parameter is highlighted in red on the x-axis. Higher clustering error values are better.

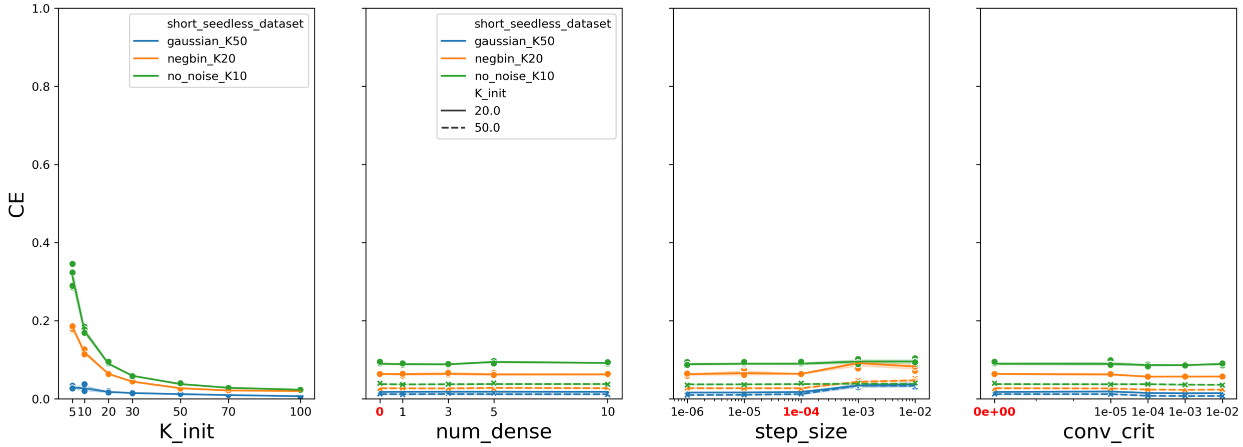

Figure S6: Parameter sweep for SDA. Each plot shows the effect on the clustering error measure (CE) when a single parameter is varied, while the other parameters are kept at their default values. The default value for a parameter is highlighted in red on the x-axis. Higher clustering error values are better.

S2.5.2 Convergence

SDA is one of the slower algorithms. We suspected this was partly due to its strict convergence criteria. The parameter *conv_crit* determines the largest allowed change in free energy at convergence, thus larger values make it easier for the algorithm to converge. The default value is 0. In Figure S6 we see there is no difference when *conv_crit* is 1×10^{-5}, but marginal differences for larger values. Using 1×10^{-5} seems to have little effect on performance, but decreases the median running time by 50% (Figure S7). We recommend users consider changing from the default value of *conv_crit* in this way to save computational time. We chose not to make the change in this study as it is valuable for us to show performance using the default values, since this is what most users will do.

S2.6 nsNMF

The only crucial parameter for nsNMF is θ which controls sparsity. The higher θ, the sparser the model. Surprisingly, we found that changing θ had little impact (Figure S8). The dataset that suffered worst performance from using a high value of θ (thus a sparser model) was the *gaussian_K50* dataset which had the smallest biclusters.

S2.7 SNMF

As with nsNMF, the important parameter is β, which controls sparsity. Larger β makes the model sparser, but we found that changing the sparsity had little impact on the performance (Figure S9).

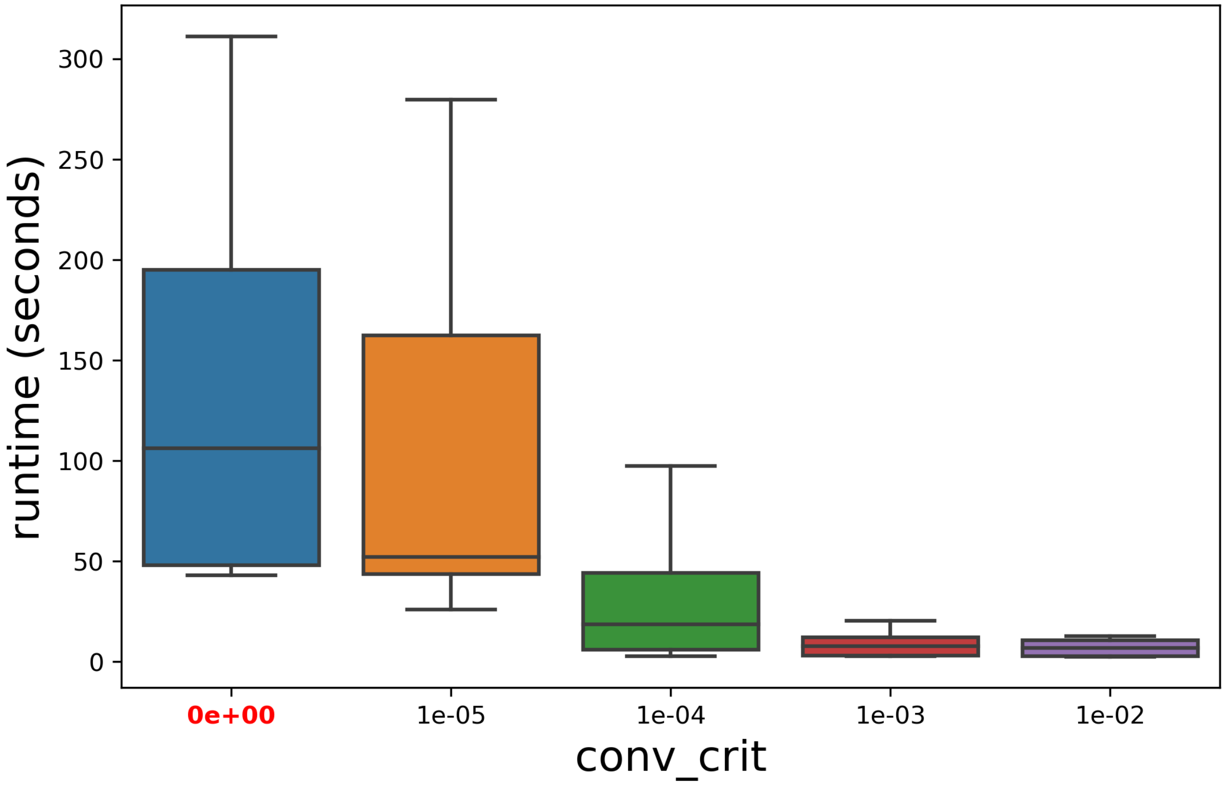

Figure S7: Running time of SDA with different *conv_crit* values.

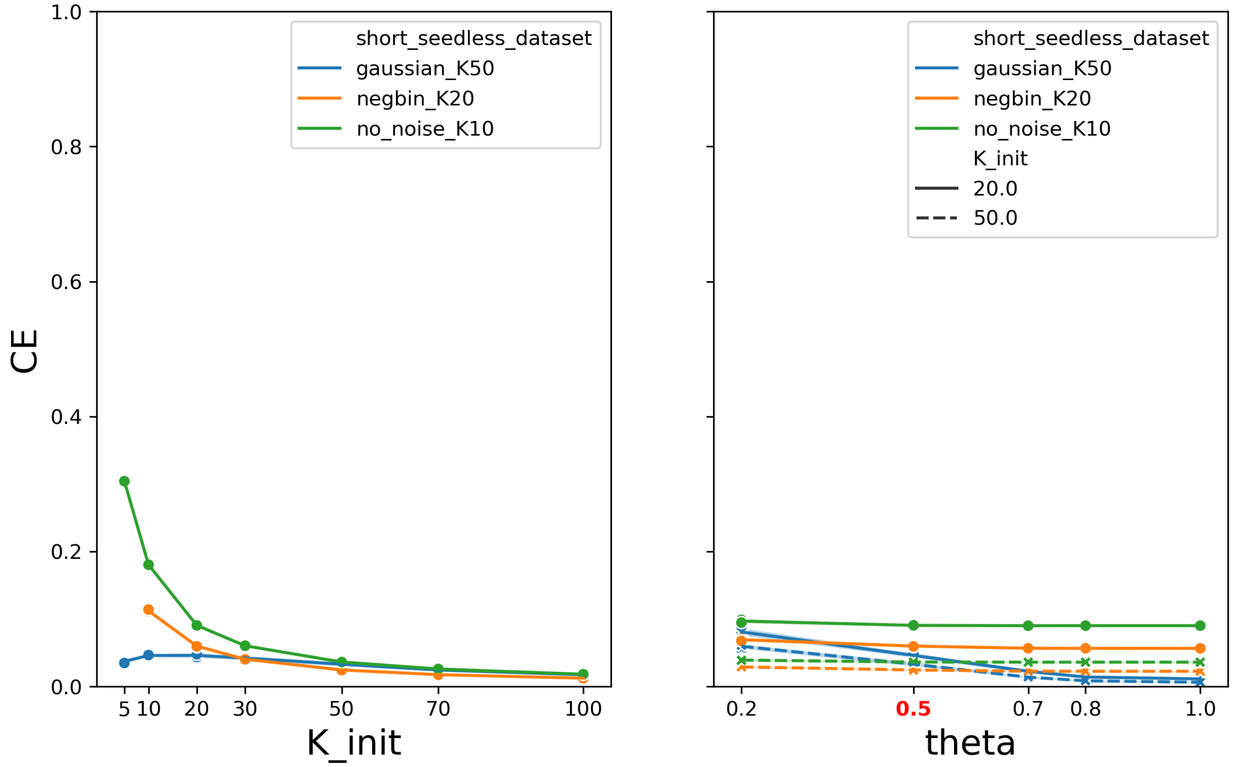

Figure S8: Parameter sweep for nsNMF. Each plot shows the effect on the clustering error measure (CE) when a single parameter is varied, while the other parameters are kept at their default values. The default value for a parameter is highlighted in red on the x-axis. Higher clustering error values are better.

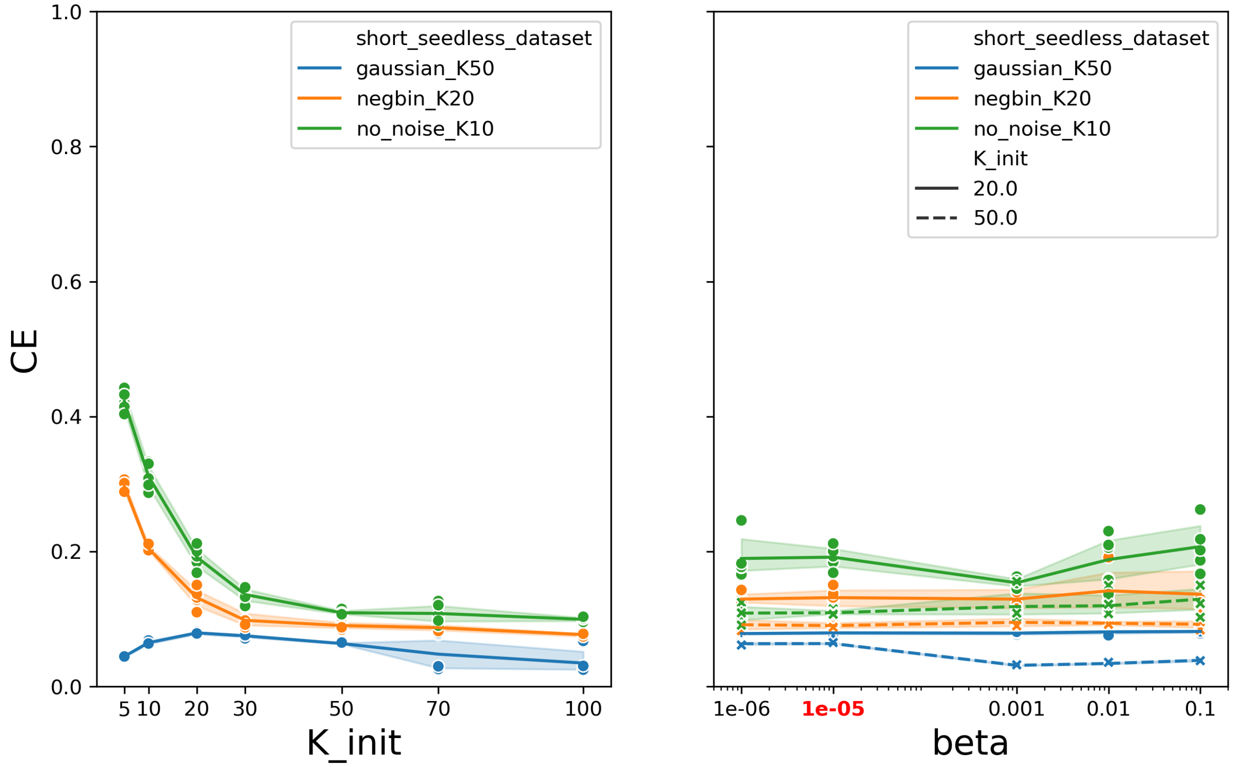

Figure S9: Parameter sweep for SNMF. Each plot shows the effect on the clustering error measure (CE) when a single parameter is varied, while the other parameters are kept at their default values. The default value for a parameter is highlighted in red on the x-axis. Higher clustering error values are better.

S2.8 SSLB

The parameter likely to have the biggest effect is the choice of prior for the values in B and X.

S2.8.1 Prior for B

The prior on B is a Beta-Binomial, with parameters a, b. Changing these seems to have little effect on the performance of SSLB (Figure S10).

S2.8.2 Prior for X

There are three different priors that can be used for X. The prior choice is controlled by the parameters *IBP* and d. If IBP $= 0$ then the simple Beta-Binomial prior is used; if IBP $= 1$ and $d = 0$ then a normal IBP is used; if IBP $= 1$ and $d \neq 0$ then a Pitman-Yor extension of the IBP is used.

The simplest is the same Beta-Binomial used for B, which has parameters $\tilde{a}, \tilde{b}$. We chose to experiment with using this Beta-Binomial prior, but, since it is not the default and generally performed worse than the other priors in the original paper [5], we decided not to investigate optimal parameters for this prior. Figure S10 shows that using the Beta-Binomial prior (i.e. IBP $= 0$) slightly degrades the perforance.

The prior used by SSLB by default is the IBP prior, which uses the parameter α. This has default value $\alpha = \frac{1}{N}$, i.e. it is dependent on the number of samples. All the datasets shown in Figure S10 have the same number of samples: $N \times T = 10 \times 10 = 100$, so the default value was 1/100 for all datasets.

Finally, the Pitman-Yor extension to the IBP prior uses both α and d. We only investigated the effect of changing d. $d = 0$ gives the standard IBP prior, and we did not find a significant difference when the Pitman-Yor extension was used, for any values of d (Figure S10).

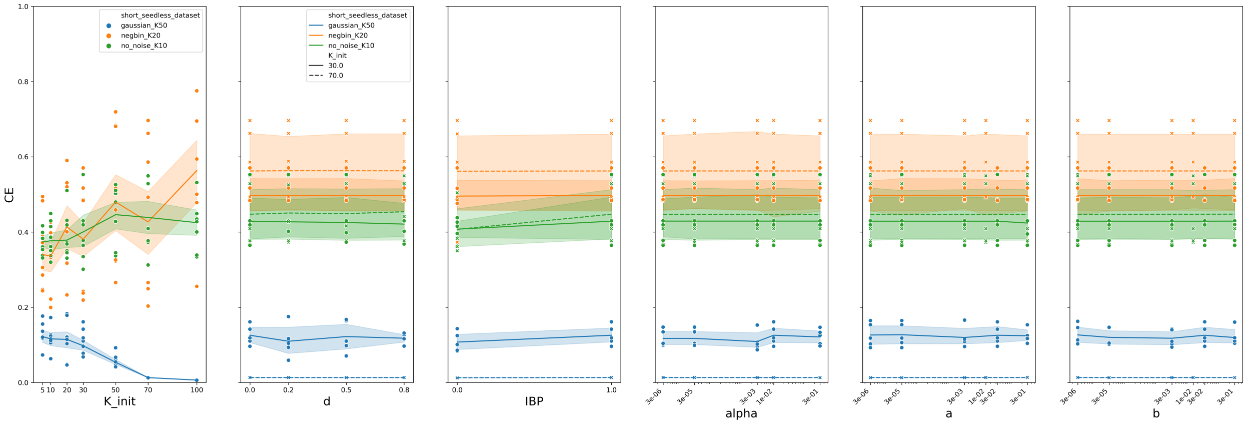

Figure S10: Parameter sweep for SSLB. Each plot shows the effect on the clustering error measure (CE) when a single parameter is varied, while the other parameters are kept at their default values. Default values were: $d = 0, \text{IBP} = 1$ (i.e. using the standard IBP prior), $\alpha = \frac{1}{N} = \frac{1}{100}$, $a = b = \frac{1}{K}$. Higher clustering error values are better.

S3 Evaluation Metrics

S3.1 Accuracy on simulated datasets

Common choices for metric to compare biclusterings, and thus to evaluate biclustering algorithms on simulated datasets, are the *consensus score* [1, 5] and the *recovery* and *relevance* scores [14, 5, 2]. Horta and Campello defined eight desirable properties of similarity measures for comparing biclusterings, including for example whether the measure penalises biclusterings with duplicated biclusters [18]. The consensus score satisfies only four of these desirable properties, and the recovery and relevance scores satisfy only one.

We chose to use the *clustering error* (CE) metric [19, 18] to evaluate biclustering accuracy, which was shown to satisfy all but one of the desirable properties defined by Horta and Campello, which is a great improvement on the consensus score and recovery and relevance scores commonly used to evaluate biclustering similarity.

S3.1.1 Metrics comparing two biclusterings

To be able to define metrics comparing two biclusterings, we first write biclusters as sets of elements. Suppose we have two factorisations $Y_{ij} = \sum_k X_{ik} B_{jk}$ and $\hat{Y}_{ij} = \sum_l \hat{X}_{il} \hat{B}_{jl}$. We write each bicluster as a set of the matrix elements that it contains: $A_k = \{(i, j) : X_{ik} \neq 0, B_{jk} \neq 0\}$ and $\hat{A}_l = \left\{(i, j) : \hat{X}_{il} \neq 0, \hat{B}_{jl} \neq 0\right\}$.

The Jaccard index, originally called the coefficient of community, [20] measures how closely two sets match, comparing their intersection to their union. Using the description of biclusters as sets, we can use the Jaccard index to measure how closely two biclusters match:

$$J(A_k, B_l) = \frac{\left|A_k \cap \hat{B}_l\right|}{\left|A_k \cap \hat{B}_l\right|} \tag{17}$$

The recovery and relevance scores [14] are defined as:

$$\begin{aligned} \text{Relevance} &:= \frac{1}{K} \sum_{k=1}^{K} \max_{l} \; J\left(A_k, \hat{A}_l\right), \\ \text{Recovery} &:= \frac{1}{L} \sum_{l=1}^{L} \max_{k} \; J\left(A_k, \hat{A}_l\right) \end{aligned} \tag{18}$$

The consensus score also makes use of the Jaccard index, but first calculates the best way to pair biclusters from each set. We can write a pairing of biclusters as $\left\{\left(A_{k_i}, \hat{A}_{l_i}\right)\right\}_{i=1}^{\min\{K,L\}}$ where each element of this set gives a pair: a bicluster A_{k_i} from A and the bicluster $\hat{A}_{l_i}$ from $\hat{A}$ that it is paired with. Note that if one biclustering contains more biclusters than the other, there will be some unpaired biclusters. We seek the pairing which maximises the following sum, the sum of the Jaccard indices of pairs divided by the number of biclusters

in the larger biclustering:

$$\text{Consensus} := \frac{1}{\max K, L} \sum_{i=1}^{\min K,L} \left| A_{k_i} \cap \hat{A}_{l_i} \right| \tag{19}$$

In practice the optimal pairing is found using the Hungarian algorithm for the assignment problem [21].

S3.1.2 Clustering error

The clustering error (CE) is defined as:

$$\text{CE}(A, \hat{A}) := \frac{d_{\max}}{|U|} \tag{20}$$

where d_{max} measures how much the biclusterings intersect and $|U|$ measures the total space collectively covered by the biclusterings, taking overlaps into account. Despite its name, CE is a measure of similarity between biclusters rather than dissimilarity.

We often use this measure to compare a biclustering $\hat{A} = \left\{\hat{A}_1, \ldots \hat{A}_L\right\}$ recovered by a algorithm to a biclustering $A = \{A_1, \ldots, A_K\}$ which we treat as the *true* biclustering. However, this measure is symmetric and can also be used when there is no 'truth', such as to compare two biclusterings returned by different runs of the same algorithm.

In a similar way to the consensus score, we use the Hungarian algorithm to find a pairing of biclusters from the two sets to maximise a sum, this time the sum of the intersections of pairs:

$$d := \sum_{i=1}^{\min K,L} \left| A_{k_i} \cap \hat{A}_{l_i} \right| \tag{21}$$

The maximum value this sum attains is called $d_{\max}$ and measures how much the biclusterings intersect.

The *size counting overlapping* of a union of two sets, called $|U|$, is

$$|U| := \sum_{i,j} \max\left\{\mathrm{N}_{i,j}, \hat{\mathrm{N}}_{i,j}\right\} \tag{22}$$

where $N_{i,j} := |\{k : (i,j) \in A_k\}|$ gives the number of times that the matrix element (i,j) is included in biclusters in A and $\hat{N}_{i,j} := \left|\left\{l : (i,j) \in \hat{A}_l\right\}\right|$ is the same for $\hat{A}$.

S4 Simulated datasets

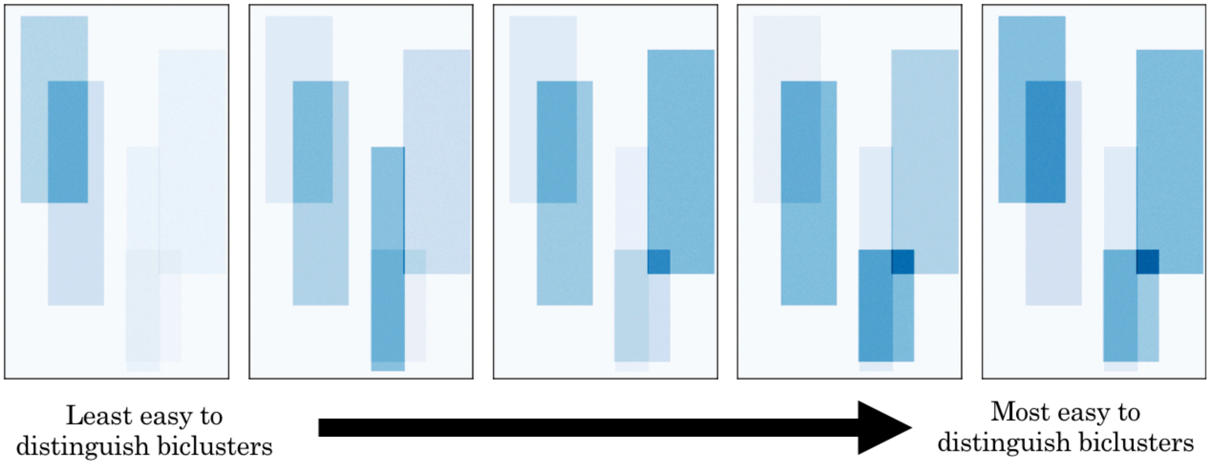

Figure S11: Illustration of simulated datasets. Bicluster shapes were simulated for a dataset with 600 samples, 1000 genes and five biclusters, with just one tissue. Means for each bicluster and the bicluster values were simulated five times, with each shown in a separate grid. They have been ordered by how easy it is to distinguish the biclusters.

Table S3: Summary of simulated datasets. The attributes of the datasets are displayed in bold if they differ from the base dataset. N is the number of samples, T the number of tissues and G the number of genes in the dataset.

Name	N	T	G	Bicluster sizes	K	Noise type	Noise level	Distribution
base	10	10	1000	mixed	20	Negative binomial	Low	NB$(n_k, 0.3)$
N50-T2	**50**	**2**	1000	mixed	20	Negative binomial	Low	NB$(n_k, 0.3)$
N10-T20	10	**20**	1000	mixed	20	Negative binomial	Low	NB$(n_k, 0.3)$
N100-T10	**100**	10	1000	mixed	20	Negative binomial	Low	NB$(n_k, 0.3)$
N500-T10	**500**	10	1000	mixed	20	Negative binomial	Low	NB$(n_k, 0.3)$
G100	10	10	**100**	mixed	20	Negative binomial	Low	NB$(n_k, 0.3)$
G5000	10	10	**5000**	mixed	20	Negative binomial	Low	NB$(n_k, 0.3)$
large-K20	**300**	**20**	**10000**	mixed	20	Negative binomial	Low	NB$(n_k, 0.3)$
negbin-medium	10	10	1000	mixed	20	Negative binomial	**Medium**	NB$(n_k, \mathbf{0.1})$
negbin-high	10	10	1000	mixed	20	Negative binomial	**High**	NB$(n_k, \mathbf{0.01})$
Gaussian	10	10	1000	mixed	20	**Gaussian**	Low	$\boldsymbol{\mathcal{N}(\mu_k, 20^2)}$
Gaussian-medium	10	10	1000	mixed	20	**Gaussian**	**Medium**	$\boldsymbol{\mathcal{N}(\mu_k, 100^2)}$
Gaussian-high	10	10	1000	mixed	20	**Gaussian**	**High**	$\boldsymbol{\mathcal{N}(\mu_k, 300^2)}$
noiseless	10	10	1000	mixed	20	**No noise**	-	-
sparse	10	10	1000	**sparse**	20	Negative binomial	Low	NB$(n_k, 0.3)$
dense	10	10	1000	**dense**	20	Negative binomial	Low	NB$(n_k, 0.3)$
sparse-square	10	10	1000	**sparse, square**	20	Negative binomial	Low	NB$(n_k, 0.3)$
dense-square	10	10	1000	**dense, square**	20	Negative binomial	Low	NB$(n_k, 0.3)$
K5	10	10	1000	mixed	**5**	Negative binomial	Low	NB$(n_k, 0.3)$
K10	10	10	1000	mixed	**10**	Negative binomial	Low	NB$(n_k, 0.3)$
K50	10	10	1000	mixed	**50**	Negative binomial	Low	NB$(n_k, 0.3)$
K70	10	10	1000	mixed	**70**	Negative binomial	Low	NB$(n_k, 0.3)$
large-K100	**300**	**20**	**10000**	mixed	**100**	Negative binomial	Low	NB$(n_k, 0.3)$
large-K400	**300**	**20**	**10000**	mixed	**400**	Negative binomial	Low	NB$(n_k, 0.3)$

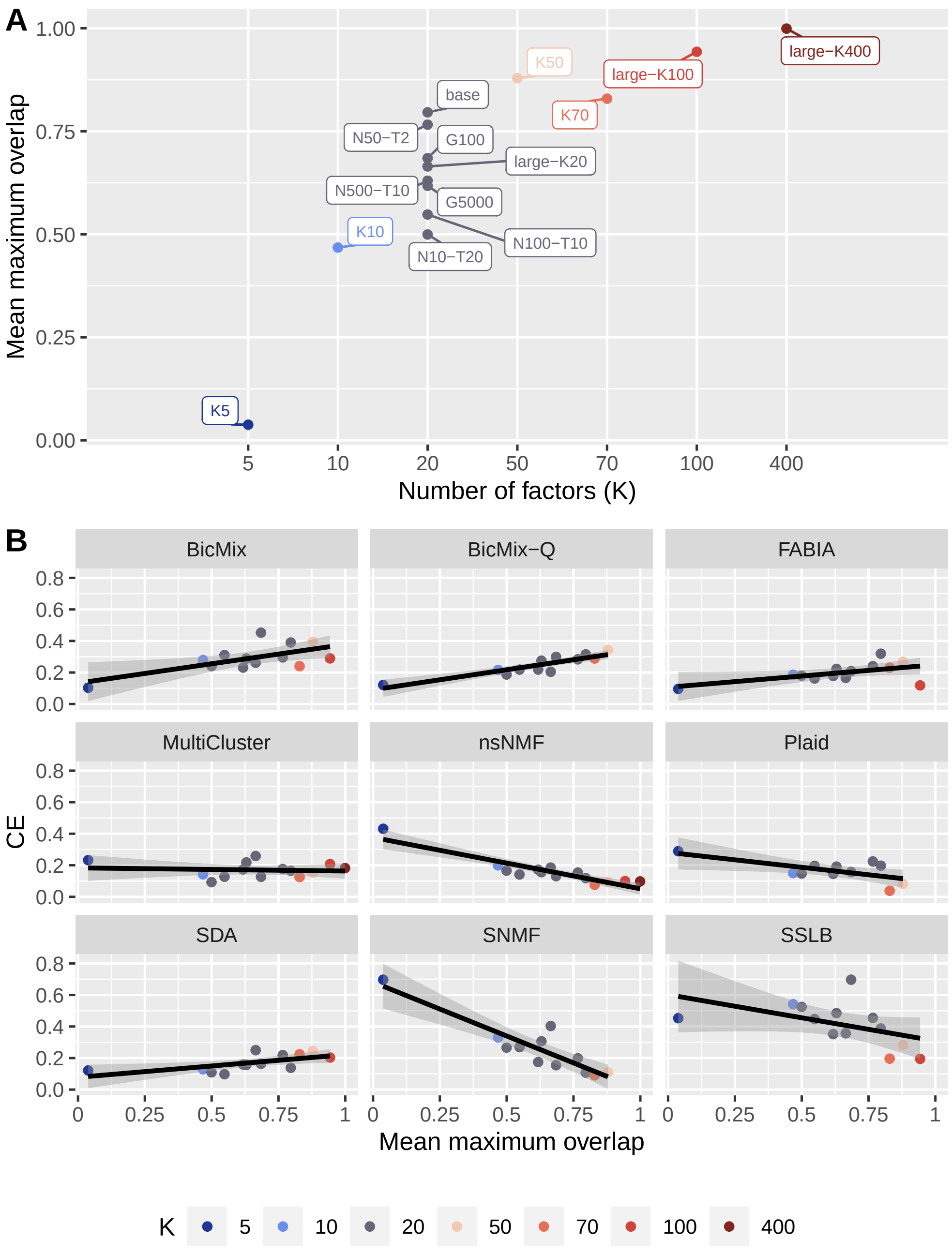

Figure S12: Overlap between biclusters in simulated datasets and the effect this overlap has on performance. In each plot there is a point for each dataset type, coloured by the number of biclusters contained in the dataset. Mean maximum overlap is a measure of overlap between biclusters in a dataset. For each bicluster in a dataset we identify the bicluster with which it shares the greatest percentage of its cells. We then take the mean of this maximum overlap percentage across all biclusters in the dataset to give a measure of the average overlap in the dataset. (A) Mean maximum overlap plotted against the number of biclusters. The properties of the datasets are given in Table S3. (B) Mean maximum overlap plotted against CE for each algorithm. Higher values of CE are better.

S5 Benaroya dataset

Table S4: Number of samples in Benaroya RNA-seq dataset for each cell type and disease. ALS stands for amyotrophic lateral sclerosis, MS stands for multiple sclerosis. Note that two sets of samples were taken from patients with MS: one set before treatment with IFN-beta and one set after treatment. The dataset is available on ArrayExpress under accession number E-GEOD-60424, collected by the Benaroya Research Institute [22]. The full dataset includes 14 samples from NK cells, but we chose to use only the cell types which were collected for every patient and thus excluded the NK cell samples.

	**Lymphoid**			**Myeloid**		
	B-Cells	CD4	CD8	Monocytes	Neutrophils	Whole blood
ALS	3	3	3	3	3	3
Type 1 Diabetes	4	4	4	4	4	4
MS (after treatment)	3	3	3	3	3	3
MS (before treatment)	3	3	3	3	3	3
Sepsis	3	3	3	3	3	3
Healthy control	4	4	4	4	4	4
**Total**	**20**	**20**	**20**	**20**	**20**	**20**

S6 Post-processing

FABIA, SDA, SNMF and nsNMF return many biclusters containing all genes and all samples, as shown in Figure S13 and Figure S14. Without post-processing, these algorithms would thus receive very poor scores. We hypothesised that many values in the matrices defining these biclusters were close to 0, and by setting these small values to 0 we would reveal the underlying structure of the biclusters and thus enable meaningful evaluation.

We explored the effect of setting elements of X and B to zero if they were below a certain threshold, with various choices of threshold α. Some algorithms, most notably BicMix, return sample loadings X_{ik} and gene loadings B_{jk} on very different scales. To avoid having to account for this imbalance for each algorithm individually, we scaled the vectors x_k and b_k for each bicluster to have L_2 norm equal to 1 before removing any elements. Thus, when using a threshold α, we set any elements satisfying the following inequality to be 0:

$$\frac{X_{ik}}{\sqrt{\sum_l |X_{lk}|^2}} < \alpha \tag{23}$$

and similarly for any elements of B satisfying:

$$\frac{B_{jk}}{\sqrt{\sum_l |B_{lk}|^2}} < \alpha \tag{24}$$

We found that a threshold of 0.01 was the best choice for all algorithms for simulated datasets (Figure S15), and was also sensible for real datasets (Figure S18 and Figure S16).

It is worth noting that, since thresholding appears to affect different metrics in similar ways, it would have been possible to choose a sensible threshold using only metrics that we would have from a standard real dataset, such as redundancy of recovered biclusters and reconstruction error. Also, although we believe the threshold chosen is suitable for all algorithms, there is potential for further fine-tuning for individual algorithms.

After thresholding, the distribution of bicluster sizes is less extreme. Figure S20 and S19 show the distribution of bicluster sizes in terms of samples and genes respectively. Interestingly some algorithms such as BicMix-Q and FABIA have a similar distribution regardless of dataset type, whereas others such as BicMix and SNMF seem to adapt to the distribution of bicluster sizes.

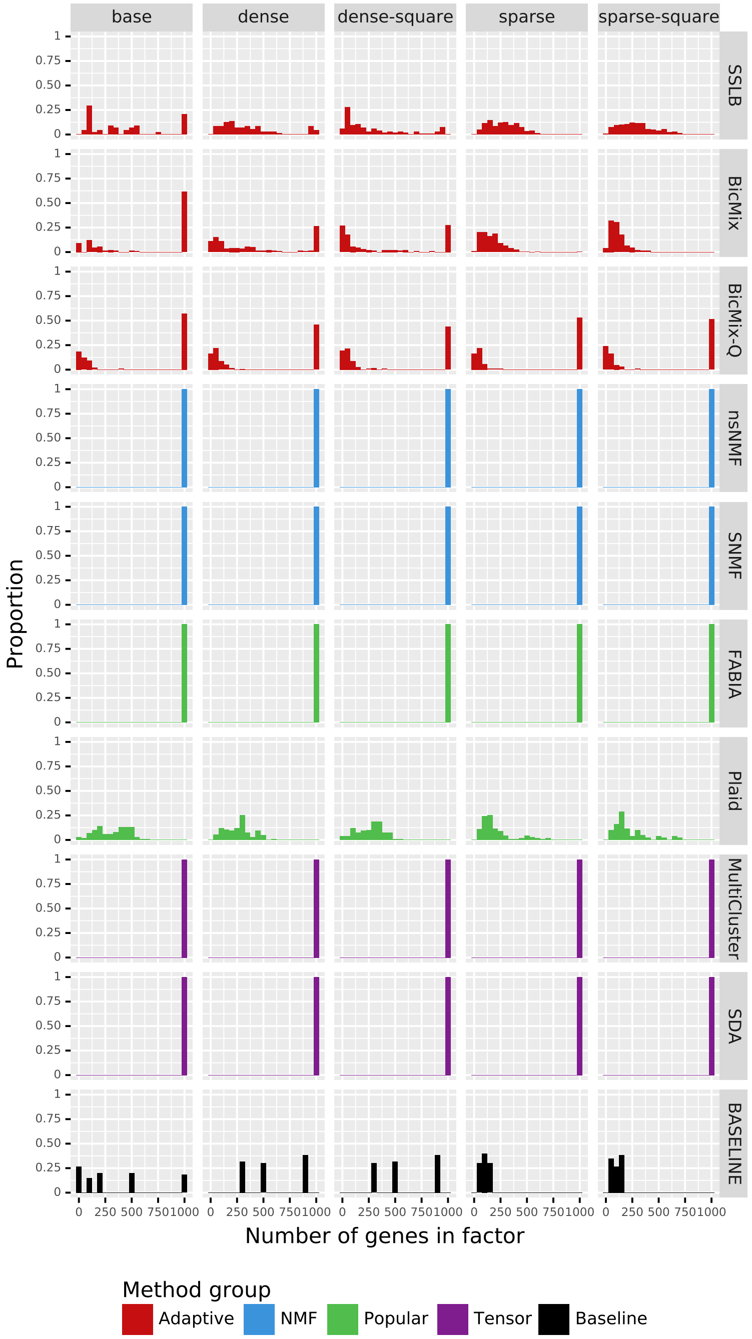

Figure S13: Number of genes contained in biclusters, with no thresholding applied. Each column shows a dataset with different distribution of bicluster sizes. Each graph in the grid shows the relative frequency of the number of genes contained in biclusters. The last row (BASELINE) shows the distribution of number of genes in biclusters in the datasets themselves, and the other rows show the distribution for each algorithm in turn. Note that 100% of the biclusters recovered by FABIA, MultiCluster, SDA, SNMF and nsNMF contain all 1000 genes. BicMix, BicMix-Q and SSLB used $K_{init} = 40$ and the remaining algorithms used $K_{init} = 20$.

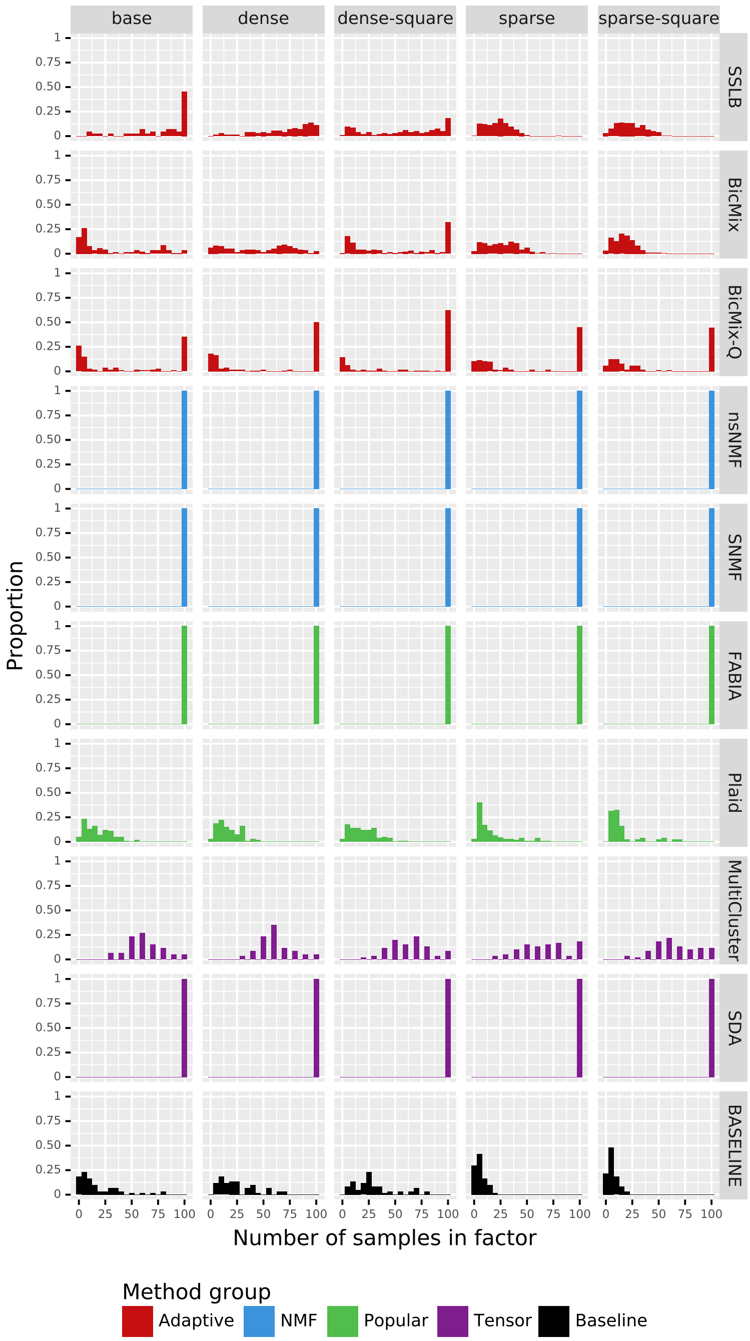

Figure S14: Number of samples contained in biclusters, with no thresholding applied. Each column shows a dataset with different distribution of bicluster sizes. Each graph in the grid shows the relative frequency of the number of samples contained in biclusters. The last row (BASELINE) shows the distribution of number of samples in biclusters in the datasets themselves, and the other rows show the distribution for each algorithm in turn. Note that 100% of the biclusters recovered by FABIA, SDA, SNMF and nsNMF contain all 100 samples. BicMix, BicMix-Q and SSLB used $K_{init} = 40$ and the remaining algorithms used $K_{init} = 20$.

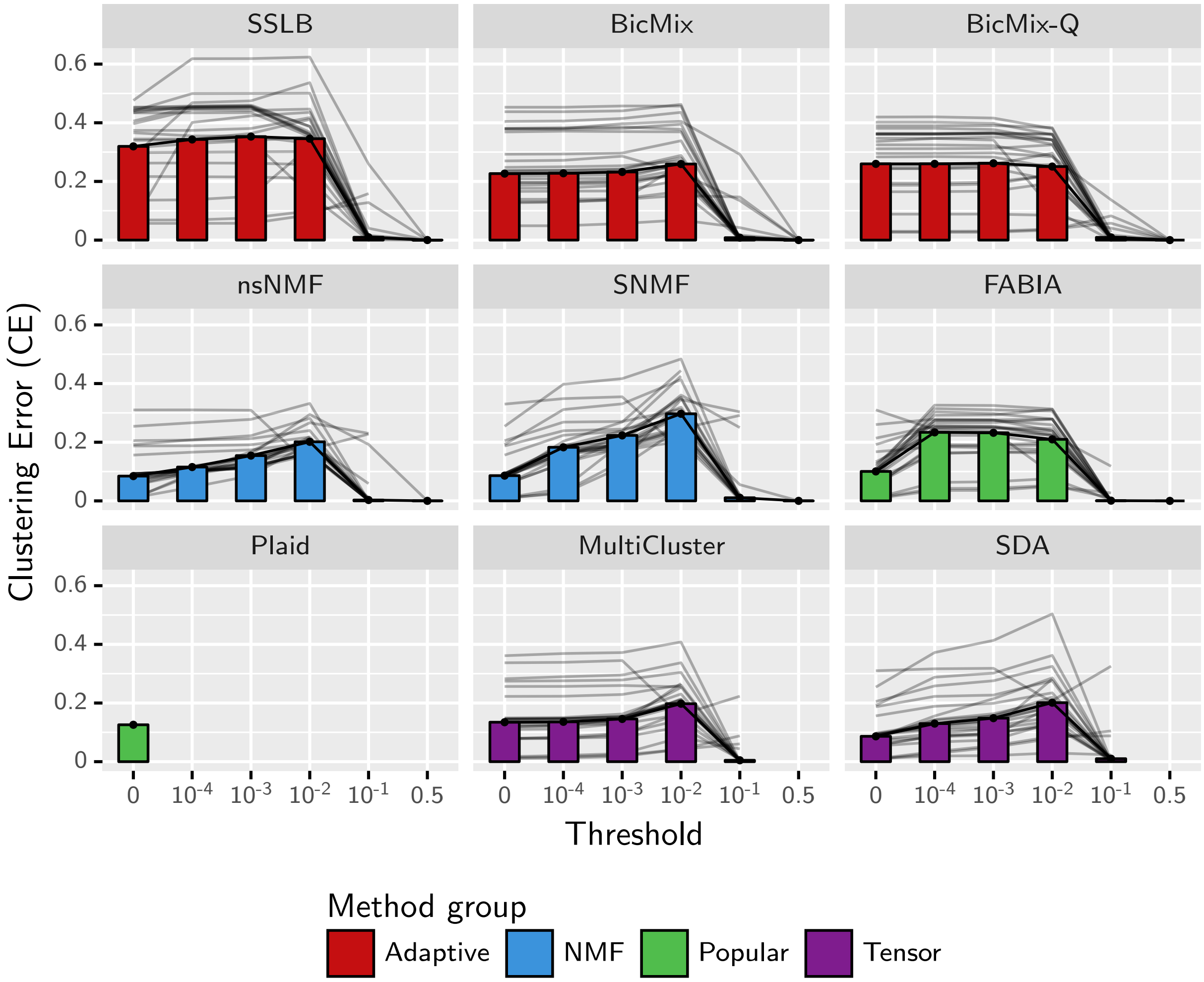

Figure S15: Biclustering accuracy (CE) against the threshold for inclusion in bicluster. Higher values of CE are better. The thresholding process is described in Supplementary Section S6. The median CE across all simulated datasets and all values of K_{init} is shown by the bars. The grey lines show the median for each dataset type. FABIA, MultiCluster, SDA, SNMF and nsNMF show significant improvement in CE as the thresholding becomes stricter and more genes and samples are moved from the recovered biclusters. BicMix and SSLB are relatively unaffected by the thresholding process, until the threshold reaches 0.1. Thresholding could not be applied to Plaid.

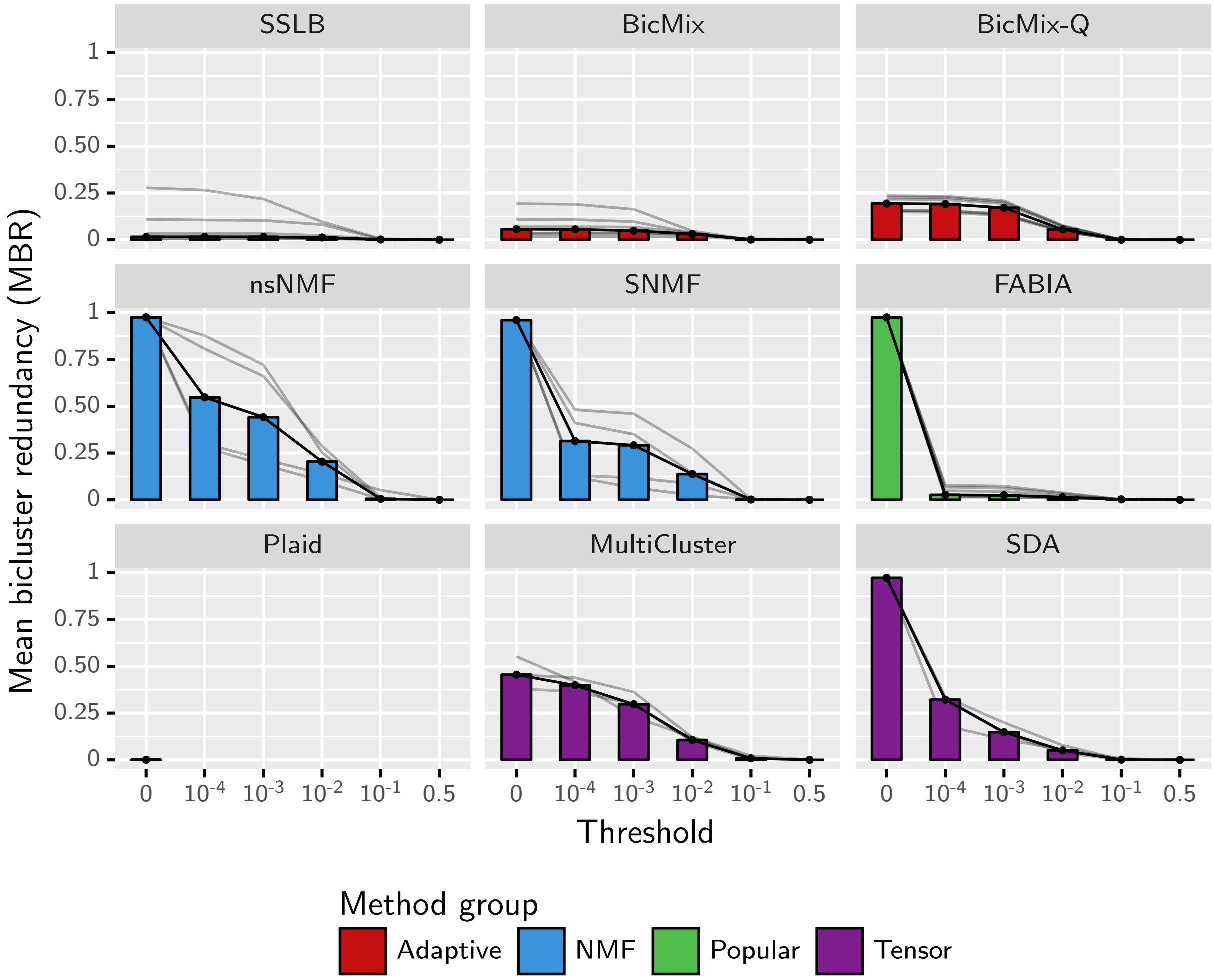

Figure S16: Average similarity of biclusters (MBR) within a run plotted against the threshold for inclusion in bicluster, for IMPC datasets. MBR is in the range $[0, 1]$, and a lower value is preferred as a high value suggests that the algorithm is returning many copies of the same bicluster. The thresholding process is described in Section S6. The median of the MBR measure across all IMPC datasets and all values of K_{init} is shown by the bars. The grey lines show the median for each dataset type. Note that without thresholding (threshold 0) the biclusters within each run by FABIA, SDA and nsNMF are almost all identical.

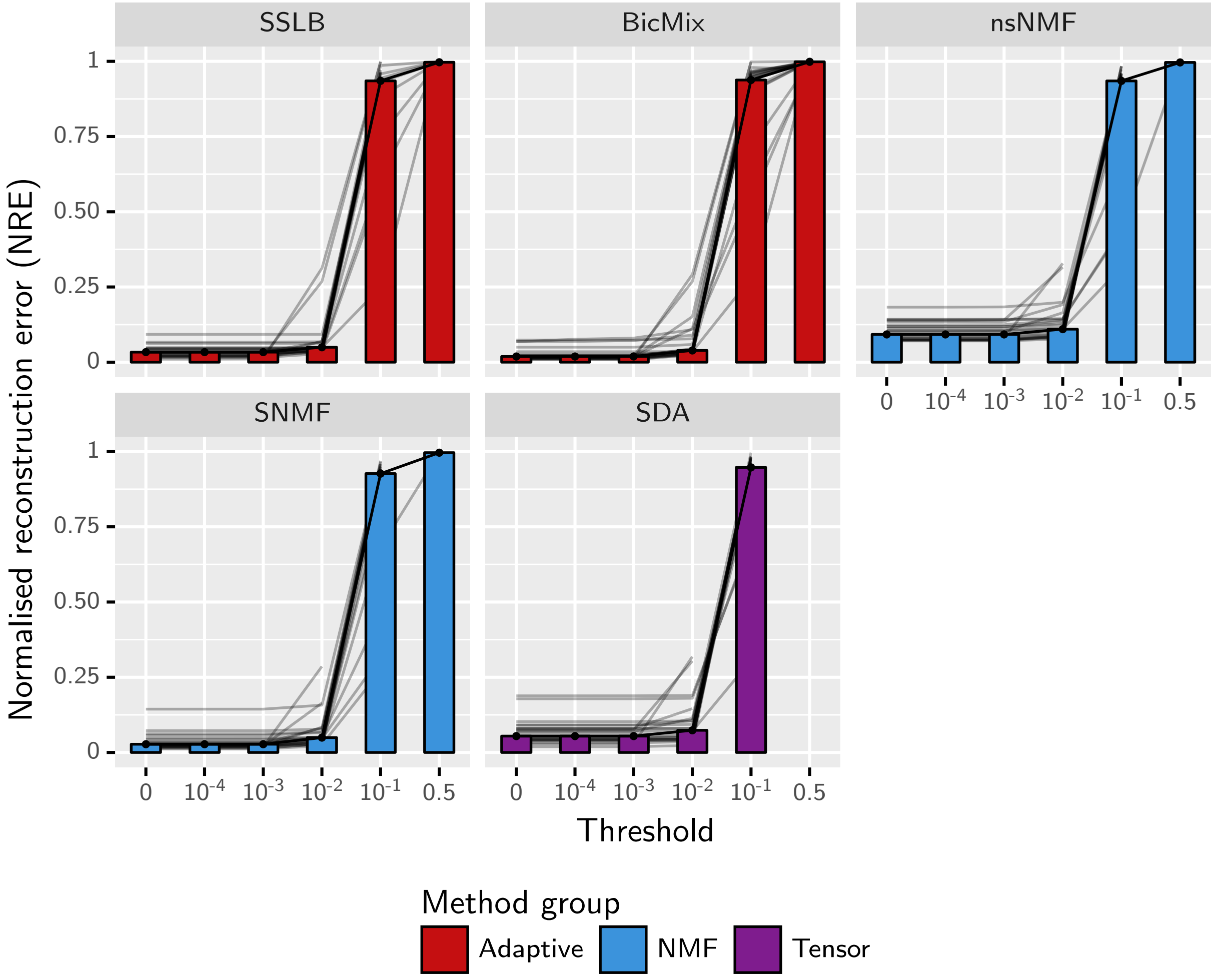

Figure S17: Normalised reconstruction error on simulated datasets, plotted against the threshold for inclusion in bicluster. The thresholding process is described in Section S6. The median of this measure across all simulated datasets and all values of K_{init} is shown by the bars. The grey lines show the median for each dataset type. Note that without thresholding (threshold 0) the biclusters within each run by FABIA, SDA and nsNMF are almost all identical.

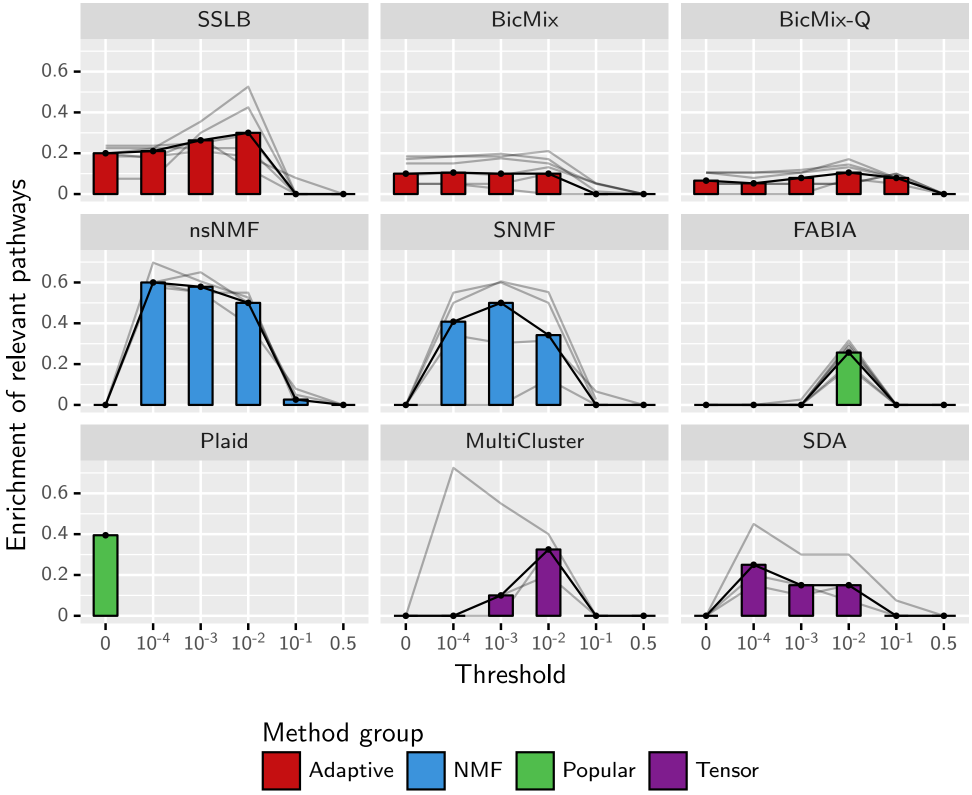

Figure S18: Biclustering performance on IMPC datasets, measured by the mean proportion of knocked-out genes for which the biclusters best matching the samples where the gene was knocked out is enriched for at least one pathway containing the knocked-out gene. A higher score is preferred. Enrichment is measured using the one-tailed hypergeometric test adjusted for multiple testing using the Benjamini-Yekutieli correction, using a threshold for significance of 0.05. The thresholding process is described in Supplementary Section S6. The median of this measure across all IMPC datasets and all values of K_{init} is shown by the bars. The grey lines show the median for each dataset type. As with simulated datasets, the performance of FABIA, MultiCluster, SDA, SNMF and nsNMF show significant improvement as the thresholding becomes stricter.

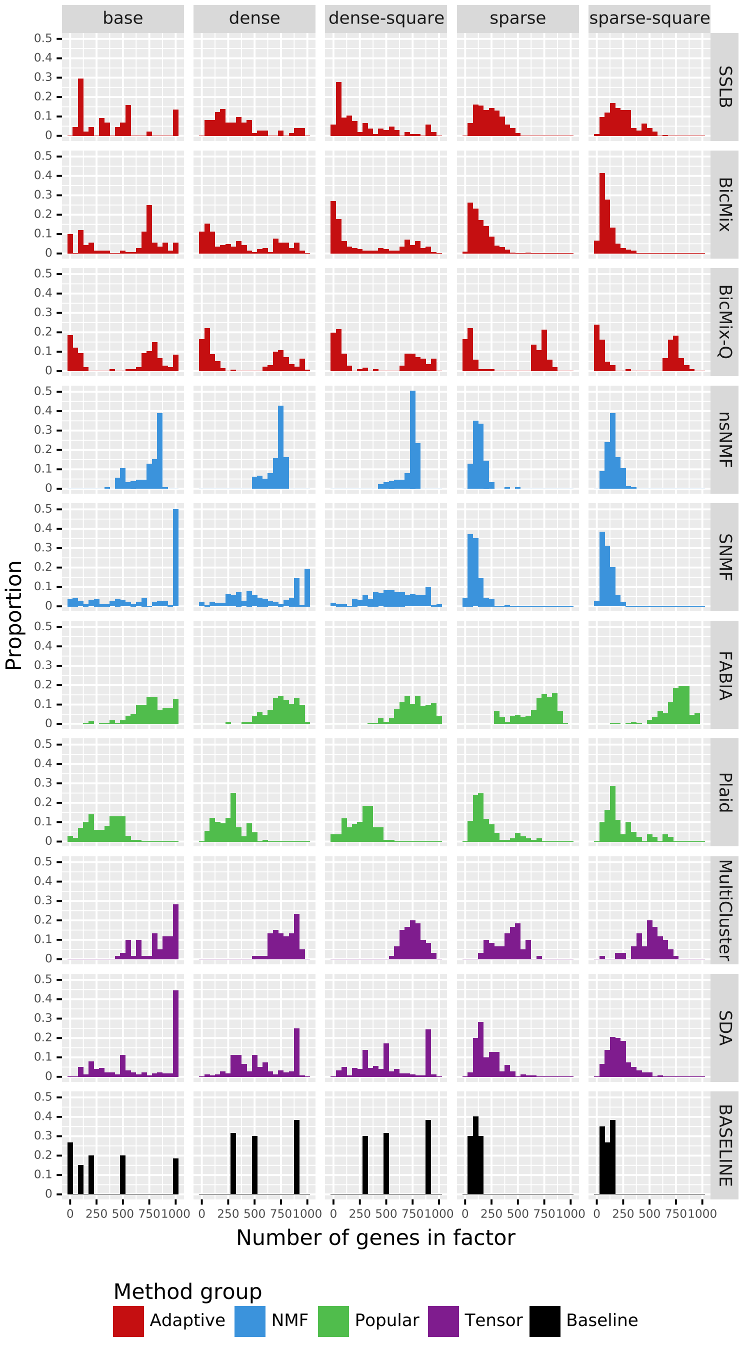

Figure S19: Number of genes contained in biclusters, with thresholding applied as specified in Section S6. Each column shows a dataset with different distribution of bicluster sizes. Each graph in the grid shows the relative frequency of the number of genes contained in biclusters. The last row shows the distribution of number of genes in biclusters in the datasets themselves, and the other rows show the distribution for each algorithm in turn. BicMix, BicMix-Q and SSLB used $K_{init} = 40$ and the remaining algorithms used $K_{init} = 20$.

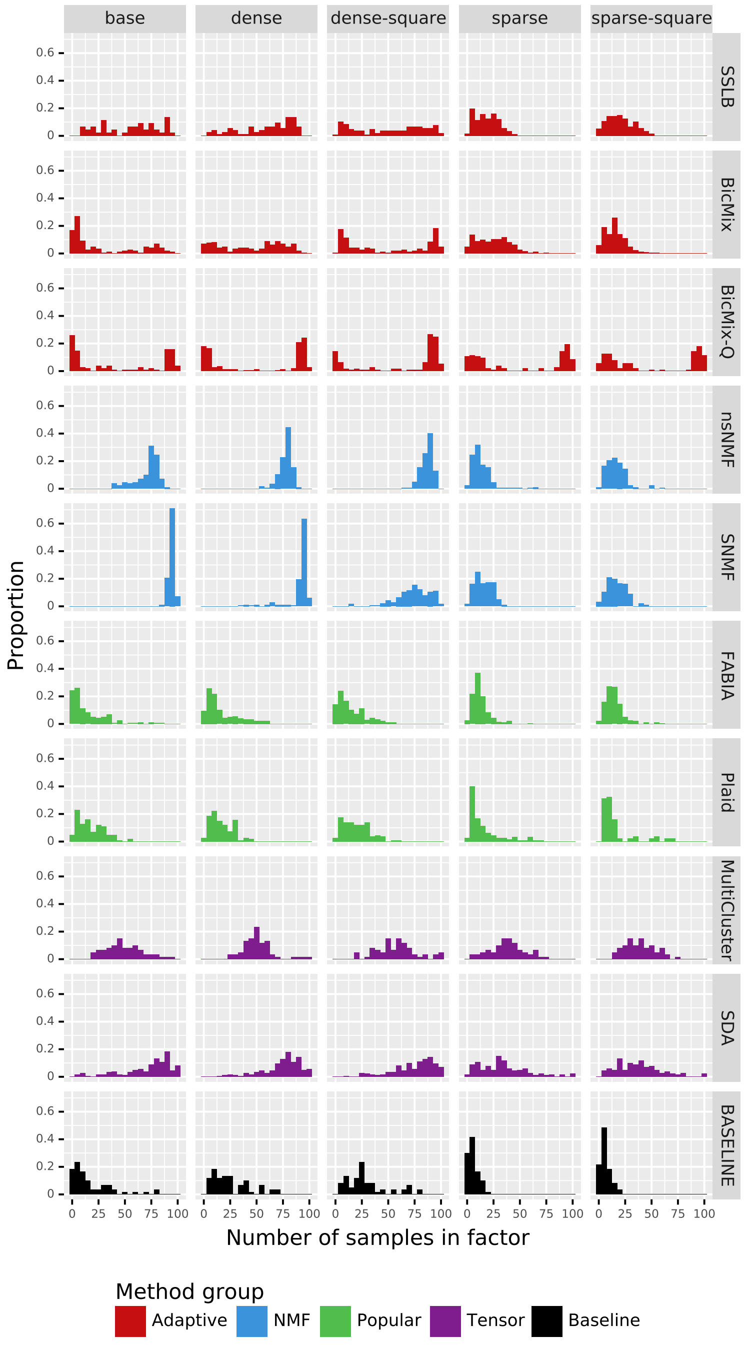

Figure S20: Number of samples contained in biclusters, with thresholding applied as specified in Section S6. Each column shows a dataset with different distribution of bicluster sizes. Each graph in the grid shows the relative frequency of the number of samples contained in biclusters. The last row shows the distribution of number of samples in biclusters in the datasets themselves, and the other rows show the distribution for each algorithm in turn. BicMix, BicMix-Q and SSLB used $K_{init} = 40$ and the remaining algorithms used $K_{init} = 20$.

S7 Extra results

Table S5: Number of failed runs by each algorithm on each simulated dataset, restricted to runs with the value of K_{init} chosen for analysis. $K_{init} = K$ for all algorithms except the *Adaptive* algorithms. Since they are designed to be run with an overestimate of the number of biclusters, *Adaptive* algorithms use $K_{init} = 25$, except for the datasets with varying K (the last section of the table), for which they use $K_{init} = K + 10$. Failures are counted as runs that crashed, exceeded the time limit of 12 hours, exceeded the memory limit of 6GB or that failed to find any biclusters. Each cell displays the number of failures as a fraction of total number of attempted runs: *Failures/Attempts*. When all attempts failed, the counts are shown in bold.

	Adaptive			***NMF***		***Popular***		***Tensor***	
**Dataset**	SSLB	BicMix	BicMix-Q	nsNMF	SNMF	FABIA	Plaid	MultiCluster	SDA
base	0 / 9	0 / 9	0 / 9	0 / 9	0 / 9	0 / 9	0 / 9	0 / 9	0 / 9
N50-T2	0 / 9	0 / 9	0 / 9	0 / 9	0 / 9	0 / 9	0 / 9	0 / 9	0 / 9
N10-T20	0 / 9	2 / 9	0 / 9	0 / 9	0 / 9	0 / 9	0 / 9	0 / 9	0 / 9
N100-T10	0 / 9	0 / 9	0 / 9	0 / 9	0 / 9	0 / 9	0 / 9	0 / 9	0 / 9
N500-T10	0 / 9	0 / 9	0 / 9	0 / 9	0 / 9	0 / 9	0 / 9	0 / 9	0 / 9
G100	0 / 9	0 / 9	0 / 9	0 / 9	0 / 9	0 / 9	0 / 9	0 / 9	0 / 9
G5000	0 / 9	3 / 9	1 / 9	0 / 9	0 / 9	0 / 9	0 / 9	0 / 9	0 / 9
large-K20	0 / 9	1 / 9	0 / 9	0 / 9	0 / 9	0 / 9	**9 / 9**	0 / 9	0 / 9
Negbin-medium	0 / 9	0 / 9	0 / 9	0 / 9	0 / 9	0 / 9	0 / 9	0 / 9	0 / 9
Negbin-high	0 / 9	0 / 9	0 / 9	0 / 9	0 / 9	0 / 9	0 / 9	0 / 9	0 / 9
Gaussian	0 / 9	3 / 9	0 / 9	0 / 9	0 / 9	0 / 9	0 / 9	0 / 9	0 / 9
Gaussian-medium	0 / 9	2 / 9	0 / 9	0 / 9	0 / 9	0 / 9	0 / 9	0 / 9	0 / 9
Gaussian-high	0 / 9	0 / 9	0 / 9	0 / 9	0 / 9	0 / 9	0 / 9	0 / 9	0 / 9
noiseless	0 / 9	0 / 9	0 / 9	0 / 9	0 / 9	0 / 9	0 / 9	0 / 9	0 / 9
sparse	0 / 9	0 / 9	0 / 9	0 / 9	0 / 9	0 / 9	0 / 9	0 / 9	0 / 9
dense	0 / 9	0 / 9	0 / 9	0 / 9	0 / 9	0 / 9	0 / 9	0 / 9	0 / 9
sparse-square	0 / 9	0 / 9	0 / 9	0 / 9	0 / 9	0 / 9	0 / 9	0 / 9	0 / 9
dense-square	0 / 9	1 / 9	0 / 9	0 / 9	0 / 9	0 / 9	0 / 9	0 / 9	0 / 9
K5	0 / 9	0 / 9	0 / 9	0 / 9	0 / 9	0 / 9	0 / 9	0 / 9	0 / 9
K10	0 / 9	0 / 9	0 / 9	0 / 9	0 / 9	0 / 9	0 / 9	0 / 9	0 / 9
K50	0 / 9	1 / 9	0 / 9	0 / 9	0 / 9	0 / 9	0 / 9	0 / 9	0 / 9
K70	0 / 9	0 / 9	0 / 9	0 / 9	0 / 9	0 / 9	0 / 9	0 / 9	0 / 9
large-K100	4 / 9	7 / 9	**9 / 9**	0 / 9	**9 / 9**	0 / 9	**9 / 9**	0 / 9	0 / 9
large-K400	**9 / 9**	**9 / 9**	**9 / 9**	0 / 9	**9 / 9**	**9 / 9**	**9 / 9**	7 / 9	**9 / 9**
**Total**	13 / 216	29 / 216	19 / 216	0 / 216	18 / 216	9 / 216	27 / 216	7 / 216	9 / 216

Table S6: Number of failed runs by each algorithm on each IMPC dataset, restricted to runs with $K_{init} = 50$. Failures are counted as runs that crashed, exceeded the time limit of 12 hours, exceeded the memory limit of 6GB or that failed to find any biclusters. Each cell displays the number of failures as a fraction of total number of attempted runs: *Failures/Attempts*. When all attempts failed, the counts are shown in bold.

	Adaptive			***NMF***		***Popular***		***Tensor***	
**Dataset**	SSLB	BicMix	BicMix-Q	nsNMF	SNMF	FABIA	Plaid	MultiCluster	SDA
Size factor	0 / 10	0 / 10	0 / 10	1 / 10	0 / 10	0 / 10	**10 / 10**	0 / 0	0 / 0
Log	0 / 10	1 / 10	0 / 10	0 / 10	0 / 10	0 / 10	0 / 10	0 / 0	0 / 0
Gaussian	0 / 10	0 / 10	0 / 10	0 / 0	0 / 0	0 / 10	5 / 10	0 / 0	0 / 0
Size factor (Tensor)	0 / 10	0 / 10	2 / 10	0 / 10	0 / 10	0 / 10	**10 / 10**	0 / 10	0 / 10
Log (Tensor)	0 / 10	0 / 10	1 / 10	0 / 10	0 / 10	0 / 10	0 / 10	0 / 10	0 / 10
Gaussian (Tensor)	0 / 10	0 / 10	0 / 10	0 / 0	0 / 0	0 / 10	2 / 10	0 / 10	0 / 10
**Total**	0 / 60	1 / 60	3 / 60	1 / 40	0 / 40	0 / 60	27 / 60	0 / 30	0 / 30

Table S7: Number of failed runs by each algorithm on each simulated dataset. Failures are counted as runs that crashed, exceeded the time limit of 12 hours, exceeded the memory limit of 6GB or that failed to find any biclusters. Each cell displays the number of failures as a fraction of total number of attempted runs: *Failures/Attempts*. When all attempts failed, the counts are shown in bold.

	Adaptive			***NMF***		***Popular***		***Tensor***	
**Dataset**	SSLB	BicMix	BicMix-Q	nsNMF	SNMF	FABIA	Plaid	MultiCluster	SDA
base	0 / 45	2 / 45	0 / 45	0 / 27	0 / 27	9 / 27	0 / 27	0 / 27	0 / 27
N50-T2	0 / 27	0 / 27	0 / 27	0 / 18	0 / 18	0 / 18	0 / 18	0 / 18	0 / 18
N10-T20	0 / 27	3 / 27	0 / 27	0 / 18	0 / 18	0 / 18	0 / 18	0 / 18	0 / 18
N100-T10	0 / 27	0 / 27	0 / 27	0 / 18	0 / 18	0 / 18	0 / 18	0 / 18	0 / 18
N500-T10	0 / 27	0 / 27	1 / 27	0 / 18	0 / 18	0 / 18	0 / 18	0 / 18	0 / 18
G100	0 / 27	0 / 27	0 / 27	0 / 18	0 / 18	0 / 18	0 / 18	0 / 18	0 / 18
G5000	0 / 27	6 / 27	2 / 27	0 / 18	0 / 18	0 / 18	0 / 18	0 / 18	0 / 18
large-K20	0 / 27	2 / 27	0 / 27	0 / 18	0 / 18	0 / 18	**18 / 18**	0 / 18	0 / 18
Negbin-medium	0 / 27	0 / 27	0 / 27	0 / 18	0 / 18	0 / 18	0 / 18	1 / 18	0 / 18
Negbin-high	0 / 27	0 / 27	0 / 27	0 / 18	0 / 18	0 / 18	0 / 18	0 / 18	0 / 18
Gaussian	0 / 27	3 / 27	0 / 27	0 / 18	0 / 18	0 / 18	0 / 18	0 / 18	0 / 18
Gaussian-medium	0 / 27	2 / 27	0 / 27	0 / 18	0 / 18	0 / 18	0 / 18	0 / 18	0 / 18
Gaussian-high	0 / 27	0 / 27	0 / 27	0 / 18	0 / 18	0 / 18	0 / 18	0 / 18	0 / 18
noiseless	0 / 27	0 / 27	0 / 27	0 / 18	0 / 18	0 / 18	0 / 18	0 / 18	0 / 18
sparse	0 / 27	0 / 27	0 / 27	0 / 18	0 / 18	0 / 18	0 / 18	0 / 18	0 / 18
dense	0 / 27	5 / 27	0 / 27	0 / 18	0 / 18	0 / 18	0 / 18	0 / 18	0 / 18
sparse-square	0 / 27	0 / 27	0 / 27	0 / 18	0 / 18	0 / 18	0 / 18	0 / 18	0 / 18
dense-square	0 / 27	1 / 27	0 / 27	0 / 18	0 / 18	0 / 18	0 / 18	0 / 18	0 / 18
K5	0 / 45	0 / 45	0 / 45	0 / 27	0 / 27	0 / 27	0 / 27	0 / 27	0 / 27
K10	0 / 45	1 / 45	0 / 45	0 / 27	0 / 27	0 / 27	0 / 27	0 / 27	0 / 27
K50	0 / 45	1 / 45	0 / 45	0 / 36	0 / 36	0 / 36	0 / 36	0 / 36	0 / 36
K70	0 / 45	0 / 45	0 / 45	0 / 36	0 / 36	0 / 36	0 / 36	0 / 36	0 / 36
large-K100	13 / 45	23 / 45	20 / 45	0 / 36	9 / 36	0 / 36	**36 / 36**	0 / 36	0 / 36
large-K400	18 / 45	23 / 45	25 / 45	0 / 36	9 / 36	9 / 36	**36 / 36**	7 / 36	9 / 36
**Total**	31 / 774	72 / 774	48 / 774	0 / 531	18 / 531	18 / 531	90 / 531	8 / 531	9 / 531

Table S8: Number of failed runs by each algorithm on each IMPC dataset. Failures are counted as runs that crashed, exceeded the time limit of 12 hours, exceeded the memory limit of 6GB or that failed to find any biclusters. Each cell displays the number of failures as a fraction of total number of attempted runs: *Failures/Attempts*. When all attempts failed, the counts are shown in bold.

	Adaptive			***NMF***		***Popular***		***Tensor***	
**Dataset**	SSLB	BicMix	BicMix-Q	nsNMF	SNMF	FABIA	Plaid	MultiCluster	SDA
Size factor	0 / 20	0 / 20	2 / 20	1 / 20	10 / 20	0 / 20	**20 / 20**	0 / 0	0 / 0
Log	0 / 20	4 / 20	4 / 20	0 / 20	7 / 20	0 / 20	0 / 20	0 / 0	0 / 0
Gaussian	0 / 20	0 / 20	3 / 20	0 / 0	0 / 0	0 / 20	10 / 20	0 / 0	0 / 0
Size factor (Tensor)	0 / 20	0 / 20	4 / 20	0 / 20	10 / 20	0 / 20	**20 / 20**	0 / 20	1 / 20
Log (Tensor)	0 / 20	0 / 20	3 / 20	0 / 20	7 / 20	0 / 20	0 / 20	0 / 20	0 / 20
Gaussian (Tensor)	0 / 20	0 / 20	3 / 20	0 / 0	0 / 0	0 / 20	4 / 20	0 / 20	0 / 20
**Total**	0 / 120	4 / 120	19 / 120	1 / 80	34 / 80	0 / 120	54 / 120	0 / 60	1 / 60

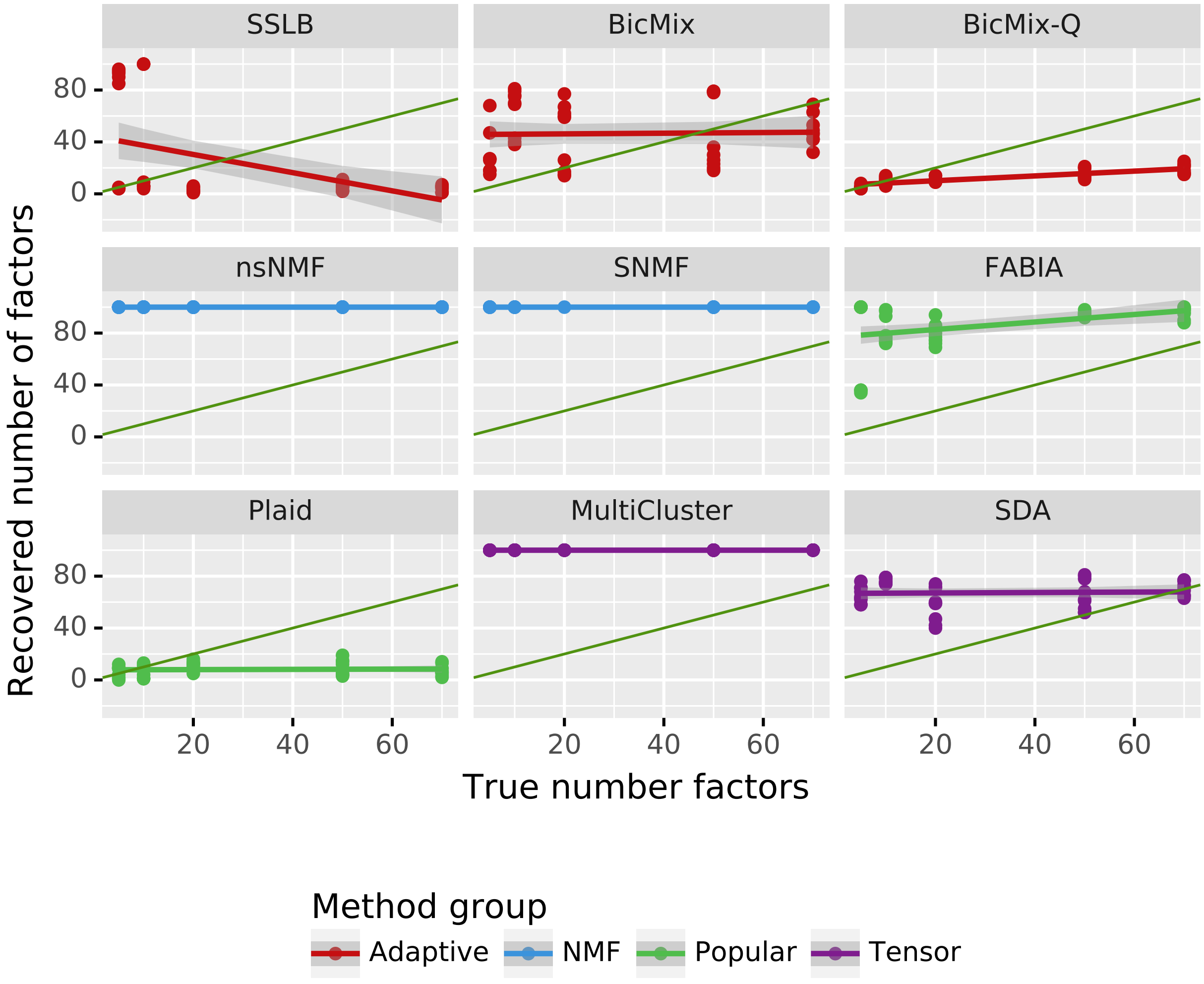

Figure S21: Recovery of K. Each algorithm is started with 100 biclusters on five datasets with varying number of implanted biclusters. K_{init} is chosen to be 100 as this larger than the true number of biclusters in any of the datasets. There is a point for each of the 3 seeds for each dataset of type *K5*, *K10*, *base* (which has $K = 20$), *K50* and *K70*. The number of biclusters recovered by the algorithm is plotted against the true number of biclusters. The standard thresholding procedure was performed as described in Section S6. The green line shows the ideal behaviour, with number of recovered biclusters matching the true number of biclusters exactly. None of the algorithms achieve this ideal behaviour, though FABIA and BicMix-Q show some positive correlation between the true number of biclusters and the recovered number of biclusters. A flatter line indicates poor sensitivity to the true number of biclusters, showing that the algorithm recovers the same number of biclusters irrespective of the true number of biclusters in the dataset. MultiCluster and the *NMF* algorithms returned 100 biclusters in all cases, showing they did not 'learn' K at all.

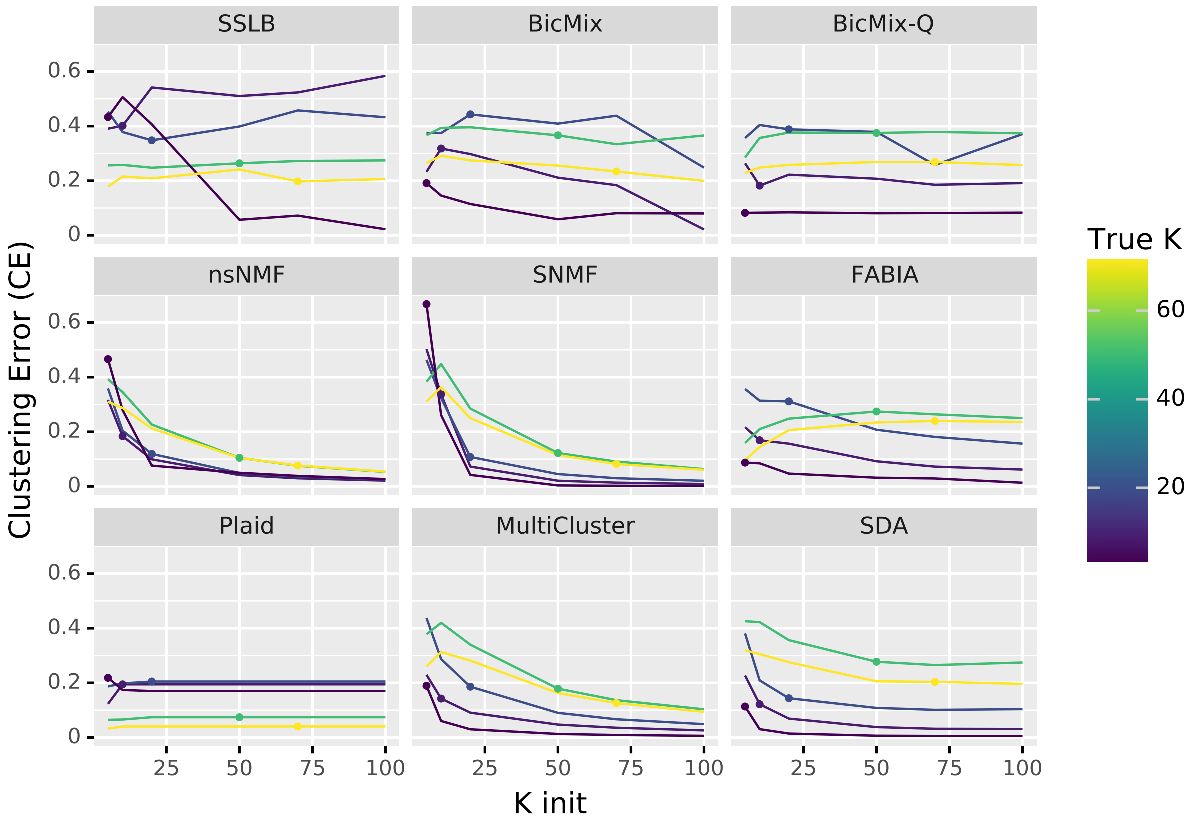

Figure S22: Biclustering accuracy (CE) of algorithms plotted against K_{init}. There is a line for each of dataset types *K5*, *K10*, *base*, *K50* and *K70*. For each dataset type there is a point at $K_{init} = K_{true}$ to draw attention to the performance of the algorithm at the true value of K. SNMF, nsNMF and MultiCluster have improved performance at low values of K_{init}, even when K_{true} is 50 or 70.

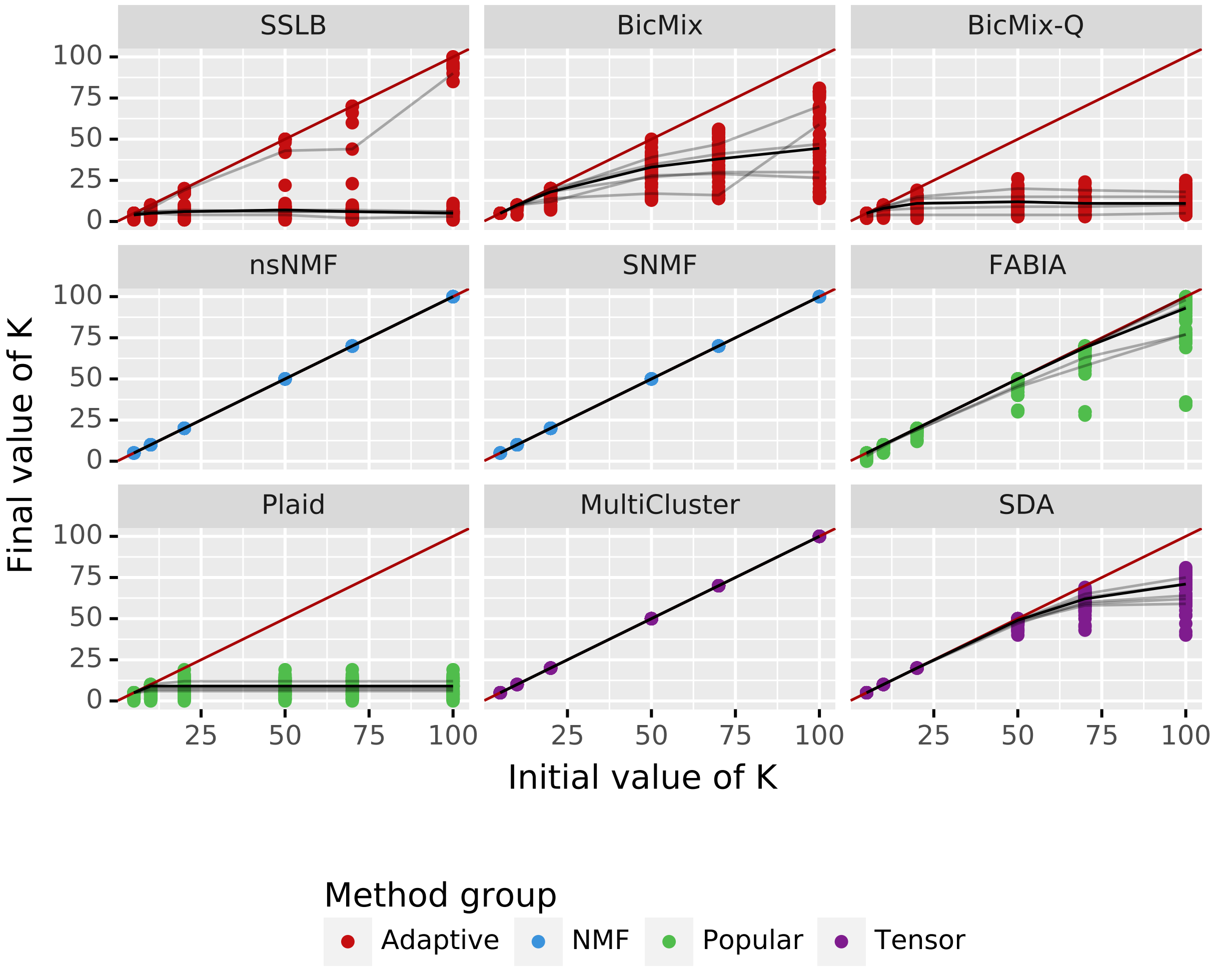

Figure S23: Robustness to choice of K_{init}, shown by final value of K (after post-processing) plotted against initial value of K. There is a point for each of the 3 seeds for each dataset of type *K5*, *K10*, *base*, *K50* and *K70*. The red line shows the limit, where the final value of K is the same as the initial value of K. The black line joins the median $\hat{K}$ across all datasets, and the gray lines join the medians for each individual dataset. The ideal behaviour is for the line to be flat once K_{init} exceeds the true K, showing that the algorithm converges to the same value of K regardless of the initial value given, as long as K_{init} is sufficiently large. True K varies from $K = 5$ to $K = 70$. MultiCluster and *NMF* algorithms always returned the same number of biclusters as they started with. Note with this plot that each algorithm shows a similar pattern in terms of recovered K, regardless of the true number of biclusters.

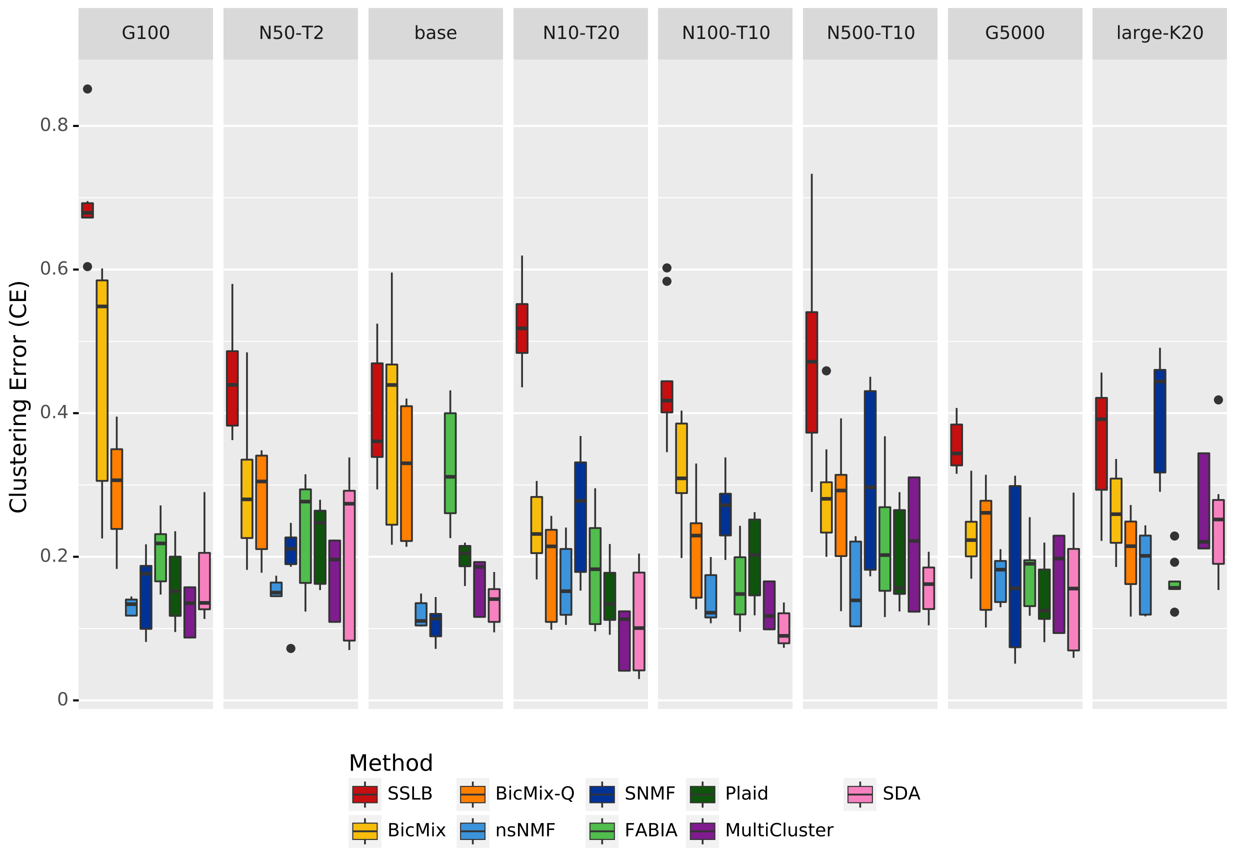

Figure S24: Biclustering accuracy (CE) of algorithms across datasets with a range of sizes. Note that Plaid failed to run on the *large-K20* dataset due to memory constraints. The bar shows median across all three runs on the thee random datasets of each type, with $K_{init} = K$ except for the *Adaptive* algorithms, and the standard thresholding applied.

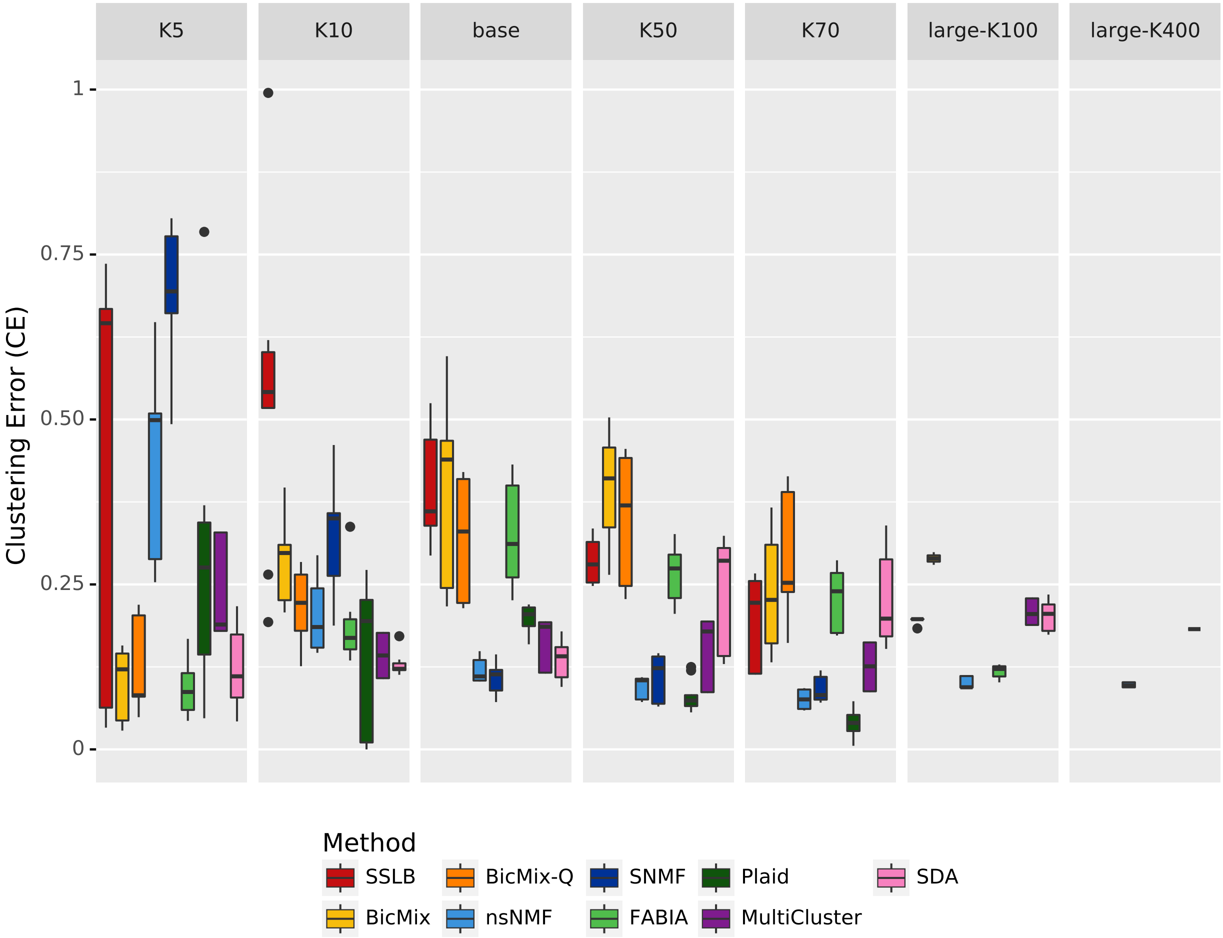

Figure S25: Biclustering accuracy (CE) of algorithms across datasets with different numbers of biclusters. The *base* dataset has 20 biclusters. The bar shows median across all runs on the random datasets of each type, with $K_{init} = K$ except for the *Adaptive* algorithms, and the standard thresholding applied.

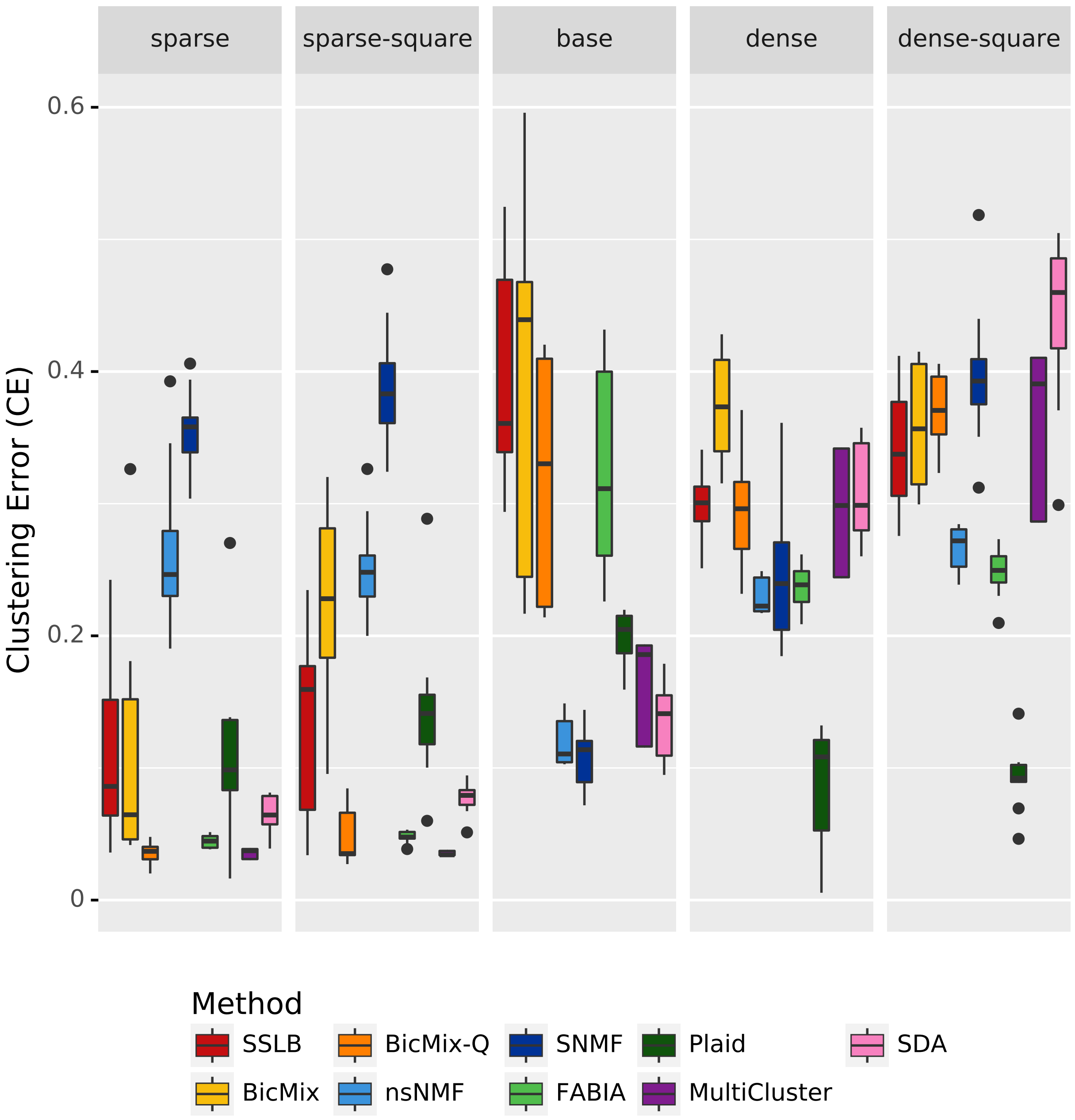

Figure S26: Biclustering accuracy (CE) of algorithms across datasets with different sparsity of biclusters. The distribution of bicluster sizes of the datasets is shown in the first row of Figures S19 and S20. The bar shows median across all runs on the random datasets of each type, with $K_{init} = K$ except for the *Adaptive* algorithms, and the standard thresholding applied.

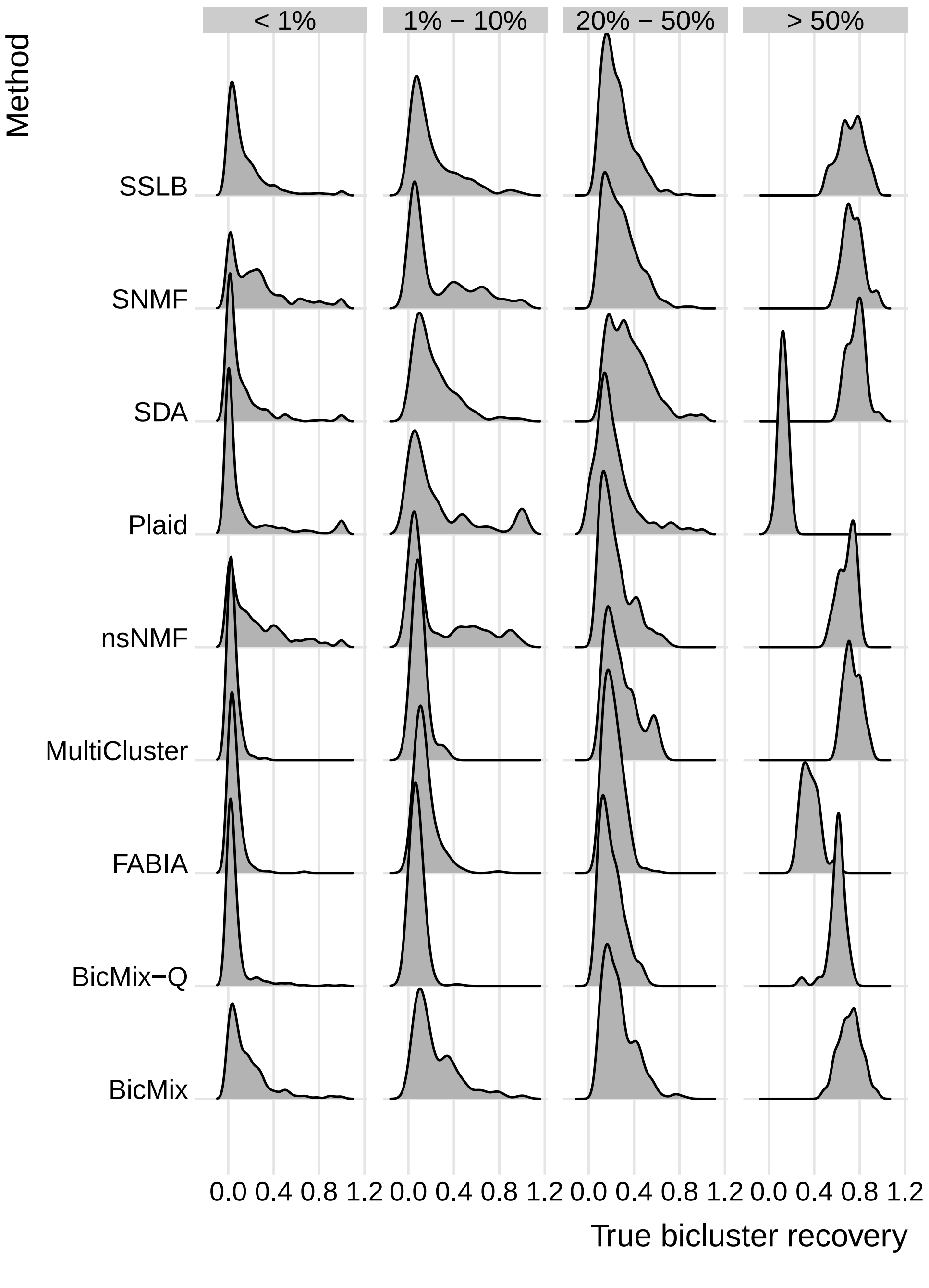

Figure S27: Recovery of true biclusters, grouped by size of true bicluster (proportion of total area taken up by true bicluster). Given true biclusters $A_1, A_2, \ldots, A_{20}$ and returned biclusters $B_1, B_2, \ldots, B_{\hat{K}}$, the recovery of a true bicluster A_k is $\max_l J(A_k, B_l)$ i.e. the maximum Jaccard index achieved between the true bicluster and some returned bicluster. The distribution of recovery scores for true biclusters from a range of datasets is shown, grouped by the size of true bicluster. The distribution of bicluster sizes of the datasets included is shown in the first row of Figures S19 and S20.

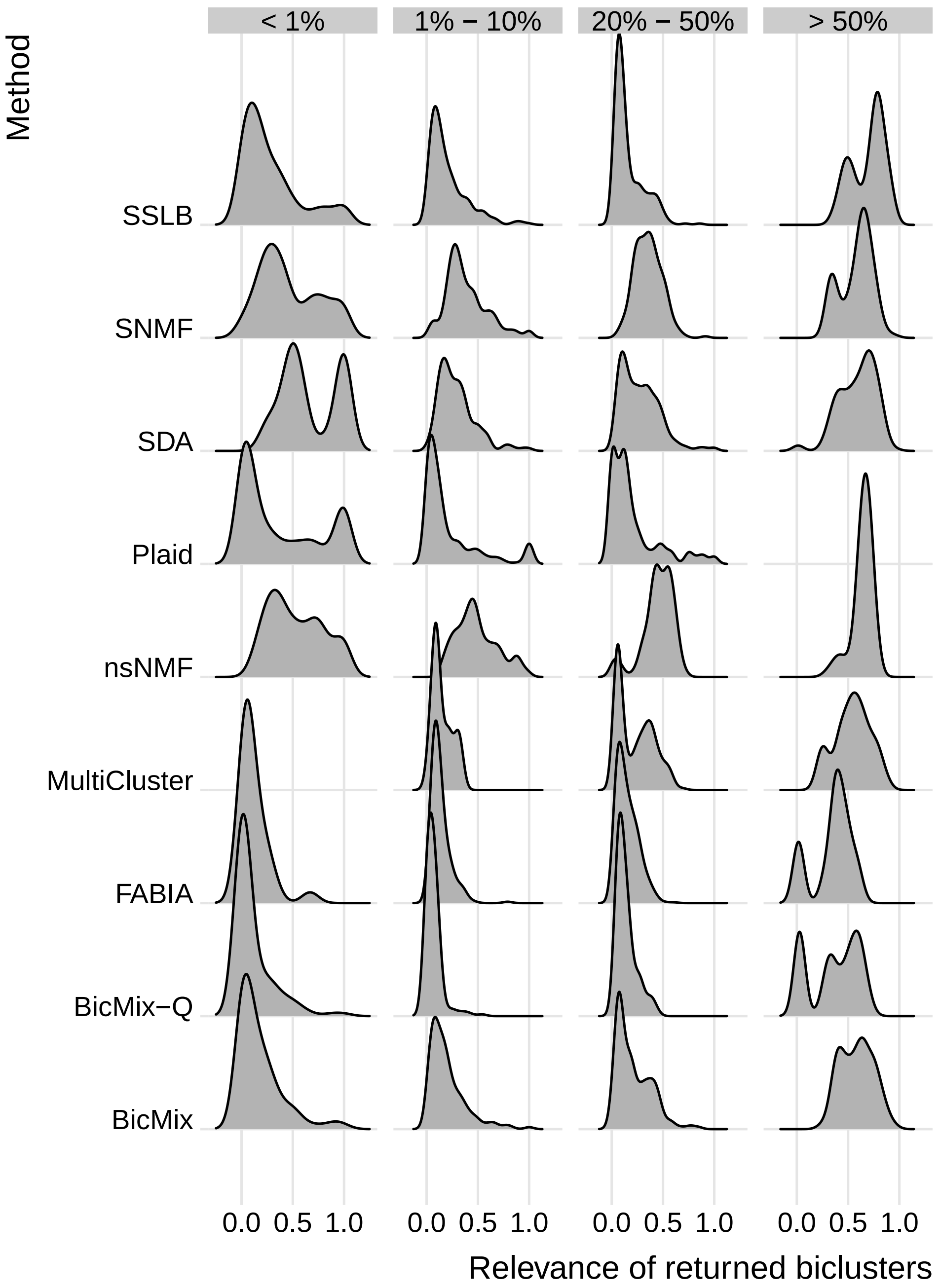

Figure S28: Relevance of returned biclusters, grouped by size of returned bicluster (proportion of total area taken up by returned bicluster). Given true biclusters $A_1, A_2, \ldots, A_{20}$ and returned biclusters $B_1, B_2, \ldots, B_{\hat{K}}$, the relevance of a returned bicluster B_l is $\max_k J(A_k, B_l)$ i.e. the maximum Jaccard index between the returned bicluster and some true bicluster. The distribution of recovery scores for true biclusters from a range of datasets is shown, grouped by the size of true bicluster. The distribution of bicluster sizes of the datasets included is shown in the first row of Figures S19 and S20.

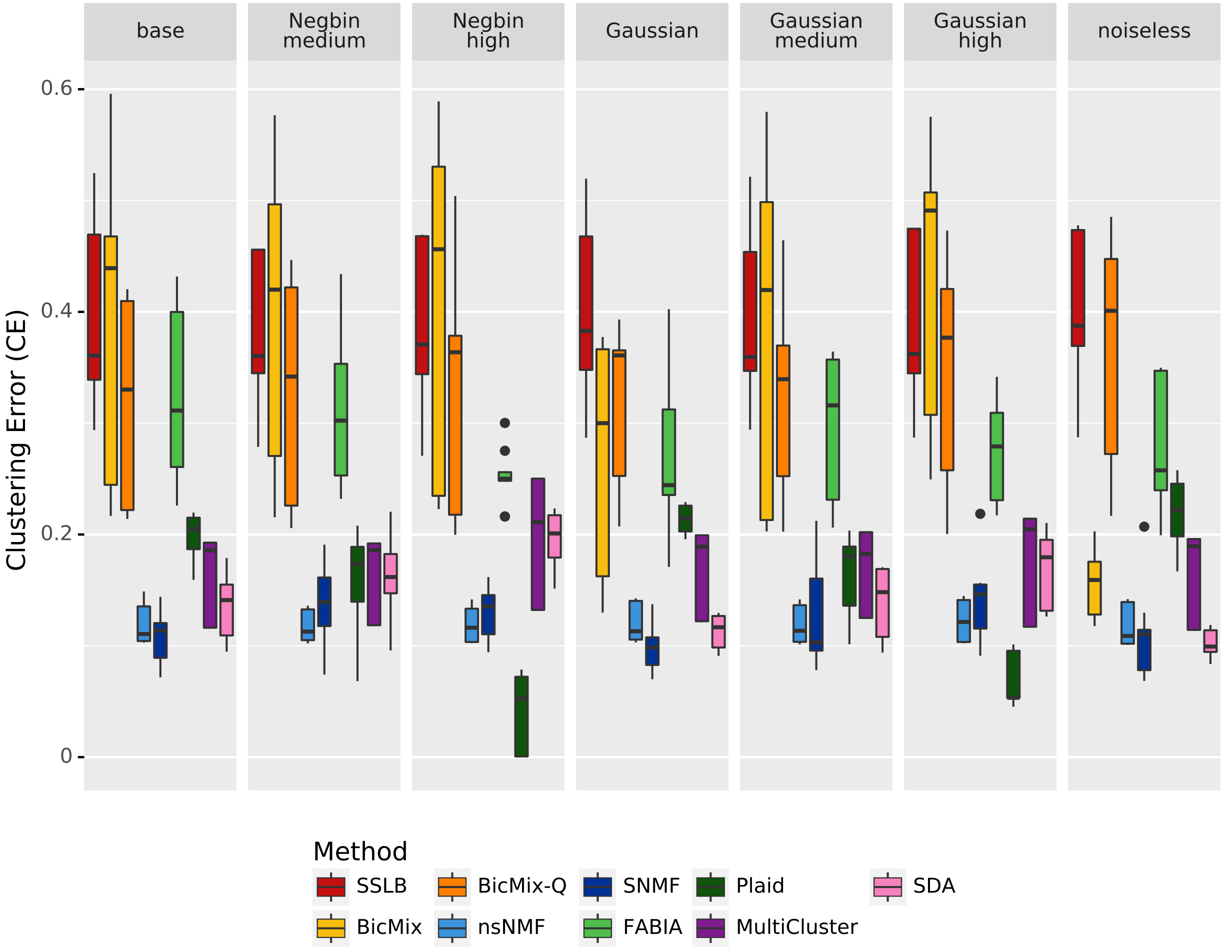

Figure S29: Biclustering accuracy (CE) of algorithms across datasets with different noise distribution. The *base* dataset uses Negative Binomial noise. The bar shows median across all runs on the random datasets of each type, with $K_{init} = K$ except for the *Adaptive* algorithms, and the standard thresholding applied.

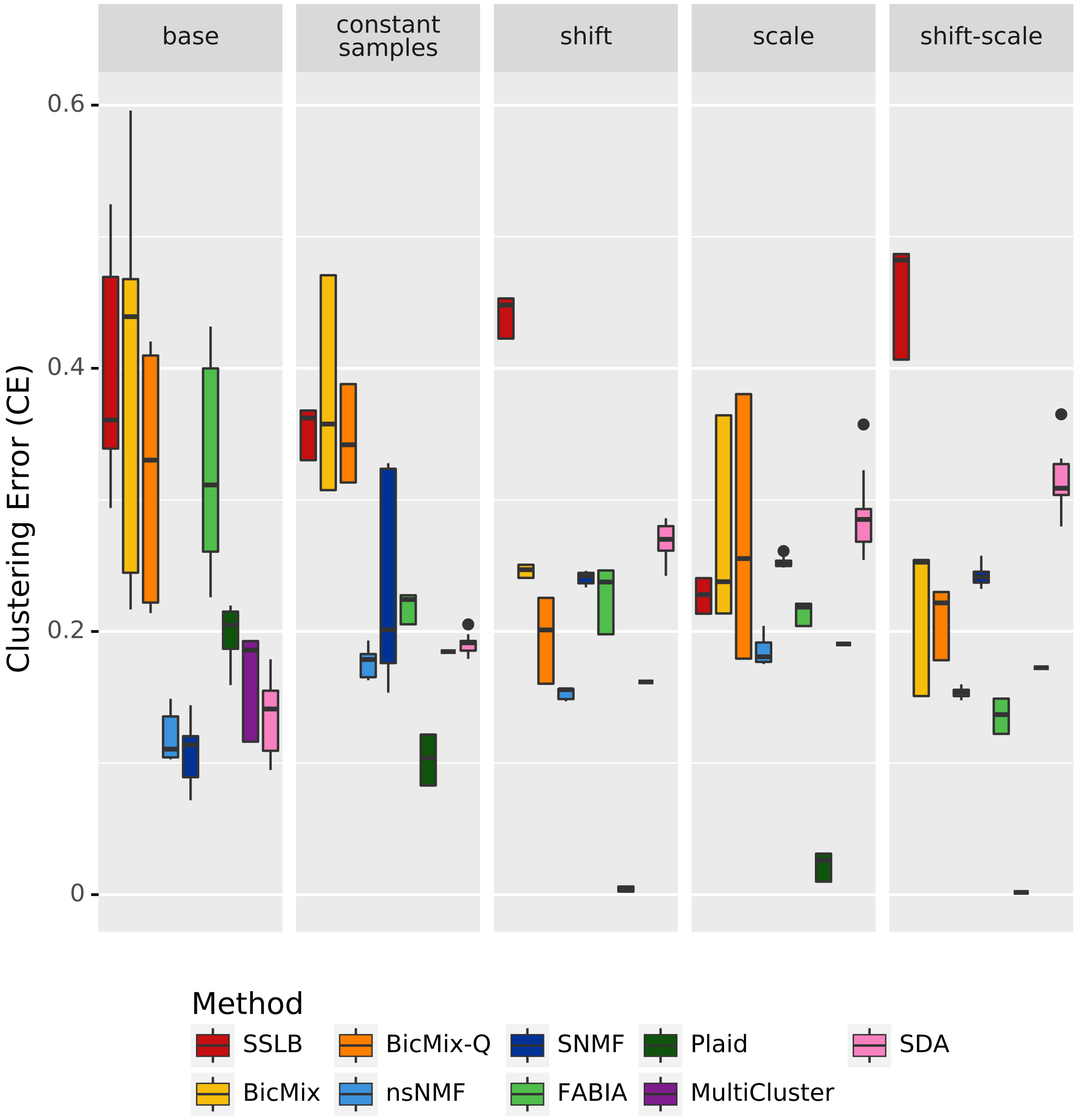

Figure S30: Biclustering accuracy (CE) of algorithms across datasets using different models for bicluster values. The *base* dataset uses the model used throughout the rest of the simulation study. The remaining datasets are variations on the shift-scale model described in [23] and in the main paper. The bar shows median across all three runs on the thee random datasets of each type, with $K_{init} = K$ except for the *Adaptive* algorithms, and the standard thresholding applied.

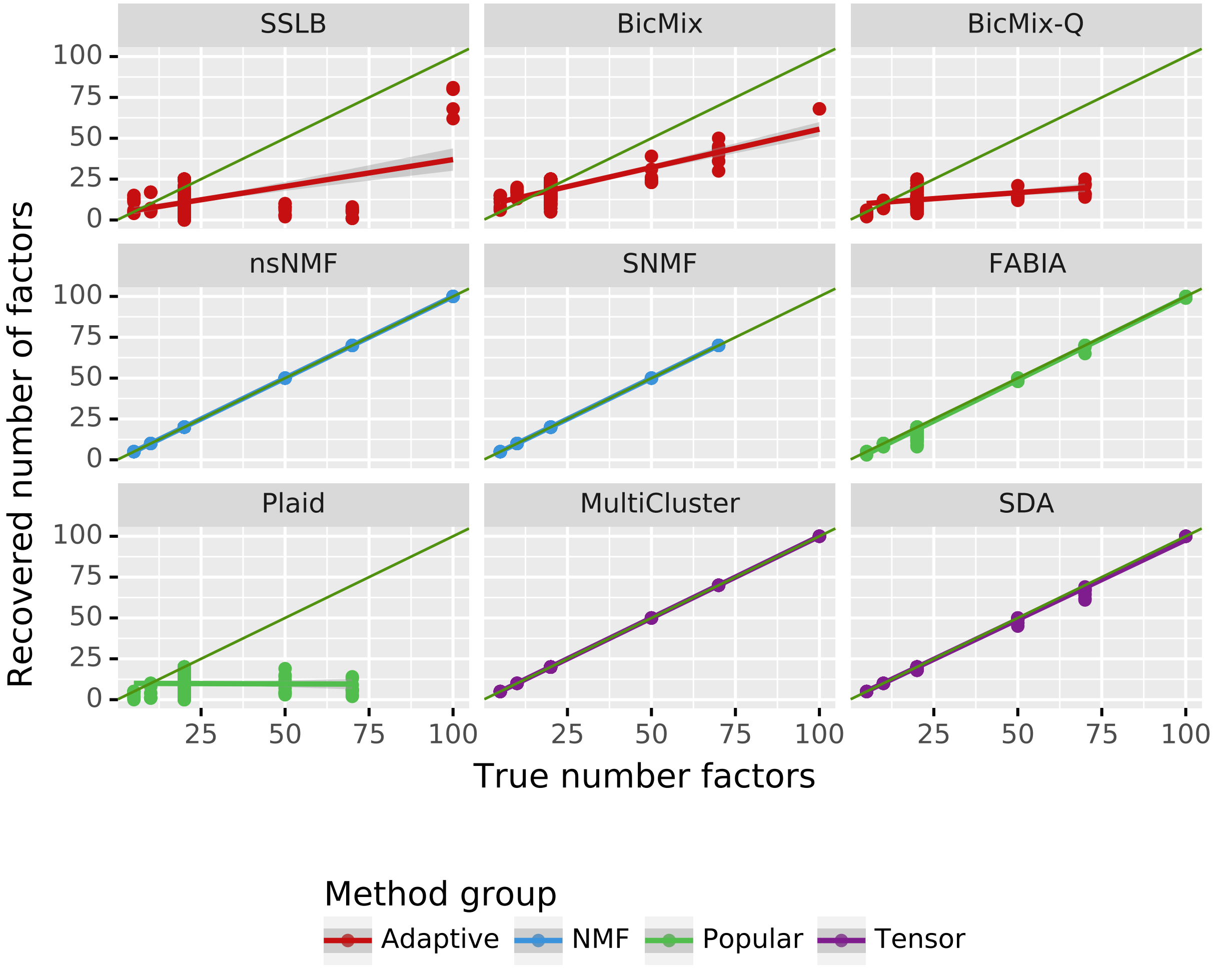

Figure S31: Recovery of K when algorithms are started with the value of K_{init} chosen for the study ($K_{\text{init}} = K$ for all algorithms except the *Adaptive* algorithms which use $K_{init} = K + 10$). There is a point for each of the 3 seeds for each dataset. The number of biclusters recovered by the algorithm is plotted against the true number of biclusters. The standard thresholding procedure was performed as described in Section S6. The green line shows the ideal behaviour, with number of recovered biclusters matching the true number of biclusters exactly. Note however that since all algorithms except the *Adaptive* algorithms started with the correct number of biclusters, it is not significant that many algorithms achieve this ideal behaviour.

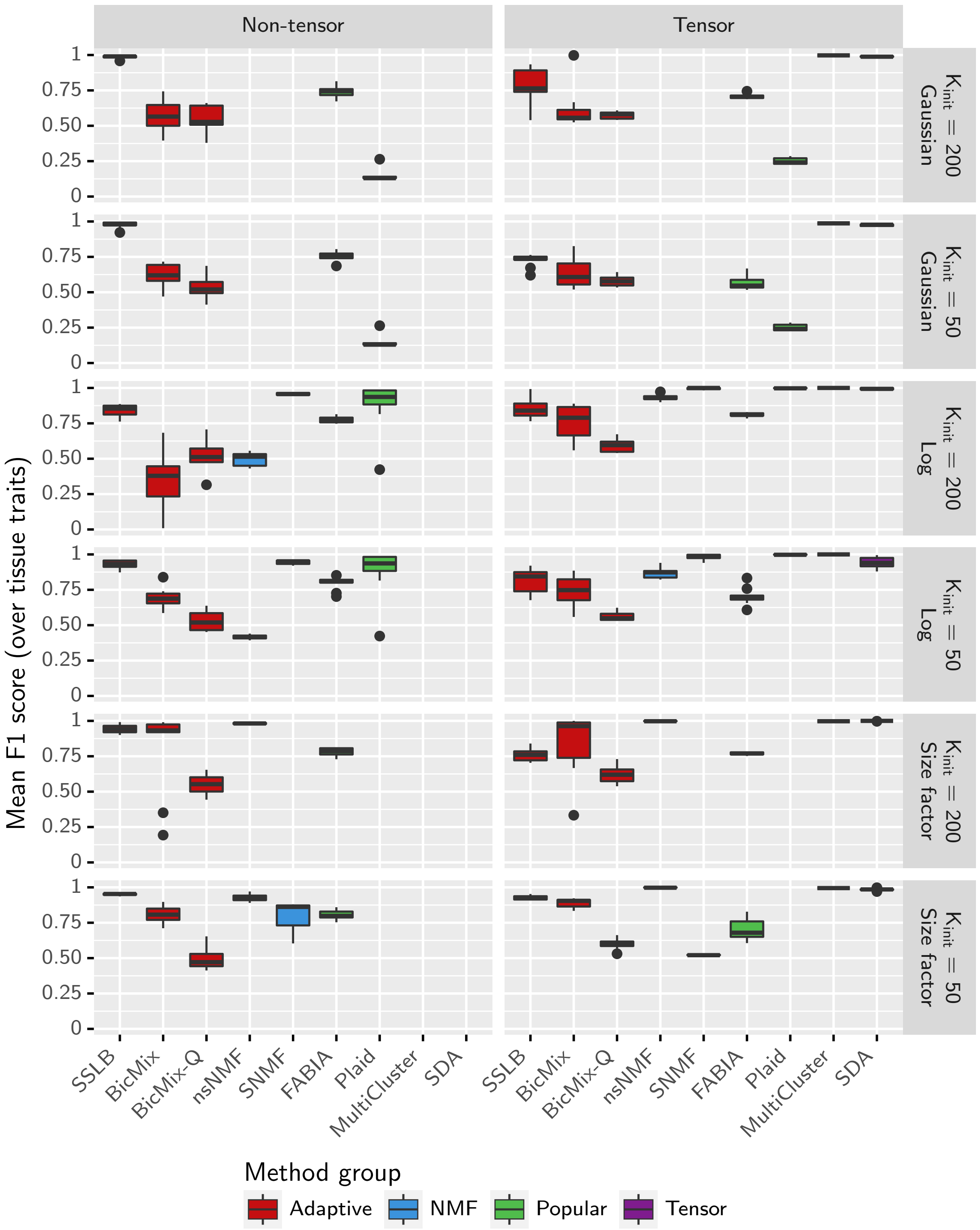

Figure S32: Sample clustering ability on IMPC datasets, measured by the mean over the tissue traits of the maximum F_1 score achieved by a bicluster. Standard thresholding is applied and results are shown both for $K_{init} = 50$ and $K_{init} = 200$. Note that MultiCluster and SDA couldn't be run on the non-tensor datasets, nsNMF and SNMF couldn't run on datasets using quantile normalisation and Plaid and SNMF failed to run on the DESeq dataset.

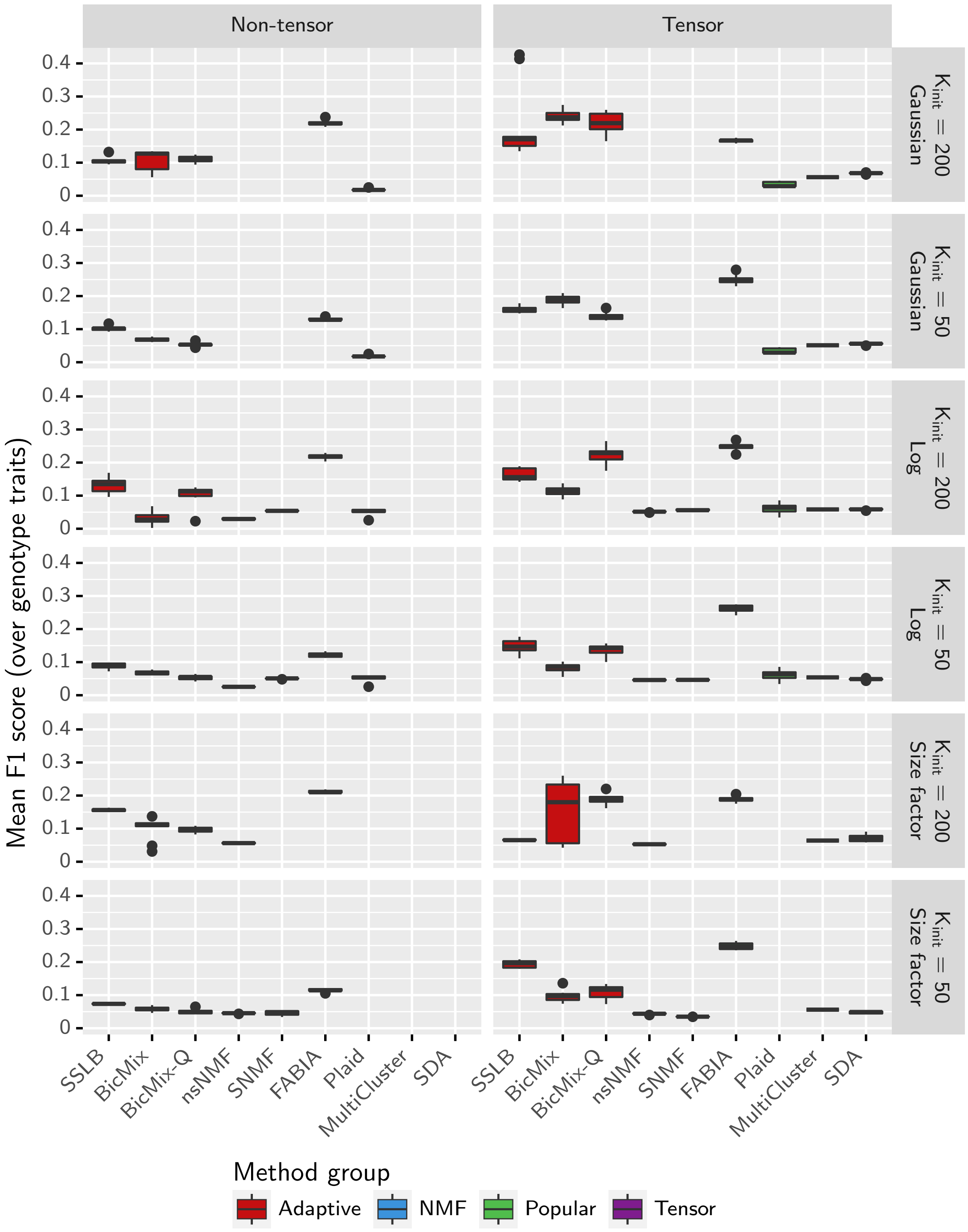

Figure S33: Sample clustering ability on IMPC datasets, measured by the mean over the genotype traits of the maximum F_1 score achieved by a bicluster. Standard thresholding is applied and results are shown both for $K_{init} = 50$ and $K_{init} = 200$. Note that MultiCluster and SDA couldn't be run on the non-tensor datasets, nsNMF and SNMF couldn't run on datasets using quantile normalisation and Plaid and SNMF failed to run on the DESeq dataset.

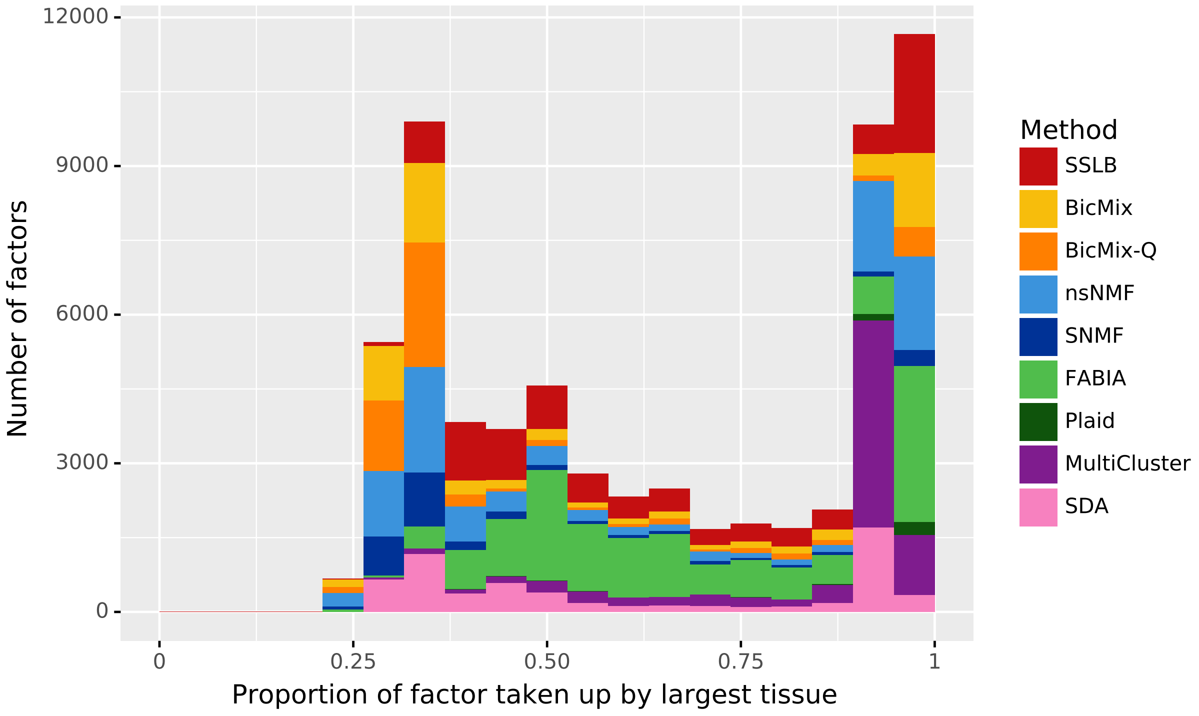

Figure S34: Proportion of bicluster taken up by the largest tissue contained in the bicluster, indicating how frequently the algorithms return biclusters containing multiple tissues. The proportion will be equal to 1 if a bicluster contains only samples from one tissue. The histogram shows that the majority of biclusters recovered contained samples mainly from a single tissue, rather than grouping together samples from different tissues. There is also a large spike around 0.29, which likely corresponds to biclusters containing every single sample as this is the proportion of samples that are from the liver, the most numerous tissue. All returned biclusters across all IMPC datasets are included in the count.

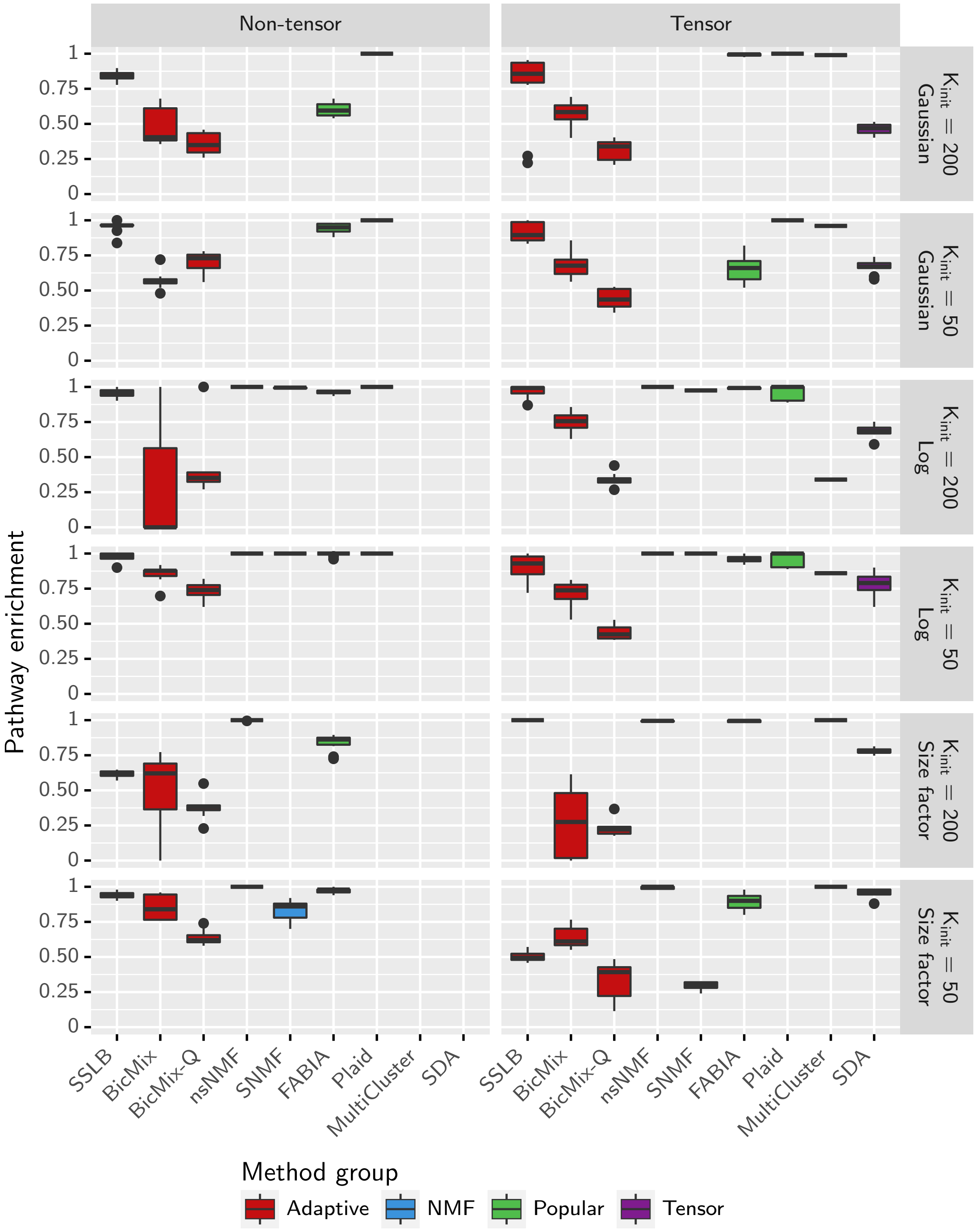

Figure S35: Gene clustering ability on IMPC datasets, measured by the mean proportion of recovered biclusters which are enriched for at least one pathway according to the one-tailed hypergeometric test adjusted for multiple testing using the Benjamini-Yekutieli correction, using a threshold for significance of 0.05. Standard thresholding is applied and results are shown both for $K_{init} = 50$ and $K_{init} = 200$. Note that MultiCluster and SDA couldn't be run on the non-tensor datasets, nsNMF and SNMF couldn't run on datasets using quantile normalisation and Plaid and SNMF failed to run on the DESeq dataset.

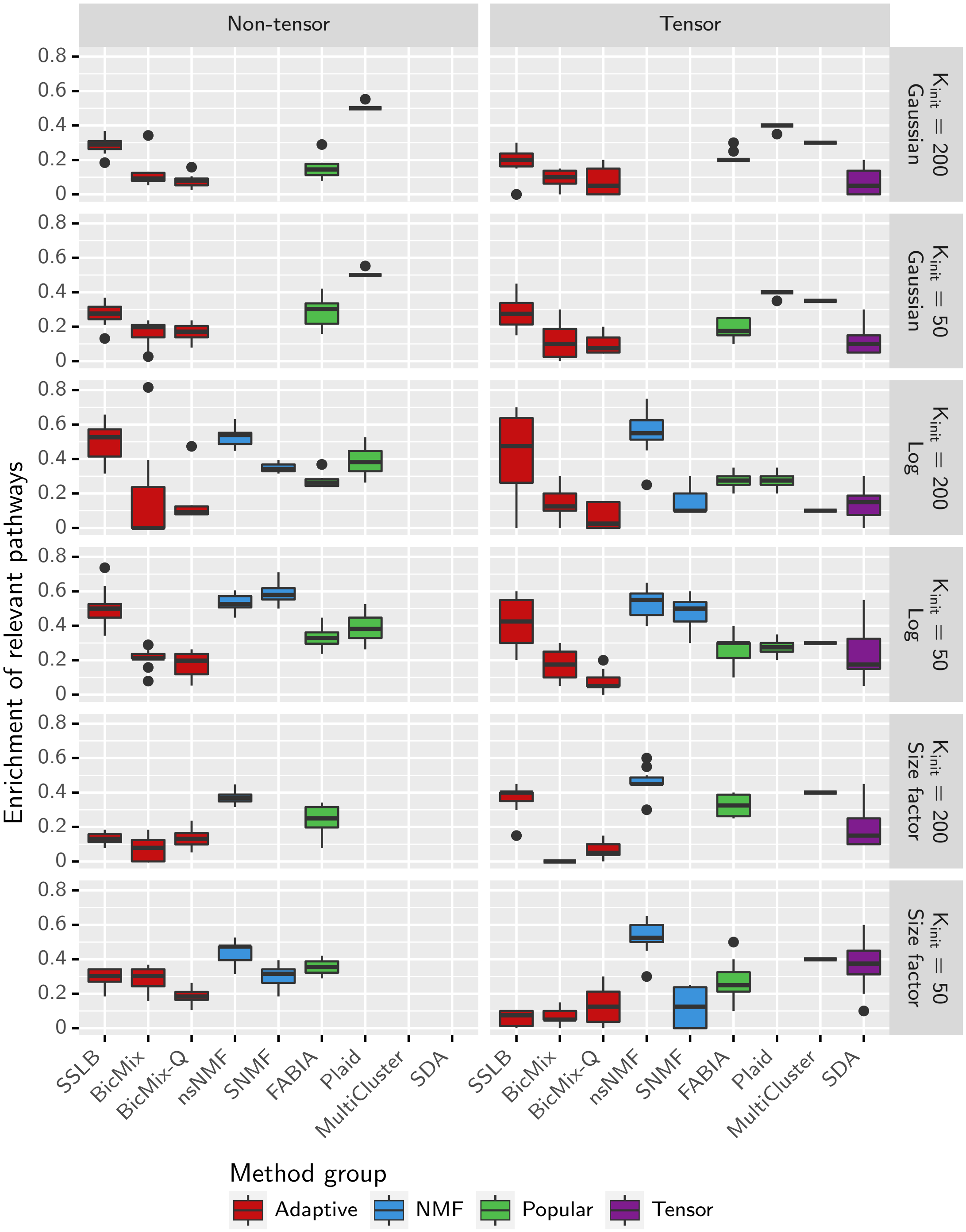

Figure S36: Biclustering ability on IMPC datasets, measured by the mean proportion of knocked-out genes for which the bicluster best matching the samples where the gene was knocked out is enriched for at least one pathway containing the knocked-out gene. Enrichment is measured using the one-tailed hypergeometric test adjusted for multiple testing using the Benjamini-Yekutieli correction, using a threshold for significance of 0.05. Standard thresholding is applied and results are shown both for $K_{init} = 50$ and $K_{init} = 200$. Note that *Tensor* algorithms couldn't be run on the non-tensor datasets, *NMF* algorithms couldn't run on datasets which used quantile normalisation and Plaid failed to run on the dataset which used DESeq's size factor normalisation.

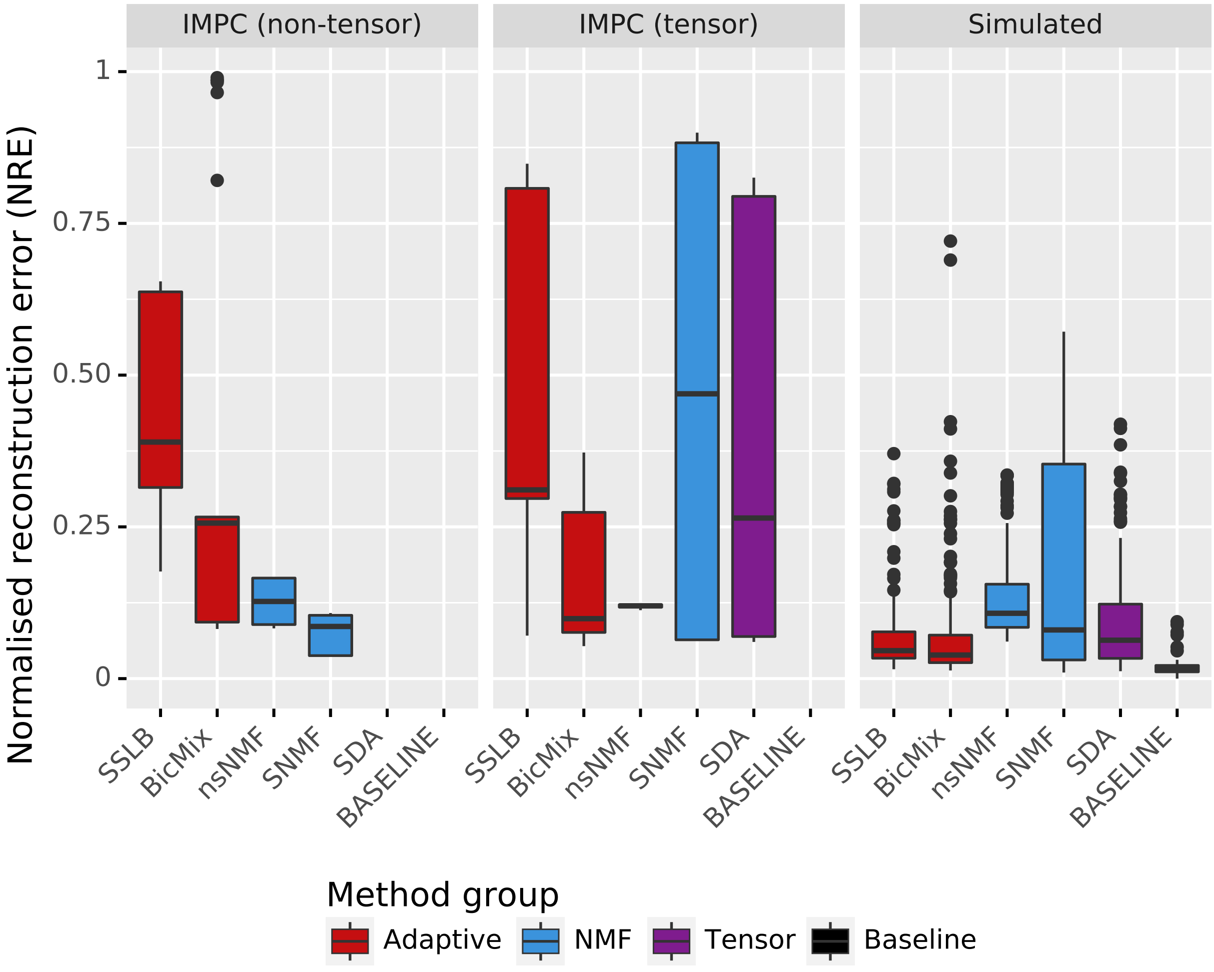

Figure S37: Normalised reconstruction error (NRE) across all datasets. Standard thresholding is applied. The best score is 0. For simulated datasets, BASELINE gives the NRE of the true factorisation $Y = XB^T + \varepsilon$, thus indicating the best performance possible.

	Cell type						Disease					MS Treatment		Lineage		Bicluster size	
	B-Cells	CD4	CD8	Monocytes	Neutrophils	Whole blood	ALS	MS	Sepsis	T1D	Healthy	Before IFN-β	After IFN-β	Myeloid	Lymphoid	Samples	Genes
0	11	17	11	11	12	8	12	24	11	12	11	12	12	23	39	70	421
4	11	6	7	8	8	5	5	11	8	12	9	4	7	16	24	45	3479
3	10	9	7	11	9	10	10	20	8	9	9	9	11	20	26	56	3382
6	10	7	6	11	9	6	6	13	9	10	11	5	8	20	23	49	3604
2	9	10	8	10	10	9	4	18	11	15	8	9	9	20	27	56	3428
5	7	10	10	7	9	10	9	18	8	7	11	11	7	16	27	53	3864
1	5	9	8	7	10	9	9	12	6	11	10	6	6	17	22	48	1268
10	5	1	3	5	4	7	1	9	4	7	4	3	6	9	9	25	2319
7	4	5	5	4	3	7	3	4	6	6	9	3	1	7	14	28	1275
8	3	5	4	3	2	5	3	8	3	6	2	6	2	5	12	22	11
9	1	3	5	2	5	3	3	6	4	2	4	2	4	7	9	19	3909
13	1	1	2		1	1		2	3	1		2		1	4	6	3816
12		1	1	1	2	1		1		2	3	1		3	2	6	2
11	1			1	3	3			2	1	5			4	1	8	390
14	2						2								2	2	3262
Total	20	20	20	20	20	20	18	36	18	24	24	18	18	40	60		

F1 score: 0.0 0.5 1.0

Figure S38: Summary of biclusters recovered by SSLB in the Benaroya sorted blood cell dataset after **log** transformation. Each row corresponds to a bicluster and each column corresponds to a sample trait. Each cell shows the number of samples contained in the bicluster which have the trait, and is coloured by the F1 score so that darker colours indicate that the bicluster is specific for that sample trait. *Myeloid* is the sum of the *Monocytes* and *Neutrophils* columns and *Lymphoid* is the sum of *B-Cells*, *CD4* and *CD8* columns. The *Samples* column shows the number of samples in the bicluster and *Genes* shows the number of genes contained in the bicluster.

Table S9: Summary of results on Benaroya RNA-seq dataset by algorithm and normalisation method. Each algorithm was run 10 times on each of the two normalised versions of the Benaroya dataset, all with $K\text{init} = 60$. The normalisation methods used were log transformation and size factor normalisation using DESeq's median of ratios normalisation method. For each normalisation method we show (A) the average number of biclusters returned, (B) how many of the 10 runs failed, and whether the algorithms returned biclusters (C) containing samples from every cell type, (D) strongly enriched for samples from myeloid cell types, (E) strongly enriched for samples from a single cell type and (F) enriched for samples from MS patients after treatment with IFN-beta.

		Adaptive			***NMF***		***Popular***		***Tensor***	
		SSLB	BicMix-Q	BicMix	nsNMF	SNMF	FABIA	Plaid	MultiCluster	SDA
	**Log**									
(A)	Average K recovered	42.3	21	53.8	60	60	60	2.2	60	60
(B)	Failing runs	0	9	1						
(C)	Cross-cell-type bicluster	Yes		Yes	Yes	Yes	Yes	Yes	Yes	Yes
(D)	Myeloid bicluster									
(E)	Single cell type bicluster									
(F)	IFN-beta bicluster									
	**Size factor**									
(A)	Average K recovered	59.4	34	60	60	60	60	1.6	60	60
(B)	Failing runs	0	5	0						
(C)	Cross-cell-type bicluster	Yes	Yes	Yes	Yes	Yes	Yes		Yes	Yes
(D)	Myeloid bicluster	Yes		Yes	Yes	Yes				Yes
(E)	Single cell type bicluster	Yes		Yes	Yes			Yes	Yes	
(F)	IFN-beta bicluster	Yes	Yes		Yes					

	Cell type						Disease					MS Treatment		Lineage		Bicluster size	
	B-Cells	CD4	CD8	Monocytes	Neutrophils	Whole blood	ALS	MS	Sepsis	T1D	Healthy	Before IFN-β	After IFN-β	Myeloid	Lymphoid	Samples	Genes
24	20	20	20	20	20	20	18	36	18	24	24	18	18	40	60	120	1935
28	20	20	20	20	20	20	18	36	18	24	24	18	18	40	60	120	1703
44	20	20	20	20	20	20	18	36	18	24	24	18	18	40	60	120	3565
49	20	20	20	20	20	20	18	36	18	24	24	18	18	40	60	120	1361
36	20	20	19	20	20	20	18	36	18	24	23	18	18	40	59	119	1428
31	20	20	19	17	19	19	18	34	18	22	22	17	17	36	59	114	1464
52	20	20	19	17	18	18	18	34	17	21	22	17	17	35	59	112	295
22	20	20	18	17	18	20	18	34	18	21	22	17	17	35	58	113	1523
18	20	19	18	20	20	20	18	35	17	24	23	18	17	40	57	117	308
32	19	20	20	18	20	16	17	35	18	22	21	18	17	38	59	113	846
30	19	20	20	17	20	19	18	34	18	24	21	17	17	37	59	115	492
8	19	20	19	18	19	18	17	34	16	22	24	17	17	37	58	113	1026
1	19	18	19	20	18	19	18	32	17	23	23	16	16	38	56	113	45
48	19	18	18	17	17	19	14	35	15	22	22	18	17	34	55	108	11
12	18	20	19	20	18	15	18	34	18	20	20	17	17	38	57	110	1760
33	18	20	19	19	17	17	16	33	16	22	23	17	16	36	57	110	27
41	18	19	16	16	18	17	18	28	14	21	23	15	13	34	53	104	117
39	18	18	18	19	18	19	16	33	18	21	22	16	17	37	54	110	62
5	18	17	18	19	17	18	14	31	16	24	22	15	16	36	53	107	1693
0	17	20	19	20	19	20	15	36	18	23	23	18	18	39	56	115	131
15	17	20	17	18	18	20	18	32	16	23	21	14	18	36	54	110	118
17	17	19	20	20	19	19	16	35	17	23	23	18	17	39	56	114	77
42	17	19	19	19	19	19	16	34	16	23	23	18	16	38	55	112	218
10	17	17	18	19	19	19	17	32	15	22	23	15	17	38	52	109	61
27	17	17	17	18	17	17	15	30	14	22	22	15	15	35	51	103	1358
4	17	16	19	17	17	18	16	33	15	21	19	17	16	34	52	104	563
6	17	16	18	16	15	16	14	29	13	20	22	15	14	31	51	98	1378
7	17	15	18	18	17	19	16	29	17	19	23	15	14	35	50	104	48
19	16	14	13	16	14	14	14	26	15	16	16	13	13	30	43	87	445
46	16	13	15	14	16	14	13	26	13	18	18	13	13	30	44	88	260
2	14	15	13	13	15	16	14	26	16	14	16	15	11	28	42	86	518
35	14	14	14	13	13	13	17	25	15	14	10	11	14	26	42	81	1192
25	13	16	17	17	15	15	11	27	14	21	20	12	15	32	46	93	655
9	13	16	17	15	13	15	13	27	13	17	19	14	13	28	46	89	465
23	13	14	15	13	14	16	12	27	12	17	17	12	15	27	42	85	323
50	13	14	14	16	19	18	16	26	15	20	17	12	14	35	41	94	242
13	13	12	14	13	13	13	11	20	11	19	17	11	9	26	39	78	592
43	13	11	14	11	10	11	13	22	12	12	11	10	12	21	38	70	326
40	13	10	18	12	11	13	15	22	13	14	13	11	11	23	41	77	480
38	11	13	15	13	17	11	14	20	13	20	13	12	8	30	39	80	215
21	11	9	12	9	9	10	13	19	10	10	8	9	10	18	32	60	391
34	11	4	6	6	5	6	3	10	5	11	9	5	5	11	21	38	129
14	10	4	3	5	8	6	7	12	7	5	5	5	7	13	17	36	88
51	9	15	14	13	13	11	9	22	9	17	18	11	11	26	38	75	445
45	9	5	9	7	8	8	10	15	9	6	6	6	9	15	23	46	1189
37	8	8	9	11	9	8	11	16	9	9	8	6	10	20	25	53	611
47	8	6	7	5	3	9	5	10	6	8	9	4	6	8	21	38	117
16	8	4	6	14	1	12	12	16	3	7	7	9	7	15	18	45	152
29	4	4	3	4	4	3	1	7	2	5	7	3	4	8	11	22	122
11	4	1	4	2	1	3	3	5		3	4	2	3	3	9	15	43
20	5	4	8	3	2		2	7	3	5	5	4	3	5	17	22	123
3	4		1	8		3	2	6	2	4	2	1	5	8	5	16	24
26		2	10	1			3	5	1	2	2	2	3	1	12	13	31
Total	20	20	20	20	20	20	18	36	18	24	24	18	18	40	60		

0.0 0.5 1.0
F1 score

Figure S39: Summary of biclusters recovered by BicMix in the Benaroya sorted blood cell dataset after **log** transformation. Each row corresponds to a bicluster and each column corresponds to a sample trait. Each cell shows the number of samples contained in the bicluster which have the trait, and is coloured by the F1 score so that darker colours indicate that the bicluster is specific for that sample trait. *Myeloid* is the sum of the *Monocytes* and *Neutrophils* columns and *Lymphoid* is the sum of *B-Cells*, *CD4* and *CD8* columns. The *Samples* column shows the number of samples in the bicluster and *Genes* shows the number of genes contained in the bicluster.

	Cell type						Disease					MS Treatment		Lineage		Bicluster size	
	B-Cells	CD4	CD8	Monocytes	Neutrophils	Whole blood	ALS	MS	Sepsis	T1D	Healthy	Before IFN-β	After IFN-β	Myeloid	Lymphoid	Samples	Genes
47	20	20	20	20	18	19	16	36	18	23	24	18	18	38	60	117	2745
15	20	20	19	18	19	20	18	35	17	23	23	18	17	37	59	116	2757
42	20	20	18	20	18	15	18	32	17	22	22	17	15	38	58	111	2829
58	20	20	18	18	16	20	16	34	17	22	23	16	18	34	58	112	2486
13	20	19	19	19	18	18	18	33	17	21	24	18	15	37	58	113	2832
38	20	18	20	20	19	17	16	33	18	24	23	18	15	39	58	114	2867
56	20	17	18	18	16	13	16	33	13	21	19	18	15	34	55	102	2476
49	20	17	13	20	20	18	16	32	15	24	21	17	15	40	50	108	3133
44	19	20	20	18	18	18	16	33	17	23	24	17	16	36	59	113	3078
51	19	20	19	20	20	17	17	33	17	24	24	17	16	40	58	115	3115
0	19	19	20	17	19	19	16	34	17	23	23	18	16	36	58	113	2531
27	19	19	19	19	18	18	18	33	18	21	22	17	16	37	57	112	3022
34	19	19	18	20	17	19	18	33	16	23	22	17	16	37	56	112	3101
25	19	19	18	19	18	19	17	34	18	22	21	17	17	37	56	112	3016
53	19	19	18	17	19	17	16	32	17	22	22	16	16	36	56	109	2953
22	19	19	16	18	18	16	17	30	18	20	21	15	15	36	54	106	2610
12	19	19	15	16	20	18	14	34	17	20	22	17	17	36	53	107	2998
36	19	18	19	20	18	18	18	34	15	22	23	17	17	38	56	112	2942
37	19	18	17	17	20	20	18	31	17	24	21	16	15	37	54	111	2559
19	19	16	17	19	19	19	17	32	16	22	22	16	16	38	52	109	2869
6	19	15	19	19	17	19	17	35	15	23	18	17	18	36	53	108	3074
55	18	20	19	19	16	16	16	31	16	23	22	14	17	35	57	108	2947
26	18	20	18	19	18	16	16	33	17	21	22	17	16	37	56	109	3070
16	18	19	19	18	18	20	18	30	18	23	23	13	17	36	56	112	2896
14	18	19	18	19	18	17	16	33	15	22	23	15	18	37	55	109	2973
45	18	19	17	19	16	19	16	33	14	24	21	16	17	35	54	108	2752
28	18	18	20	19	16	19	17	34	17	22	20	17	17	35	56	110	2942
46	18	18	19	18	20	19	16	36	17	21	22	18	18	38	55	112	3079
59	18	18	18	17	18	17	13	34	15	21	23	16	18	35	54	106	3140
10	18	18	17	20	19	17	15	36	14	22	22	18	18	39	53	109	2955
8	18	18	16	18	18	18	16	31	15	22	22	14	17	36	52	106	3033
23	18	17	20	19	20	17	17	34	18	21	21	18	16	39	55	111	3103
40	18	17	20	19	18	19	16	33	17	23	22	18	15	37	55	111	2817
31	18	17	17	20	15	19	16	32	15	21	22	17	15	35	52	106	3058
57	18	16	20	19	18	19	17	34	15	21	23	17	17	37	54	110	2923
50	18	16	19	18	17	17	15	32	18	18	22	15	17	35	53	105	2516
18	17	20	17	18	13	15	16	30	14	21	19	14	16	31	54	100	2269
43	17	19	19	16	18	20	18	31	17	20	23	16	15	34	55	109	3022
11	17	19	18	17	20	19	17	31	16	24	22	15	16	37	54	110	2885
24	17	19	18	16	16	19	17	32	14	21	21	16	16	32	54	105	2992
29	17	18	18	20	20	18	16	33	18	21	23	15	18	40	53	111	3045
1	17	17	19	18	17	18	14	33	13	22	24	16	17	35	53	106	2869
48	17	17	19	16	18	20	16	31	16	22	22	15	16	34	53	107	3144
33	17	17	18	19	17	18	16	34	12	22	22	16	18	36	52	106	2829
52	17	17	18	18	19	17	14	32	15	22	23	17	15	37	52	106	3115
3	17	17	17	18	18	18	14	32	16	21	22	17	15	36	51	105	3085
30	17	17	17	17	13	14	17	29	17	14	18	14	15	30	51	95	1961
4	17	17	17	16	16	18	16	30	15	21	19	16	14	32	51	101	3041
17	17	17	16	17	14	16	15	29	14	22	17	14	15	31	50	97	3118
2	17	13	18	17	18	19	15	30	16	20	21	15	15	35	48	102	3141
9	16	20	19	20	18	19	18	33	16	23	22	18	15	38	55	112	3086
32	16	16	13	17	15	15	11	26	13	21	21	13	13	32	45	92	2474
20	16	12	12	12	16	13	15	25	12	15	14	12	13	28	40	81	1767
54	15	19	18	18	19	16	15	34	17	21	18	17	17	37	52	105	2680
21	15	16	19	19	19	19	17	32	16	22	20	15	17	38	50	107	2491
41	15	16	16	17	18	17	13	28	16	20	22	14	14	35	47	99	3054
5	15	14	16	19	18	16	16	27	16	21	18	16	11	37	45	98	3013
7	15	14	14	18	15	14	13	26	13	17	21	12	14	33	43	90	2689
39	14	19	18	17	14	17	15	28	15	21	20	14	14	31	51	99	2946
35	13	10	14	11	11	9	13	21	14	10	10	11	10	22	37	68	1686
Total	20	20	20	20	20	20	18	36	18	24	24	18	18	40	60		

0.0 0.5 1.0
F1 score

Figure S40: Summary of biclusters recovered by nsNMF in the Benaroya sorted blood cell dataset after **log** transformation. Each row corresponds to a bicluster and each column corresponds to a sample trait. Each cell shows the number of samples contained in the bicluster which have the trait, and is coloured by the F1 score so that darker colours indicate that the bicluster is specific for that sample trait. *Myeloid* is the sum of the *Monocytes* and *Neutrophils* columns and *Lymphoid* is the sum of *B-Cells*, *CD4* and *CD8* columns. The *Samples* column shows the number of samples in the bicluster and *Genes* shows the number of genes contained in the bicluster.

	Cell type						Disease					MS Treatment		Lineage		Bicluster size	
	B-Cells	CD4	CD8	Monocytes	Neutrophils	Whole blood	ALS	MS	Sepsis	T1D	Healthy	Before IFN-β	After IFN-β	Myeloid	Lymphoid	Samples	Genes
0	20	20	20	20	20	20	18	36	18	24	24	18	18	40	60	120	3816
37	20	7	6	8	13	18	11	26	8	13	14	12	14	21	33	72	2118
16	18	16	6	8	16	11	8	21	12	20	14	10	11	24	40	75	2559
53	17	15	15	8	7	2	11	20	13	10	10	11	9	15	47	64	2155
19	16	12	14	8	14	8	10	22	12	15	13	12	10	22	42	72	2038
41	16	12	7	18	6	1	7	16	10	16	11	9	7	24	35	60	2390
11	16	5	6	10	5	15	7	14	14	14	8	8	6	15	27	57	2170
30	14	14	14	2	10	12	9	19	11	14	13	9	10	12	42	66	2300
2	14	14	8	8	8	6	11	19	4	11	13	9	10	16	36	58	2682
39	13	13	14	8	4	11	12	21	11	6	13	10	11	12	40	63	2082
58	13	10	6	8	9	7	11	14	7	9	12	6	8	17	29	53	2067
1	13	10	5	6	4	10	7	15	8	8	10	7	8	10	28	48	2904
55	13	2	4	12	5	6	4	14	3	11	10	6	8	17	19	42	2364
57	12	11	6	5	10	6	10	18	5	4	13	10	8	15	29	50	2379
14	12	4	14	18	6	6	7	18	11	14	10	10	8	24	30	60	2102
10	11	14	8	7	13	8	9	22	7	9	14	10	12	20	33	61	2603
59	11	13	8	12	10	6	9	18	5	14	14	9	9	22	32	60	2255
35	11	10	14	14	14	7	11	19	9	15	16	10	9	28	35	70	2202
20	11	4	14	17	3	1	7	15	4	15	9	9	6	20	29	50	2480
29	10	20	14	12	16	10	14	21	14	15	18	11	10	28	44	82	2545
36	10	8	7	11	9	1	7	15	5	7	12	8	7	20	25	46	1937
26	10	3	11	3	15	14	6	18	7	14	11	8	10	18	24	56	2623
51	10	3	5	9	8	7	6	13	5	8	10	7	6	17	18	42	2509
9	9	8	12	8	3	12	10	14	3	13	12	8	6	11	29	52	1880
44	9	3	2	4	7	9	4	12	5	4	9	7	5	11	14	34	2638
6	8	16	14	2	11	19	13	21	10	12	14	10	11	13	38	70	2170
15	8	15	7	14	14	8	14	18	5	11	18	9	9	28	30	66	1801
5	8	14	8	20	16	12	13	24	10	16	15	12	12	36	30	78	2799
33	8	10	1	6	10	19	9	14	8	13	10	7	7	16	19	54	2318
7	8	9	8	15	10	11	7	20	11	16	7	10	10	25	25	61	3030
28	8	3	18	2	3	10	9	14	8	4	9	7	7	5	29	44	2227
21	7	14	17	14	13	8	9	22	9	20	13	10	12	27	38	73	1924
3	7	14	8	13	11	7	10	18	6	10	16	9	9	24	29	60	2502
47	7	10	6	12	12	7	9	13	9	14	9	7	6	24	23	54	2112
49	7	3	2	14	13	9	8	14	5	9	12	8	6	27	12	48	2560
25	6	10	5	6	10	7	5	12	6	16	5	6	6	16	21	44	2678
43	6	9	8	5	16	11	8	14	7	14	12	6	8	21	23	55	2215
40	6	6	9	3	2	2	2	9	5	6	6	4	5	5	21	28	2733
17	6	6	6	12	10	14	7	13	12	12	10	7	6	22	18	54	2258
12	5	13	14	14	4	12	8	22	10	8	14	10	12	18	32	62	2429
18	5	10	12	7	13	2	3	16	8	11	11	7	9	20	27	49	2163
42	5	7	11	6	3	5	5	11	6	9	6	6	5	9	23	37	2868
34	5	2	5	1	3	12	10	8	1	5	4	5	3	4	12	28	2397
54	4	11	14	5	20	8	9	16	10	14	13	8	8	25	29	62	1914
22	4	9	4	6	9	11	5	15	6	11	6	6	9	15	17	43	2039
46	4	6	10	8	6	8	9	14	10	2	7	7	7	14	20	42	1892
24	2	7	10	4	10	1	6	9	4	8	7	4	5	14	19	34	2555
50	2	5	6	11	20	8	8	15	8	9	12	6	9	31	13	52	2591
38	2	4	12	16	10	8	8	16	6	12	10	8	8	26	18	52	2318
23	1	6	6	12	8	11	7	13	9	7	8	6	7	20	13	44	2203
8	12	13	12	14	12		6	19	10	12	16	10	9	26	37	63	2306
52	1	17	2	5	9		3	11	4	8	8	5	6	14	20	34	3003
45	18	10	11	5		11	7	19	7	13	9	9	10	5	39	55	2292
31	9	20	6	6		11	7	17	7	8	13	7	10	6	35	52	2159
48	8	4	8	8		13	5	13	7	8	8	7	6	8	20	41	2210
27	16	10	12		6	15	7	16	10	16	10	8	8	6	38	59	2151
4	6	4	18		10	16	8	16	9	12	9	8	8	10	28	54	2931
56	7	8		7	7	16	10	12	7	7	9	6	6	14	15	45	2417
13		16	8	12	6	11	10	16	8	8	11	8	8	18	24	53	2119
32		6	9	5	10	10	6	11	4	12	7	4	7	15	15	40	2539
Total	20	20	20	20	20	20	18	36	18	24	24	18	18	40	60		

0.0 0.5 1.0
F1 score

Figure S41: Summary of biclusters recovered by MultiCluster in the Benaroya sorted blood cell dataset after **log** transformation. Each row corresponds to a bicluster and each column corresponds to a sample trait. Each cell shows the number of samples contained in the bicluster which have the trait, and is coloured by the F1 score so that darker colours indicate that the bicluster is specific for that sample trait. *Myeloid* is the sum of the *Monocytes* and *Neutrophils* columns and *Lymphoid* is the sum of *B-Cells*, *CD4* and *CD8* columns. The *Samples* column shows the number of samples in the bicluster and *Genes* shows the number of genes contained in the bicluster.

	Cell type						Disease					MS Treatment		Lineage		Bicluster size	
	B-Cells	CD4	CD8	Monocytes	Neutrophils	Whole blood	ALS	MS	Sepsis	T1D	Healthy	Before IFN-β	After IFN-β	Myeloid	Lymphoid	Samples	Genes
0	20	20	20	20	20	20	18	36	18	24	24	18	18	40	60	120	835
14	20	20	20	20	20	20	18	36	18	24	24	18	18	40	60	120	1565
15	20	20	20	20	20	20	18	36	18	24	24	18	18	40	60	120	2840
17	20	20	20	20	20	20	18	36	18	24	24	18	18	40	60	120	1026
18	20	20	20	20	20	20	18	36	18	24	24	18	18	40	60	120	2418
28	20	20	20	20	20	20	18	36	18	24	24	18	18	40	60	120	2278
29	20	20	20	20	20	20	18	36	18	24	24	18	18	40	60	120	3522
35	20	20	20	20	20	20	18	36	18	24	24	18	18	40	60	120	1444
40	20	20	20	20	20	20	18	36	18	24	24	18	18	40	60	120	1679
44	20	20	20	20	20	20	18	36	18	24	24	18	18	40	60	120	3824
48	20	20	20	20	20	20	18	36	18	24	24	18	18	40	60	120	3605
21	20	20	20	20	15	13	16	32	17	21	22	16	16	35	60	108	1794
19	20	20	20	20	14	14	18	34	17	16	23	17	17	34	60	108	2568
56	20	20	20	20	10	18	16	34	17	20	21	17	17	30	60	108	784
52	20	20	20	20	10	12	16	32	14	20	20	16	16	30	60	102	1421
30	20	20	20	14	20	14	16	33	16	20	23	16	17	34	60	108	1674
5	20	20	20	14	14	14	14	30	16	22	20	15	15	28	60	102	244
16	20	20	14	20	20	20	18	34	15	24	23	17	17	40	54	114	604
38	20	20	14	20	20	20	18	34	18	20	24	17	17	40	54	114	865
7	20	20	14	17	20	20	15	33	17	24	22	17	16	37	54	111	2553
9	20	20	8	20	16	18	16	30	15	20	21	15	15	36	48	102	1001
39	20	16	18	14	20	20	16	32	17	22	21	16	16	34	54	108	3620
49	20	14	20	20	20	20	17	34	16	24	23	17	17	40	54	114	2250
12	20	14	20	20	14	20	18	36	16	16	22	18	18	34	54	108	3174
31	20	14	20	20	14	20	17	34	15	20	22	17	17	34	54	108	814
43	20	14	17	9	14	20	16	30	12	17	19	14	16	23	51	94	2795
1	20	14	14	20	20	20	14	32	16	24	22	16	16	40	48	108	477
6	20	14	8	20	20	14	15	30	14	16	21	15	15	40	42	96	1906
8	19	19	20	17	20	20	18	33	18	24	22	17	16	37	58	115	2305
13	19	19	19	14	19	14	11	33	16	24	20	15	18	33	57	104	2101
47	19	14	15	12	17	19	13	28	15	20	20	13	15	29	48	96	3109
53	18	17	12	18	16	17	13	29	15	20	21	13	16	34	47	98	2795
34	18	16	20	20	20	20	17	33	18	24	22	17	16	40	54	114	1071
25	18	16	20	20	16	18	15	31	18	24	20	16	15	36	54	108	1836
33	18	16	14	20	20	14	17	29	18	16	22	15	14	40	48	102	1314
3	18	15	13	13	10	12	13	21	12	19	16	12	9	23	46	81	3342
24	18	12	18	20	20	20	15	31	18	24	20	16	15	40	48	108	4037
45	17	12	17	12	13	16	14	28	11	15	19	12	16	25	46	87	2255
37	16	17	17	15	13	17	16	27	16	20	16	15	12	28	50	95	2569
10	15	16	18	11	16	11	14	24	17	16	16	13	11	27	49	87	3318
2	14	20	20	20	20	20	18	34	18	22	22	17	17	40	54	114	2576
22	14	20	20	20	20	20	18	34	18	22	22	17	17	40	54	114	2497
42	14	20	20	20	20	20	18	34	18	22	22	17	17	40	54	114	2842
51	14	20	20	20	20	14	18	32	14	22	22	16	16	40	54	108	821
23	14	20	20	20	14	20	15	33	14	24	22	16	17	34	54	108	2106
20	14	20	20	18	16	14	17	29	17	18	21	15	14	34	54	102	747
55	14	20	20	18	10	20	16	29	16	22	19	15	14	28	54	102	1662
41	14	20	19	18	17	14	11	32	15	24	20	16	16	35	53	102	2187
54	14	14	20	20	20	14	18	32	17	14	21	16	16	40	48	102	1823
57	14	14	20	12	16	20	15	30	15	18	18	15	15	28	48	96	846
46	14	14	14	14	20	14	12	27	11	22	18	13	14	34	42	90	1116
26	14	11	16	13	10	17	11	22	13	16	19	11	11	23	41	81	2150
27	13	18	18	20	20	20	17	32	16	22	22	17	15	40	49	109	2422
36	13	17	16	17	13	16	13	29	12	17	21	13	16	30	46	92	2717
32	12	17	13	17	15	6	10	24	11	22	13	12	12	32	42	80	3002
4	12	16	20	20	20	20	17	31	18	22	20	16	15	40	48	108	386
11	12	16	20	20	20	20	17	31	18	22	20	16	15	40	48	108	1964
50	12	16	20	18	16	20	14	29	16	24	19	15	14	34	48	102	3339
Total	20	20	20	20	20	20	18	36	18	24	24	18	18	40	60		

0.0 0.5 1.0
F1 score

Figure S42: Summary of biclusters recovered by SDA in the Benaroya sorted blood cell dataset after **log** transformation. Each row corresponds to a bicluster and each column corresponds to a sample trait. Each cell shows the number of samples contained in the bicluster which have the trait, and is coloured by the F1 score so that darker colours indicate that the bicluster is specific for that sample trait. *Myeloid* is the sum of the *Monocytes* and *Neutrophils* columns and *Lymphoid* is the sum of *B-Cells*, *CD4* and *CD8* columns. The *Samples* column shows the number of samples in the bicluster and *Genes* shows the number of genes contained in the bicluster.

	Cell type						Disease					MS Treatment		Lineage		Bicluster size	
	B-Cells	CD4	CD8	Monocytes	Neutrophils	Whole blood	ALS	MS	Sepsis	T1D	Healthy	Before IFN-β	After IFN-β	Myeloid	Lymphoid	Samples	Genes
4	20	20	18	20	20	15	17	36	16	21	23	18	18	40	58	113	3153
59	20	17	18	18	20	16	16	35	16	21	21	18	17	38	55	109	2914
46	19	18	19	18	19	16	18	31	17	20	23	17	14	37	56	109	3235
54	19	17	13	17	15	17	15	32	15	19	17	16	16	32	49	98	2879
10	19	15	10	19	16	13	12	28	14	21	17	12	16	35	44	92	1502
48	19	8	18	13	8	12	13	21	12	21	11	12	9	21	45	78	2696
8	18	19	15	18	19	17	16	31	17	21	21	16	15	37	52	106	3196
43	18	18	17	14	19	9	15	28	15	16	21	15	13	33	53	95	3427
16	18	18	15	18	18	19	15	33	15	22	21	16	17	36	51	106	2756
24	18	15	15	19	17	16	16	33	13	20	18	17	16	36	48	100	3367
41	17	17	15	18	17	12	13	29	14	20	20	14	15	35	49	96	3513
42	17	16	15	19	17	16	14	29	13	22	22	15	14	36	48	100	2913
26	17	14	11	18	18	18	14	30	16	20	16	14	16	36	42	96	3231
55	17	13	13	17	10	11	12	24	10	19	16	12	12	27	43	81	3571
1	16	19	18	19	20	15	15	34	15	22	21	18	16	39	53	107	3270
36	16	19	15	17	14	13	15	27	15	18	19	12	15	31	50	94	3362
25	16	17	17	15	17	16	10	31	14	22	21	16	15	32	50	98	3403
13	16	16	17	12	12	20	18	27	13	17	18	14	13	24	49	93	3189
20	15	17	19	17	19	16	15	33	15	20	20	17	16	36	51	103	3368
2	15	17	11	17	12	17	11	28	14	17	19	16	12	29	43	89	3163
18	15	15	14	17	19	16	14	28	14	21	19	13	15	36	44	96	3330
19	15	15	9	16	16	11	11	23	11	21	16	13	10	32	39	82	3411
31	15	12	10	12	16	16	11	25	13	15	17	11	14	28	37	81	3129
0	15	10	10	8	14	8	10	23	6	8	18	13	10	22	35	65	3240
22	15	10	9	12	12	17	8	20	11	17	19	10	10	24	34	75	3599
14	14	15	14	16	8	15	14	28	11	14	15	13	15	24	43	82	3246
50	14	13	7	9	17	7	6	19	10	18	14	11	8	26	34	67	2907
57	13	15	8	14	15	13	11	22	13	17	15	14	8	29	36	78	2938
37	13	8	13	13	14	5	9	17	9	13	18	10	7	27	34	66	3419
33	13	7	12	19	7	11	9	22	8	16	14	10	12	26	32	69	2621
53	12	14	10	14	19	16	11	26	13	17	18	14	12	33	36	85	2860
38	12	11	9	8	4	13	8	18	8	10	13	8	10	12	32	57	3282
51	12	8	7	4	8	4	4	15	5	7	12	8	7	12	27	43	3097
29	12	5	4	6	5	8	8	12	9	5	6	7	5	11	21	40	2637
28	11	14	12	14	10	12	8	21	8	18	18	11	10	24	37	73	3402
40	11	8	13	14	7	9	8	20	9	12	13	10	10	21	32	62	3352
52	11	6	4	6	6	6	7	12	8	7	5	5	7	12	21	39	2229
30	10	12	8	9	10	7	10	15	12	9	10	9	6	19	30	56	3363
9	10	8	7	10	6	10	6	16	5	12	12	7	9	16	25	51	2808
49	10	7	10	6	6	6	7	16	8	8	6	7	9	12	27	45	2084
5	10	3	18	16	19	14	8	27	11	18	16	15	12	35	31	80	3058
15	9	15	18	20	13	20	12	26	15	21	21	13	13	33	42	95	2828
27	9	9	12	7	9	7	13	19	9	7	5	8	11	16	30	53	2404
56	9	8	7	8	9	8	6	15	5	10	13	8	7	17	24	49	2830
47	9	7	14	10	8	14	14	18	9	12	9	9	9	18	30	62	2506
58	9	7	7	5	4	3	5	11	9	6	4	5	6	9	23	35	2936
35	8	9	6	10	5	7	9	13	9	7	7	6	7	15	23	45	2200
44	8	9	5	9	6	9	4	13	3	15	11	6	7	15	22	46	1645
45	8	7	12	4	7	4	7	18	4	8	5	9	9	11	27	42	2840
34	8	5	3	4	4	6	3	9	3	9	6	5	4	8	16	30	2005
12	7	7	1	4	13	14	8	18	3	6	11	9	9	17	15	46	2386
7	7	3	5	6	5	6	6	10	5	5	6	5	5	11	15	32	2187
32	7	2	3	4	2	3	4	7	3	4	3	3	4	6	12	21	1969
39	6	6	9	7	5	7	6	14	10	5	5	6	8	12	21	40	2857
21	5	10	10	10	8	6	5	14	4	17	9	6	8	18	25	49	3481
17	4	8	5	7	8	5	1	10	2	13	11	6	4	15	17	37	2968
23	4	6	10	13	13	12	7	19	6	12	14	11	8	26	20	58	3153
3	3	5	3	3	4	4	4	8	6	3	1	3	5	7	11	22	2636
6	3	1	7	2	1	2	4	4	3	3	2	2	2	3	11	16	1905
11		8	4	1	13	9	5	5	8	13	4	3	2	14	12	35	2784
Total	20	20	20	20	20	20	18	36	18	24	24	18	18	40	60		

0.0 0.5 1.0
F1 score

Figure S43: Summary of biclusters recovered by SNMF in the Benaroya sorted blood cell dataset after **log** transformation. Each row corresponds to a bicluster and each column corresponds to a sample trait. Each cell shows the number of samples contained in the bicluster which have the trait, and is coloured by the F1 score so that darker colours indicate that the bicluster is specific for that sample trait. *Myeloid* is the sum of the *Monocytes* and *Neutrophils* columns and *Lymphoid* is the sum of *B-Cells*, *CD4* and *CD8* columns. The *Samples* column shows the number of samples in the bicluster and *Genes* shows the number of genes contained in the bicluster.

	Cell type						Disease					MS Treatment		Lineage		Bicluster size	
	B-Cells	CD4	CD8	Monocytes	Neutrophils	Whole blood	ALS	MS	Sepsis	T1D	Healthy	Before IFN-β	After IFN-β	Myeloid	Lymphoid	Samples	Genes
23	20	20	19	18	17	18	16	34	17	23	22	18	16	35	59	112	3247
30	20	19	19	18	19	17	16	33	18	22	23	17	16	37	58	112	2884
58	20	19	19	18	18	18	18	34	15	23	22	18	16	36	58	112	3136
19	20	19	18	17	15	19	16	33	15	22	22	15	18	32	57	108	3196
11	20	19	17	19	14	20	18	33	17	20	21	16	17	33	56	109	3193
55	20	19	17	16	20	19	17	33	16	22	23	17	16	36	56	111	3090
17	20	19	17	16	18	15	16	30	15	20	24	15	15	34	56	105	2793
52	20	19	17	15	19	17	16	33	17	20	21	16	17	34	56	107	3209
50	20	18	20	15	18	18	17	31	18	20	23	15	16	33	58	109	2776
29	20	18	19	18	20	19	18	33	18	21	24	15	18	38	57	114	3569
12	20	18	17	20	19	20	18	35	17	22	22	18	17	39	55	114	3253
32	20	17	19	19	18	17	15	33	17	22	23	16	17	37	56	110	3122
41	19	20	19	19	20	20	17	35	18	23	24	17	18	39	58	117	3357
7	19	20	17	19	14	18	16	32	18	21	20	16	16	33	56	107	3300
44	19	19	19	17	18	17	15	34	17	23	20	17	17	35	57	109	3098
31	19	19	18	19	18	17	18	32	16	21	23	16	16	37	56	110	2666
36	19	19	18	18	18	18	16	35	15	23	21	17	18	36	56	110	3282
18	19	19	17	19	20	18	16	36	16	21	23	18	18	39	55	112	3254
51	19	18	18	19	20	20	17	33	17	23	24	16	17	39	55	114	3316
0	19	18	18	17	20	19	17	32	18	22	22	16	16	37	55	111	2870
2	19	18	17	20	18	17	16	32	17	22	22	15	17	38	54	109	2912
46	19	18	17	19	19	17	17	32	17	22	21	14	18	38	54	109	3701
59	19	18	17	18	20	15	17	32	15	24	19	16	16	38	54	107	3230
48	19	18	17	18	19	18	15	34	16	21	23	17	17	37	54	109	3383
25	19	18	16	15	16	19	13	33	15	23	19	15	18	31	53	103	3050
40	19	17	18	19	17	18	17	32	15	22	22	17	15	36	54	108	3148
39	19	17	18	15	19	18	15	32	16	22	21	16	16	34	54	106	3139
53	19	17	17	19	20	19	18	32	16	24	21	16	16	39	53	111	3041
14	19	17	17	17	19	16	15	33	15	22	20	16	17	36	53	105	3289
37	19	16	20	20	18	18	18	32	16	23	22	17	15	38	55	111	3444
1	18	20	20	17	17	19	18	33	15	23	22	16	17	34	58	111	2949
22	18	20	18	15	20	17	16	32	17	23	20	16	16	35	56	108	3230
54	18	20	17	15	19	17	14	32	14	22	24	16	16	34	55	106	3208
35	18	20	16	16	20	18	17	32	16	22	21	15	17	36	54	108	3140
5	18	19	19	18	16	16	16	32	15	23	20	16	16	34	56	106	2991
24	18	19	19	16	18	19	17	31	17	21	23	15	16	34	56	109	3067
56	18	18	19	19	20	19	18	35	18	21	21	18	17	39	55	113	3181
13	18	18	19	18	18	20	18	31	17	24	21	15	16	36	55	111	3308
45	18	18	19	18	16	17	17	33	16	19	21	18	15	34	55	106	3228
27	18	18	17	17	18	20	13	35	18	19	23	18	17	35	53	108	3016
16	18	17	20	17	19	17	15	32	17	22	22	16	16	36	55	108	2592
6	18	17	18	16	18	20	18	34	12	22	21	18	16	34	53	107	2948
21	18	17	17	20	18	18	15	32	18	21	22	18	14	38	52	108	3337
34	18	16	19	16	20	18	16	34	14	21	22	17	17	36	53	107	3525
47	17	19	19	15	16	17	17	31	16	19	20	16	15	31	55	103	3015
10	17	19	16	19	19	19	15	35	15	22	22	17	18	38	52	109	3232
33	17	18	17	18	20	18	15	35	16	20	22	17	18	38	52	108	2997
9	17	16	18	20	18	17	13	33	16	22	22	18	15	38	51	106	3264
57	17	15	18	18	20	18	17	30	17	22	20	14	16	38	50	106	3283
4	16	20	20	17	19	20	17	33	17	22	23	18	15	36	56	112	2973
26	16	19	19	20	16	18	17	32	17	20	22	16	16	36	54	108	3196
28	16	19	18	17	17	19	16	32	16	20	22	16	16	34	53	106	3015
8	16	17	15	19	19	18	17	31	16	19	21	15	16	38	48	104	3365
3	16	17	15	19	18	18	17	28	16	21	21	12	16	37	48	103	3366
38	15	19	18	19	20	19	18	33	17	23	19	16	17	39	52	110	2764
20	15	18	19	20	17	19	17	35	17	21	18	18	17	37	52	108	2889
43	15	17	17	17	18	19	16	33	15	22	17	17	16	35	49	103	3105
42	15	17	16	17	19	16	15	31	14	18	22	15	16	36	48	100	3134
49	15	16	18	19	19	19	17	29	16	22	22	13	16	38	49	106	3372
15	14	20	18	17	20	18	17	30	16	22	22	17	13	37	52	107	3240
Total	20	20	20	20	20	20	18	36	18	24	24	18	18	40	60		

F1 score: 0.0 0.5 1.0

Figure S44: Summary of biclusters recovered by FABIA in the Benaroya sorted blood cell dataset after **log** transformation. Each row corresponds to a bicluster and each column corresponds to a sample trait. Each cell shows the number of samples contained in the bicluster which have the trait, and is coloured by the F1 score so that darker colours indicate that the bicluster is specific for that sample trait. *Myeloid* is the sum of the *Monocytes* and *Neutrophils* columns and *Lymphoid* is the sum of *B-Cells*, *CD4* and *CD8* columns. The *Samples* column shows the number of samples in the bicluster and *Genes* shows the number of genes contained in the bicluster.

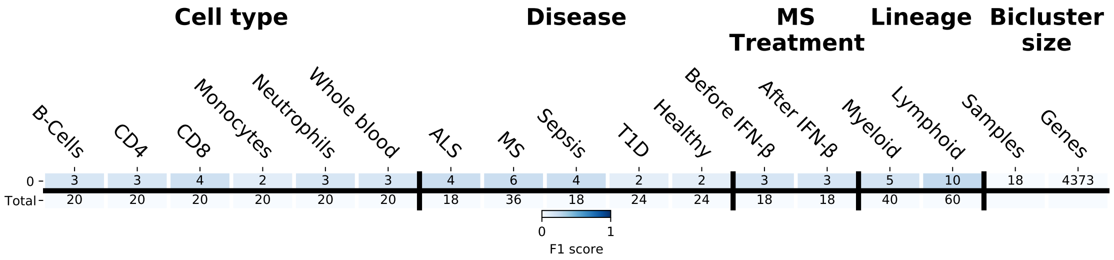

Figure S45: Summary of biclusters recovered by Plaid in the Benaroya sorted blood cell dataset after **log** transformation. Each row corresponds to a bicluster and each column corresponds to a sample trait. Each cell shows the number of samples contained in the bicluster which have the trait, and is coloured by the F1 score so that darker colours indicate that the bicluster is specific for that sample trait. *Myeloid* is the sum of the *Monocytes* and *Neutrophils* columns and *Lymphoid* is the sum of *B-Cells*, *CD4* and *CD8* columns. The *Samples* column shows the number of samples in the bicluster and *Genes* shows the number of genes contained in the bicluster.

	Cell type						Disease					MS Treatment		Lineage		Bicluster size	
	B-Cells	CD4	CD8	Monocytes	Neutrophils	Whole blood	ALS	MS	Sepsis	T1D	Healthy	Before IFN-β	After IFN-β	Myeloid	Lymphoid	Samples	Genes
0	20	20	20	20	20	20	18	36	18	24	24	18	18	40	60	120	898
4	5	4	4	2	4	20	9	11	6	6	7	5	6	6	13	39	798
11	3	3	2	2	7	3		15		2	3		15	9	8	20	255
1	2	1	3	20	20	9	7	14	12	10	12	6	8	40	6	55	894
5	15	11	13	5		7	10	11	5	12	13	4	7	5	39	51	305
7	8	12	14	4		7	9	11	7	7	11	5	6	4	34	45	310
30	1	3	1	1		2	1	2		5		1	1	1	5	8	2
9	15	1	1			2	2	6	3	5	3	3	3		17	19	320
12	2	4	10			3	3	5	2	3	6	2	3		16	19	46
22	1	1	1			6	3	4		1	1	1	3		3	9	76
16	1		1	1		11		4	1	5	4	3	1	1	2	14	25
21	1			1	4	1			6		1			5	1	7	173
2			1	3	20	18	5	11	10	8	8	6	5	23	1	42	814
15	1	8	5				3	2	5	2	2	1	1		14	14	84
42	2	1		1					1	2	1			1	3	4	24
23	3			1		1	1	3			1	2	1	1	3	5	37
8		18	10			3	3	9	4	8	7	5	4		28	31	227
6		10	19			7	7	11	4	8	6	8	3		29	36	427
20		8	2			2	1	7	2	1	1	4	3		10	12	17
19		1	1			11	3	5	1	2	2	3	2		2	13	88
26			1	2	3				6					5	1	6	479
14			1		4	5		1	7		2		1	4	1	10	470
3				4	17	6	4	8	2	6	7	4	4	21		27	645
17				1	7	3	2	3	1	2	3	2	1	8		11	436
39				1	2	1		2		2		1	1	3		4	381
47				1	1	2		4				3	1	2		4	72
10	19					2	3	6	2	4	6	3	3		19	21	662
53	1					1			2						1	2	212
25		3	4					3			4		3		7	7	19
55		1				1				2					1	2	34
13				7		7		7		2	5	4	3	7		14	59
24				7		1		5			3	2	3	7		8	88
18				6		3		2	4		3	1	1	6		9	109
28				5		1	2	2		1	1	1	1	5		6	70
54				2		1			1		2			2		3	12
40				1		5		2		1	3	1	1	1		6	40
44				1		2			1		2			1		3	69
49				1		1		1	1			1		1		2	56
29					4	2		2			4	1	1	4		6	56
32					3	1	1		1		2			3		4	90
35					3	1	2	1			1		1	3		4	66
41					2	2	1	2			1	2		2		4	69
31					2	1				1	2			2		3	68
36					2	1		1			2	1		2		3	71
45					2	1	2				1			2		3	58
46					1	3			1	2	1			1		4	49
50					1	1			2					1		2	166
52					1	1				2				1		2	55
33	6							1	3	2		1			6	6	32
27	4						1	2		1		1	1		4	4	29
56	2						1			1					2	2	46
43			2						1		1				2	2	18
37				3				1			2		1	3		3	13
51				2					2					2		2	121
34					3		1	1			1		1	3		3	150
38					3			1			2	1		3		3	66
48						4	1				3					4	13
Total	20	20	20	20	20	20	18	36	18	24	24	18	18	40	60		

0.0 0.5 1.0
F1 score

Figure S46: Summary of biclusters recovered by SSLB in the Benaroya sorted blood cell dataset after **size factor** normalisation using DESeq's median of ratios method. Each row corresponds to a bicluster and each column corresponds to a sample trait. Each cell shows the number of samples contained in the bicluster which have the trait, and is coloured by the F1 score so that darker colours indicate that the bicluster is specific for that sample trait. *Myeloid* is the sum of the *Monocytes* and *Neutrophils* columns and *Lymphoid* is the sum of *B-Cells*, *CD4* and *CD8* columns. The *Samples* column shows the number of samples in the bicluster and *Genes* shows the number of genes contained in the bicluster.

	Cell type						Disease					MS Treatment		Lineage		Bicluster size	
	B-Cells	CD4	CD8	Monocytes	Neutrophils	Whole blood	ALS	MS	Sepsis	T1D	Healthy	Before IFN-β	After IFN-β	Myeloid	Lymphoid	Samples	Genes
5	20	20	20	20	20	20	18	36	18	24	24	18	18	40	60	120	275
6	20	20	20	20	20	20	18	36	18	24	24	18	18	40	60	120	369
27	20	20	20	20	20	20	18	36	18	24	24	18	18	40	60	120	430
32	20	20	20	20	20	20	18	36	18	24	24	18	18	40	60	120	220
35	20	20	20	20	20	20	18	36	18	24	24	18	18	40	60	120	126
40	20	20	20	20	20	20	18	36	18	24	24	18	18	40	60	120	271
21	20	20	20	20	20	19	18	36	17	24	24	18	18	40	60	119	166
54	20	20	20	20	20	19	18	36	18	23	24	18	18	40	60	119	337
11	20	20	20	20	19	20	18	35	18	24	24	17	18	39	60	119	110
37	20	20	20	20	19	18	18	36	17	24	22	18	18	39	60	117	254
55	20	20	20	20	16	20	17	35	18	23	23	17	18	36	60	116	324
50	20	20	20	20	15	20	18	34	15	24	24	18	16	35	60	115	258
34	20	20	20	20	10	20	15	33	18	23	21	16	17	30	60	110	159
4	20	20	20	19	20	20	18	35	18	24	24	18	17	39	60	119	205
2	20	20	20	19	20	19	18	36	17	24	23	18	18	39	60	118	112
36	20	20	20	18	19	16	17	32	18	23	23	15	17	37	60	113	171
10	20	20	20	17	20	20	18	35	16	24	24	18	17	37	60	117	123
57	20	20	20	10	20	10	15	27	18	19	21	13	14	30	60	100	169
18	20	20	20	9	20	11	16	33	14	18	19	17	16	29	60	100	179
14	20	20	20	9	19	19	15	31	16	22	23	15	16	28	60	107	232
1	20	20	20	9	18	19	16	31	17	21	21	15	16	27	60	106	160
15	20	20	20	6	8	15	14	25	14	15	21	14	11	14	60	89	185
47	20	20	20	2	6	20	13	27	15	17	16	14	13	8	60	88	322
24	20	20	5	18	11	11	12	29	9	18	17	16	13	29	45	85	95
12	20	17	20	20	18	20	17	34	18	22	24	17	17	38	57	115	178
53	20	16	18	20	20	20	18	32	17	23	24	16	16	40	54	114	182
59	20	5	13	18	20	10	15	25	12	16	18	12	13	38	38	86	135
26	20	3	20	4	20	20	13	26	15	17	16	12	14	24	43	87	220
20	20	1	9	20	20	16	13	24	14	18	17	13	11	40	30	86	274
22	20	1	3	18	18	11	11	22	10	13	15	10	12	36	24	71	193
43	19	20	14	20	20	17	17	34	14	23	22	16	18	40	53	110	171
49	17	16	6	20	18	20	13	30	15	19	20	14	16	38	39	97	188
42	16	17	17	6	14	4	10	20	12	15	17	11	9	20	50	74	183
9	15	19	20	18	16	19	18	33	14	22	20	18	15	34	54	107	215
52	14	20	17	20	17	20	15	32	18	24	19	15	17	37	51	108	327
45	14	5	11	20	18	19	15	26	14	14	18	13	13	38	30	87	208
28	11	3	1	19	12	5	6	15	8	12	10	8	7	31	15	51	67
30	9	20	20	20	20	20	17	33	16	22	21	17	16	40	49	109	218
48	8	20	20	20	19	20	16	32	17	22	20	16	16	39	48	107	193
33	4	20	20	20	20	20	15	30	18	21	20	15	15	40	44	104	393
38	3	20	20	20	20	19	16	31	15	20	20	16	15	40	43	102	124
39	3	20	14	14	20	8	13	25	10	17	14	12	13	34	37	79	104
31	1	1	7	11	17	17	9	14	13	10	8	8	6	28	9	54	109
46	4	20	20		2	18	11	20	9	12	12	10	10	2	44	64	328
25	20		1	6	14	11	5	17	13	6	11	7	10	20	21	52	140
8	20		1	4	20	10	9	12	10	11	13	6	6	24	21	55	139
19	1		1	11	8	2	1	4	9	4	5	2	2	19	2	23	143
16		1	1	3	16	8	1	9	6	5	8	4	5	19	2	29	213
44	20			3	20	4	7	12	12	8	8	6	6	23	20	47	163
58			2	7	13	18	5	13	10	9	3	6	7	20	2	40	125
7			1	5	10	6	2	3	7	2	8	1	2	15	1	22	172
17			1	2	8	2	1		7	3	2			10	1	13	81
0		1			1	19	3	7	2	5	4	3	4	1	1	21	88
41			6		20	10	6	9	8	5	8	5	4	20	6	36	96
23				20	1	19	6	12	6	8	8	6	6	21		40	70
3				17	8	12	5	9	6	9	8	5	4	25		37	52
56				7	8	3	2	4	8	4		1	3	15		18	122
29				5	12	7	4	6	6	6	2	3	3	17		24	152
51				1	2	3		2	4			1	1	3		6	82
13					19	5	4	6	5	4	5	2	4	19		24	125
Total	20	20	20	20	20	20	18	36	18	24	24	18	18	40	60		

F1 score: 0.0 – 0.5 – 1.0

Figure S47: Summary of biclusters recovered by BicMix in the Benaroya sorted blood cell dataset after **size factor** normalisation using DESeq's median of ratios method. Each row corresponds to a bicluster and each column corresponds to a sample trait. Each cell shows the number of samples contained in the bicluster which have the trait, and is coloured by the F1 score so that darker colours indicate that the bicluster is specific for that sample trait. *Myeloid* is the sum of the *Monocytes* and *Neutrophils* columns and *Lymphoid* is the sum of *B-Cells*, *CD4* and *CD8* columns. The *Samples* column shows the number of samples in the bicluster and *Genes* shows the number of genes contained in the bicluster.

	Cell type						Disease					MS Treatment		Lineage		Bicluster size	
	B-Cells	CD4	CD8	Monocytes	Neutrophils	Whole blood	ALS	MS	Sepsis	T1D	Healthy	Before IFN-β	After IFN-β	Myeloid	Lymphoid	Samples	Genes
7	19	16	18	14	2	18	13	26	12	18	18	13	13	16	53	87	500
0	18	18	20	14	7	18	15	28	11	19	22	14	14	21	56	95	600
32	18	12	12	18	18	19	17	26	14	21	19	13	13	36	42	97	595
2	17	19	19	19	10	19	16	29	16	20	22	17	12	29	55	103	549
12	16	18	18	20	16	20	17	32	15	20	24	18	14	36	52	108	561
56	16	15	14	15	14	19	13	31	14	20	15	14	17	29	45	93	364
6	14	18	16	19	17	19	17	31	15	21	19	15	16	36	48	103	459
9	11	2	5	20	9	15	9	20	7	12	14	9	11	29	18	62	363
17	5	1	4	20	20	14	10	18	9	15	12	6	12	40	10	64	457
34	2	2	3	19	18	12	7	18	10	9	12	8	10	37	7	56	194
3	1	7	7	17	17	19	10	20	14	14	10	11	9	34	15	68	390
33	20	20	20	14		16	14	28	10	19	19	14	14	14	60	90	402
31	20	20	20	5		17	12	26	10	16	18	14	12	5	60	82	270
58	20	20	19	17		14	14	26	11	20	19	14	12	17	59	90	258
20	20	19	19	16		13	13	27	12	18	17	14	13	16	58	87	309
10	20	14	9	15		19	9	26	13	16	13	11	15	15	43	77	340
21	20	13	14	19		19	15	26	12	16	16	13	13	19	47	85	238
4	19	20	19	19		19	15	30	14	19	18	15	15	19	58	96	297
44	19	19	17	10		13	13	23	8	18	16	13	10	10	55	78	304
19	19	18	19	9		1	11	16	9	16	14	8	8	9	56	66	139
38	19	17	16	18		18	13	30	12	18	15	15	15	18	52	88	388
13	18	20	20	18		17	14	29	13	20	17	15	14	18	58	93	414
5	18	20	20	15		15	13	27	12	18	18	14	13	15	58	88	170
37	18	20	19	9		17	13	27	10	16	17	14	13	9	57	83	212
24	18	20	18	7		12	12	22	7	18	16	12	10	7	56	75	251
28	18	18	19	20		19	15	27	14	18	20	12	15	20	55	94	293
49	17	20	19	19		11	15	25	12	19	15	13	12	19	56	86	161
57	17	19	20	20		17	13	29	13	19	19	15	14	20	56	93	276
43	17	19	20	7		14	13	25	9	14	16	12	13	7	56	77	584
14	17	19	16	17		19	14	25	12	18	19	13	12	17	52	88	327
46		5	4	13	2	20	7	15	9	7	6	7	8	15	9	44	184
1	19	20	20			5	10	20	8	12	14	11	9		59	64	243
22	18	20	20			4	10	18	9	13	12	10	8		58	62	225
47	18	18	20			16	11	22	8	15	16	12	10		56	72	263
51	14	20	20			6	8	17	9	14	12	9	8		54	60	321
59	2	20	20			6	9	13	6	10	10	7	6		42	48	306
45	8		1	20		13	6	15	6	7	8	7	8	20	9	42	145
52			1	19	20	19	9	16	10	12	12	8	8	39	1	59	356
29			1	18	18	19	7	18	10	11	10	9	9	36	1	56	291
36			1	1	19	5	3	6	7	4	6	3	3	20	1	26	433
50	1			20		18	6	11	6	8	8	5	6	20	1	39	203
15				20	20	12	8	13	8	11	12	7	6	40		52	319
53				20	19	19	8	17	9	12	12	9	8	39		58	189
55				20	12	7	7	11	6	7	8	6	5	32		39	158
30				18	18	12	8	14	6	10	10	6	8	36		48	486
8				17	20	13	6	17	8	11	8	8	9	37		50	401
16				17	17	17	7	15	8	12	9	7	8	34		51	192
41				12	20	14	7	15	7	8	9	6	9	32		46	366
40				3	17	5	3	5	9	4	4	4	1	20		25	247
54				2	14	3	3	9		3	4	1	8	16		19	318
25				2	3	11	1	4	8	2	1	2	2	5		16	136
48				20		8	4	10	4	5	5	5	5	20		28	156
11					20	14	4	9	6	7	8	5	4	20		34	477
35					20	4	3	6	6	4	5	3	3	20		24	328
42					20	4	3	7	4	4	6	3	4	20		24	444
26					19	5	3	8	6	4	3	3	5	19		24	412
18					17	13	4	7	6	6	7	4	3	17		30	503
39	20						3	6	3	4	4	3	3		20	20	222
27				20			3	6	3	4	4	3	3	20		20	196
23					19		3	6	2	4	4	3	3	19		19	285
Total	20	20	20	20	20	20	18	36	18	24	24	18	18	40	60		

0.0 0.5 1.0
F1 score

Figure S48: Summary of biclusters recovered by nsNMF in the Benaroya sorted blood cell dataset after **size factor** normalisation using DESeq's median of ratios method. Each row corresponds to a bicluster and each column corresponds to a sample trait. Each cell shows the number of samples contained in the bicluster which have the trait, and is coloured by the F1 score so that darker colours indicate that the bicluster is specific for that sample trait. *Myeloid* is the sum of the *Monocytes* and *Neutrophils* columns and *Lymphoid* is the sum of *B-Cells*, *CD4* and *CD8* columns. The *Samples* column shows the number of samples in the bicluster and *Genes* shows the number of genes contained in the bicluster.

	Cell type						Disease					MS Treatment		Lineage		Bicluster size	
	B-Cells	CD4	CD8	Monocytes	Neutrophils	Whole blood	ALS	MS	Sepsis	T1D	Healthy	Before IFN-β	After IFN-β	Myeloid	Lymphoid	Samples	Genes
0	20	20	20	20	20	20	18	36	18	24	24	18	18	40	60	120	576
53	19	15	1	17	6	6	11	21	9	10	13	11	10	23	35	64	492
20	17	17	14	6	10	14	11	21	11	18	17	11	10	16	48	78	491
55	17	5	18	2	5	5	8	15	10	10	9	7	8	7	40	52	428
40	14	20	8	8	8	2	9	19	5	12	15	9	10	16	42	60	505
46	12	10	14	8	10	12	8	23	8	14	13	11	12	18	36	66	375
39	12	4	13	18	15	2	11	17	8	14	14	10	7	33	29	64	307
28	11	10	13	8	13	7	17	18	11	2	14	10	8	21	34	62	367
23	11	8	1	8	11	14	9	17	6	10	11	9	8	19	20	53	401
33	11	5	2	10	6	2	3	8	5	10	10	5	3	16	18	36	475
52	10	17	12	5	5	12	7	19	10	11	14	9	10	10	39	61	378
43	10	10	1	19	3	5	10	13	8	7	10	8	5	22	21	48	372
7	9	5	8	13	13	11	11	18	6	16	8	9	9	26	22	59	461
13	9	4	4	10	2	12	5	9	6	14	7	5	4	12	17	41	332
24	8	14	15	6	10	11	14	17	11	11	11	8	9	16	37	64	394
17	8	6	8	6	4	14	7	16	5	8	10	8	8	10	22	46	231
34	7	16	16	6	9	14	10	19	9	18	12	8	11	15	39	68	300
41	7	14	1	6	7	6	7	12	7	9	6	6	6	13	22	41	314
54	6	16	2	12	6	6	6	13	9	12	8	7	6	18	24	48	374
35	6	10	11	8	10	5	8	16	5	7	14	8	8	18	27	50	340
21	6	8	7	7	18	7	6	14	7	15	11	7	7	25	21	53	276
45	6	8	5	7	9	13	7	12	10	11	8	8	4	16	19	48	499
57	5	12	11	5	8	2	7	10	9	8	9	6	4	13	28	43	457
9	5	10	11	2	4	3	7	9	5	7	7	4	5	6	26	35	315
49	5	8	1	11	12	2	4	14	5	4	12	5	9	23	14	39	467
31	5	3	11	8	3	14	6	13	8	7	10	6	7	11	19	44	293
36	5	2	9	4	12	3	7	9	8	4	7	5	4	16	16	35	430
37	4	6	4	9	10	6	5	11	9	4	10	5	6	19	14	39	477
19	4	5	3	5	7	10	3	10	7	8	6	6	4	12	12	34	317
10	2	20	20	8	16	12	12	24	10	18	14	11	13	24	42	78	518
25	2	15	14	6	6	6	8	14	6	10	11	6	8	12	31	49	425
48	2	13	19	6	4	13	12	19	8	6	12	8	11	10	34	57	447
16	2	7	14	5	6	6	5	10	8	10	7	5	5	11	23	40	529
58	2	4	6	14	7	10	7	15	10	2	9	8	7	21	12	43	401
42	2	2	9	4	11	7	6	13	2	8	6	7	6	15	13	35	529
32	13	7	8	8	10		9	15	10	2	10	7	8	18	28	46	261
15	1	4	8	14	12		5	10	7	10	7	5	5	26	13	39	500
1	20	20	20	18		12	14	27	12	20	17	14	13	18	60	90	387
29	10	3	2	8		6	5	9	7	2	6	6	3	8	15	29	364
59	4	7	8	14		9	7	13	8	8	6	8	5	14	19	42	477
18	3	2	1		7	2	2	4	1	5	3	2	2	7	6	15	307
12	18	2		8	16	6	5	15	9	10	11	8	7	24	20	50	409
47	12	12		8	14	12	10	14	13	9	12	7	7	22	24	58	636
38	11	7		10	8	9	4	13	6	12	10	5	8	18	18	45	521
44	8	16		2	10	12	8	13	9	8	10	6	7	12	24	48	404
51	6	5		6	5	9	6	6	4	9	6	4	2	11	11	31	347
6	2	2		3	10	13	2	7	10	6	5	3	4	13	4	30	331
27	6		18	12	6	12	3	16	9	18	8	8	8	18	24	54	417
50	3		15	17	11	7	11	16	7	11	8	10	6	28	18	53	417
8		16	8	18	16	20	11	21	12	20	14	11	10	34	24	78	430
56		5	8	7	9	17	5	15	9	8	9	7	8	16	13	46	486
4	2	3		5	4		2	5	1	2	4	1	4	9	5	14	421
14	2	1		2	8		1	5	1	3	3	2	3	10	3	13	355
22	10			18	6	12	6	14	6	12	8	7	7	24	10	46	520
30	5			7	8	2	2	6	2	7	5	4	2	15	5	22	351
11	3			3	6	3	3	3	4	3	2	1	2	9	3	15	257
3	1			2	20	2	5	7	3	4	6	3	4	22	1	25	433
26			10	13	13	10	7	17	5	7	10	8	9	26	10	46	431
2			2	1	8	9	1	7	6	3	3	3	4	9	2	20	199
5	6			20	4		3	10	4	6	7	5	5	24	6	30	439
Total	20	20	20	20	20	20	18	36	18	24	24	18	18	40	60		

0.0 0.5 1.0
F1 score

Figure S49: Summary of biclusters recovered by MultiCluster in the Benaroya sorted blood cell dataset after **size factor** normalisation using DESeq's median of ratios method. Each row corresponds to a bicluster and each column corresponds to a sample trait. Each cell shows the number of samples contained in the bicluster which have the trait, and is coloured by the F1 score so that darker colours indicate that the bicluster is specific for that sample trait. *Myeloid* is the sum of the *Monocytes* and *Neutrophils* columns and *Lymphoid* is the sum of *B-Cells*, *CD4* and *CD8* columns. The *Samples* column shows the number of samples in the bicluster and *Genes* shows the number of genes contained in the bicluster.

	Cell type						Disease					MS Treatment		Lineage		Bicluster size	
	B-Cells	CD4	CD8	Monocytes	Neutrophils	Whole blood	ALS	MS	Sepsis	T1D	Healthy	Before IFN-β	After IFN-β	Myeloid	Lymphoid	Samples	Genes
19	20	20	20	20	20	20	18	36	18	24	24	18	18	40	60	120	3437
23	20	20	20	20	20	16	18	34	18	23	23	17	17	40	60	116	4191
18	20	20	20	16	19	16	17	31	18	23	22	15	16	35	60	111	83
35	20	20	19	18	19	17	18	33	17	22	23	16	17	37	59	113	68
32	20	20	17	19	20	17	18	34	16	23	22	17	17	39	57	113	148
7	20	19	19	17	19	18	14	34	16	24	24	17	17	36	58	112	81
31	20	19	18	16	19	18	14	33	16	24	23	16	17	35	57	110	139
22	20	19	18	16	18	18	17	34	16	22	20	16	18	34	57	109	44
3	20	18	19	19	17	17	16	36	18	18	22	18	18	36	57	110	96
13	20	18	18	19	17	17	17	34	16	20	22	17	17	36	56	109	70
26	20	16	15	20	20	20	16	33	17	22	23	18	15	40	51	111	3718
6	19	20	19	18	17	20	16	34	17	23	23	17	17	35	58	113	3000
2	19	19	18	14	14	20	16	31	17	21	19	15	16	28	56	104	3215
8	19	18	19	19	20	16	14	36	18	21	22	18	18	39	56	111	16
21	19	16	20	18	16	18	16	32	17	23	19	16	16	34	55	107	2988
29	18	19	20	16	19	19	14	36	17	22	22	18	18	35	57	111	34
17	18	18	16	18	18	18	16	32	18	23	17	16	16	36	52	106	131
10	18	17	19	16	19	16	17	30	18	20	20	12	18	35	54	105	64
30	18	17	17	18	20	17	16	35	16	20	20	18	17	38	52	107	8
0	17	19	19	19	18	17	18	33	16	20	22	18	15	37	55	109	17
1	17	19	19	19	15	20	17	32	16	20	24	15	17	34	55	109	8
28	17	19	19	16	16	19	18	31	18	19	20	13	18	32	55	106	79
4	17	18	17	17	17	18	16	32	17	20	19	16	16	34	52	104	56
24	16	20	19	16	18	18	17	31	16	20	23	16	15	34	55	107	28
5	16	19	18	18	17	20	18	31	16	20	23	17	14	35	53	108	24
33	16	19	18	15	19	18	16	29	16	21	23	16	13	34	53	105	56
36	16	17	19	19	20	19	18	34	18	18	22	16	18	39	52	110	41
25	15	16	11	20	10	20	12	29	16	17	18	14	15	30	42	92	3204
16	14	19	18	9	7	15	12	26	12	16	16	12	14	16	51	82	3037
15	12	12	11	12	17	12	7	24	14	19	12	15	9	29	35	76	3006
9	10	11	7	16	15	17	12	26	15	6	17	11	15	31	28	76	2901
11	6	10	7	8	6	12	9	15	10	7	8	6	9	14	23	49	166
34	1	2	1	7	5	4	1	14	2	1	2	2	12	12	4	20	36
27	1	2	2	6		7	3	6	2	3	4	2	4	6	5	18	37
20	2	1	1		4	1	4	2		3		1	1	4	4	9	3
14			1	6	9	6		10	9		3	1	9	15	1	22	362
12					4		1	1	1	1			1	4		4	15
Total	20	20	20	20	20	20	18	36	18	24	24	18	18	40	60		

0.0 0.5 1.0
F1 score

Figure S50: Summary of biclusters recovered by BicMix-Q in the Benaroya sorted blood cell dataset after **size factor** normalisation using DESeq's median of ratios method. Each row corresponds to a bicluster and each column corresponds to a sample trait. Each cell shows the number of samples contained in the bicluster which have the trait, and is coloured by the F1 score so that darker colours indicate that the bicluster is specific for that sample trait. *Myeloid* is the sum of the *Monocytes* and *Neutrophils* columns and *Lymphoid* is the sum of *B-Cells*, *CD4* and *CD8* columns. The *Samples* column shows the number of samples in the bicluster and *Genes* shows the number of genes contained in the bicluster.

	Cell type						Disease					MS Treatment		Lineage		Bicluster size	
	B-Cells	CD4	CD8	Monocytes	Neutrophils	Whole blood	ALS	MS	Sepsis	T1D	Healthy	Before IFN-β	After IFN-β	Myeloid	Lymphoid	Samples	Genes
1	20	20	20	20	20	20	18	36	18	24	24	18	18	40	60	120	566
2	20	20	20	20	20	20	18	36	18	24	24	18	18	40	60	120	1575
12	20	20	20	20	20	20	18	36	18	24	24	18	18	40	60	120	270
15	20	20	20	20	20	20	18	36	18	24	24	18	18	40	60	120	302
17	20	20	20	20	20	20	18	36	18	24	24	18	18	40	60	120	1572
34	20	20	20	20	20	20	18	36	18	24	24	18	18	40	60	120	990
48	20	20	20	20	20	20	18	36	18	24	24	18	18	40	60	120	364
11	20	20	20	20	20	14	18	34	15	24	23	17	17	40	60	114	575
13	20	20	20	20	20	14	18	34	18	20	24	17	17	40	60	114	945
47	20	20	20	20	20	8	15	32	15	24	22	16	16	40	60	108	793
35	20	20	20	20	16	18	16	34	18	24	22	17	17	36	60	114	1888
23	20	20	20	20	14	20	17	34	16	24	23	17	17	34	60	114	601
25	20	20	20	20	14	14	14	32	16	24	22	16	16	34	60	108	2249
58	20	20	20	20	14	14	15	34	17	20	22	17	17	34	60	108	1295
0	20	20	20	20	10	18	15	32	16	24	21	16	16	30	60	108	599
42	20	20	20	18	10	20	16	31	16	24	21	16	15	28	60	108	1380
19	20	20	20	14	20	20	16	35	16	24	23	17	18	34	60	114	1343
27	20	20	20	14	20	20	18	34	17	22	23	17	17	34	60	114	206
10	20	20	20	14	20	14	16	33	16	20	23	16	17	34	60	108	1407
31	20	20	20	14	20	14	16	33	13	24	22	16	17	34	60	108	259
29	20	20	20	12	16	20	17	31	18	22	20	16	15	28	60	108	771
8	20	20	14	20	20	20	15	34	18	24	23	17	17	40	54	114	293
43	20	20	14	20	20	20	15	34	18	24	23	17	17	40	54	114	546
46	20	20	14	20	20	20	18	34	18	20	24	17	17	40	54	114	1944
51	20	20	14	20	20	14	18	32	18	16	24	16	16	40	54	108	1285
20	20	20	14	14	14	20	15	31	14	20	22	15	16	28	54	102	353
57	20	20	8	20	16	18	16	30	15	20	21	15	15	36	48	102	247
49	20	16	18	20	20	20	16	34	18	24	22	17	17	40	54	114	1245
5	20	16	18	20	20	14	16	32	15	24	21	16	16	40	54	108	788
39	20	16	18	14	20	20	16	32	18	22	20	16	16	34	54	108	860
26	20	16	12	20	20	20	13	32	18	24	21	16	16	40	48	108	471
41	20	14	20	20	16	18	16	34	17	20	21	17	17	36	54	108	2229
33	20	14	20	20	14	14	18	34	16	12	22	17	17	34	54	102	1706
53	20	12	12	20	6	8	8	24	11	20	15	13	11	26	44	78	1272
9	20	4	18	2	8	20	12	25	9	12	14	12	13	10	42	72	642
30	18	16	20	14	20	20	17	31	18	22	20	16	15	34	54	108	1725
59	18	10	20	20	14	20	16	31	15	20	20	16	15	34	48	102	1838
38	15	18	18	13	10	17	16	29	13	15	18	16	13	23	51	91	1392
4	14	20	20	20	20	20	16	35	16	24	23	17	18	40	54	114	674
32	14	20	20	20	20	20	18	34	18	22	22	17	17	40	54	114	1190
44	14	20	20	20	20	20	18	34	18	22	22	17	17	40	54	114	784
16	14	20	20	20	14	20	15	33	14	24	22	16	17	34	54	108	566
55	14	20	20	14	16	18	16	30	16	20	20	15	15	30	54	102	470
54	14	20	20	12	16	14	17	27	14	20	18	14	13	28	54	96	1851
18	14	16	18	14	20	20	16	30	18	20	18	15	15	34	48	102	1429
24	14	10	12	20	20	20	14	31	15	16	20	15	16	40	36	96	720
14	14	8	20	4	8	20	13	24	10	12	15	11	13	12	42	74	1001
50	12	16	20	12	16	20	14	27	16	22	17	14	13	28	48	96	2119
36	12	16	14	20	20	20	17	29	15	22	19	15	14	40	42	102	1118
56	11	8	14	8	14	15	10	23	13	10	14	11	12	22	33	70	930
40	8	20	14	20	20	20	13	31	16	22	20	15	16	40	42	102	1302
7	7	20	18	12	8	20	14	26	12	16	17	12	14	20	45	85	1022
28	2	10	12		4	14	4	15	8	8	7	7	8	4	24	42	1589
45	1		2	9	13	5	6	9	6	4	5	4	5	22	3	30	1175
3			4	6	8	14	5	11	5	6	5	6	5	14	4	32	1027
6			2	14	10	8	5	13	6	2	8	6	7	24	2	34	1065
22			2	3	5	10	3	7	5	2	3	3	4	8	2	20	781
52				8	20	14	9	12	8	4	9	6	6	28		42	1026
37				2	14	11	5	11	5		6	5	6	16		27	1015
21			1			5		1	4		1		1		1	6	1095
Total	20	20	20	20	20	20	18	36	18	24	24	18	18	40	60		

0.0 0.5 1.0
F1 score

Figure S51: Summary of biclusters recovered by SDA in the Benaroya sorted blood cell dataset after **size factor** normalisation using DESeq's median of ratios method. Each row corresponds to a bicluster and each column corresponds to a sample trait. Each cell shows the number of samples contained in the bicluster which have the trait, and is coloured by the F1 score so that darker colours indicate that the bicluster is specific for that sample trait. *Myeloid* is the sum of the *Monocytes* and *Neutrophils* columns and *Lymphoid* is the sum of *B-Cells*, *CD4* and *CD8* columns. The *Samples* column shows the number of samples in the bicluster and *Genes* shows the number of genes contained in the bicluster.

	Cell type						Disease					MS Treatment		Lineage		Bicluster size	
	B-Cells	CD4	CD8	Monocytes	Neutrophils	Whole blood	ALS	MS	Sepsis	T1D	Healthy	Before IFN-β	After IFN-β	Myeloid	Lymphoid	Samples	Genes
0	20	20	20	20	20	20	18	36	18	24	24	18	18	40	60	120	230
1	20	20	20	20	20	20	18	36	18	24	24	18	18	40	60	120	307
2	20	20	20	20	20	20	18	36	18	24	24	18	18	40	60	120	272
5	20	20	20	20	20	20	18	36	18	24	24	18	18	40	60	120	258
6	20	20	20	20	20	20	18	36	18	24	24	18	18	40	60	120	162
8	20	20	20	20	20	20	18	36	18	24	24	18	18	40	60	120	323
9	20	20	20	20	20	20	18	36	18	24	24	18	18	40	60	120	496
10	20	20	20	20	20	20	18	36	18	24	24	18	18	40	60	120	341
11	20	20	20	20	20	20	18	36	18	24	24	18	18	40	60	120	294
12	20	20	20	20	20	20	18	36	18	24	24	18	18	40	60	120	259
14	20	20	20	20	20	20	18	36	18	24	24	18	18	40	60	120	323
15	20	20	20	20	20	20	18	36	18	24	24	18	18	40	60	120	244
16	20	20	20	20	20	20	18	36	18	24	24	18	18	40	60	120	233
18	20	20	20	20	20	20	18	36	18	24	24	18	18	40	60	120	378
19	20	20	20	20	20	20	18	36	18	24	24	18	18	40	60	120	516
20	20	20	20	20	20	20	18	36	18	24	24	18	18	40	60	120	366
23	20	20	20	20	20	20	18	36	18	24	24	18	18	40	60	120	407
24	20	20	20	20	20	20	18	36	18	24	24	18	18	40	60	120	500
25	20	20	20	20	20	20	18	36	18	24	24	18	18	40	60	120	357
26	20	20	20	20	20	20	18	36	18	24	24	18	18	40	60	120	551
27	20	20	20	20	20	20	18	36	18	24	24	18	18	40	60	120	524
29	20	20	20	20	20	20	18	36	18	24	24	18	18	40	60	120	401
30	20	20	20	20	20	20	18	36	18	24	24	18	18	40	60	120	241
31	20	20	20	20	20	20	18	36	18	24	24	18	18	40	60	120	357
36	20	20	20	20	20	20	18	36	18	24	24	18	18	40	60	120	312
37	20	20	20	20	20	20	18	36	18	24	24	18	18	40	60	120	219
38	20	20	20	20	20	20	18	36	18	24	24	18	18	40	60	120	221
39	20	20	20	20	20	20	18	36	18	24	24	18	18	40	60	120	261
41	20	20	20	20	20	20	18	36	18	24	24	18	18	40	60	120	482
43	20	20	20	20	20	20	18	36	18	24	24	18	18	40	60	120	122
47	20	20	20	20	20	20	18	36	18	24	24	18	18	40	60	120	265
48	20	20	20	20	20	20	18	36	18	24	24	18	18	40	60	120	199
49	20	20	20	20	20	20	18	36	18	24	24	18	18	40	60	120	494
54	20	20	20	20	20	20	18	36	18	24	24	18	18	40	60	120	330
55	20	20	20	20	20	20	18	36	18	24	24	18	18	40	60	120	425
56	20	20	20	20	20	20	18	36	18	24	24	18	18	40	60	120	240
59	20	20	20	20	20	20	18	36	18	24	24	18	18	40	60	120	435
52	20	20	20	20	17	20	17	35	17	24	24	17	18	37	60	117	102
50	20	20	20	20	11	20	17	32	16	23	23	16	16	31	60	111	473
45	20	20	20	20	8	20	18	31	17	21	21	16	15	28	60	108	181
28	20	20	20	20	7	20	16	33	17	20	21	17	16	27	60	107	167
33	20	20	20	20	7	20	15	31	17	21	23	16	15	27	60	107	147
57	20	20	20	20	7	20	15	32	17	21	22	17	15	27	60	107	198
3	20	20	20	20	6	20	16	31	15	22	22	15	16	26	60	106	207
51	20	20	20	20	5	20	15	32	16	20	22	16	16	25	60	105	211
21	20	20	20	20	3	20	15	30	17	20	21	15	15	23	60	103	106
22	20	20	20	20	3	20	15	30	17	20	21	15	15	23	60	103	209
40	20	20	20	20	3	20	15	30	17	20	21	15	15	23	60	103	45
46	20	20	20	20	3	20	15	30	17	20	21	15	15	23	60	103	133
53	20	20	20	20	3	20	15	30	17	20	21	15	15	23	60	103	201
4	20	20	20	20	1	20	15	30	15	20	21	15	15	21	60	101	120
32	20	20	20	20	1	20	15	30	15	20	21	15	15	21	60	101	180
7	19	20	20	20	20	20	18	36	17	24	24	18	18	40	59	119	401
42	16	20	20	20	20	20	17	34	18	23	24	17	17	40	56	116	335
17	11	4	8	20	20	20	12	23	14	15	19	10	13	40	23	83	236
58	1	1	1	20	20	20	9	18	12	12	12	9	9	40	3	63	190
13	20	20	20	20		20	15	30	15	20	20	15	15	20	60	100	208
34			1	20	20	20	9	18	10	12	12	9	9	40	1	61	213
35			1	20	20	20	9	18	10	12	12	9	9	40	1	61	211
44			1	20	20	20	9	18	10	12	12	9	9	40	1	61	155
Total	20	20	20	20	20	20	18	36	18	24	24	18	18	40	60		

0.0 0.5 1.0

F1 score

Figure S52: Summary of biclusters recovered by SNMF in the Benaroya sorted blood cell dataset after **size factor** normalisation using DESeq's median of ratios method. Each row corresponds to a bicluster and each column corresponds to a sample trait. Each cell shows the number of samples contained in the bicluster which have the trait, and is coloured by the F1 score so that darker colours indicate that the bicluster is specific for that sample trait. *Myeloid* is the sum of the *Monocytes* and *Neutrophils* columns and *Lymphoid* is the sum of *B-Cells*, *CD4* and *CD8* columns. The *Samples* column shows the number of samples in the bicluster and *Genes* shows the number of genes contained in the bicluster.

	Cell type						Disease					MS Treatment		Lineage		Bicluster size	
	B-Cells	CD4	CD8	Monocytes	Neutrophils	Whole blood	ALS	MS	Sepsis	T1D	Healthy	Before IFN-β	After IFN-β	Myeloid	Lymphoid	Samples	Genes
11	20	19	18	19	19	17	16	33	17	24	22	16	17	38	57	112	3261
56	20	19	18	18	18	17	17	32	16	23	22	14	18	36	57	110	2974
3	20	19	17	17	19	19	17	34	17	21	22	17	17	36	56	111	2940
29	20	19	16	19	20	19	16	33	18	23	23	17	16	39	55	113	3428
37	20	18	18	18	18	20	16	32	18	23	23	15	17	36	56	112	3323
40	20	18	18	18	18	18	16	34	14	23	23	16	18	36	56	110	3078
16	20	18	18	16	18	20	17	33	16	22	22	17	16	34	56	110	2864
36	20	17	19	18	19	18	17	32	16	22	24	14	18	37	56	111	3146
50	20	17	18	18	19	18	16	33	17	21	23	17	16	37	55	110	3119
25	20	17	18	18	18	18	16	33	17	23	20	16	17	36	55	109	2885
38	20	17	17	18	18	18	12	33	17	23	23	16	17	36	54	108	3231
58	20	17	14	19	20	14	14	28	18	21	23	15	13	39	51	104	3361
41	19	20	19	19	18	16	16	32	18	21	24	16	16	37	58	111	3017
5	19	20	18	18	18	19	17	34	17	23	21	16	18	36	57	112	3252
15	19	19	19	19	19	17	17	32	18	22	23	18	14	38	57	112	3320
52	19	19	19	18	17	19	17	33	17	21	23	16	17	35	57	111	3098
33	19	19	18	19	18	20	17	34	17	23	22	17	17	37	56	113	3167
2	19	19	17	19	20	19	18	33	17	23	22	18	15	39	55	113	3003
0	19	19	16	18	17	18	15	32	17	22	21	17	15	35	54	107	3286
31	19	19	15	19	18	15	15	32	16	20	22	15	17	37	53	105	2988
24	19	17	19	19	19	18	17	31	17	24	22	16	15	38	55	111	3135
26	19	17	19	19	18	18	17	33	16	21	23	17	16	37	55	110	3314
21	19	17	17	18	20	18	17	33	18	19	22	17	16	38	53	109	3251
10	19	17	16	17	19	16	16	31	17	18	22	16	15	36	52	104	3221
46	19	16	18	16	20	17	14	32	17	22	21	15	17	36	53	106	3243
19	19	15	18	17	20	18	18	31	16	23	19	17	14	37	52	107	3177
34	18	20	18	20	20	17	15	35	17	23	23	18	17	40	56	113	3299
51	18	20	16	20	18	17	17	29	18	22	23	14	15	38	54	109	3256
7	18	19	19	20	18	17	17	33	17	23	21	17	16	38	56	111	2783
6	18	19	19	19	19	15	17	33	17	20	22	17	16	38	56	109	3308
43	18	19	18	18	19	19	18	34	17	20	22	17	17	37	55	111	3250
4	18	19	17	20	20	19	15	32	18	24	24	17	15	40	54	113	3252
44	18	18	19	19	19	17	17	35	15	20	23	17	18	38	55	110	3110
49	18	18	19	18	20	18	17	33	17	22	22	15	18	38	55	111	3158
42	18	17	18	18	19	18	18	31	18	21	20	15	16	37	53	108	3156
45	18	17	16	18	17	19	17	29	18	20	21	12	17	35	51	105	3135
53	18	17	15	19	18	18	17	32	15	22	19	16	16	37	50	105	3279
54	18	16	18	19	20	19	16	34	16	23	21	16	18	39	52	110	3173
57	18	16	18	19	18	18	16	33	16	21	21	15	18	37	52	107	2828
48	17	20	20	19	19	19	15	34	18	24	23	18	16	38	57	114	2935
18	17	20	18	19	20	19	16	35	18	23	21	17	18	39	55	113	3338
32	17	19	20	17	20	19	16	32	18	22	24	15	17	37	56	112	3133
27	17	19	18	19	19	18	16	34	16	22	22	18	16	38	54	110	3102
12	17	19	18	18	19	15	15	34	15	23	19	17	17	37	54	106	3277
47	17	18	18	19	20	18	18	32	17	20	23	17	15	39	53	110	3335
13	17	18	17	19	19	16	15	34	15	23	19	17	17	38	52	106	3083
39	17	18	17	18	19	19	18	32	17	20	21	16	16	37	52	108	3214
23	17	17	18	16	19	17	13	34	16	22	19	16	18	35	52	104	3378
1	17	17	17	18	19	20	17	34	16	17	24	17	17	37	51	108	3026
17	17	17	15	20	18	18	13	33	16	23	20	17	16	38	49	105	3075
20	17	16	16	20	20	19	13	32	18	22	23	16	16	40	49	108	3112
28	17	15	20	18	17	16	15	30	17	21	20	15	15	35	52	103	3037
55	16	19	18	20	20	19	16	34	17	21	24	17	17	40	53	112	3234
59	16	18	18	19	19	18	14	33	17	21	23	17	16	38	52	108	3056
14	16	17	17	18	20	19	17	34	16	20	20	17	17	38	50	107	3301
8	16	16	19	19	19	18	17	31	15	23	21	15	16	38	51	107	3326
35	16	16	18	19	19	19	14	34	16	20	23	16	18	38	50	107	3228
9	15	19	18	19	18	18	16	32	16	21	22	15	17	37	52	107	3220
22	15	18	20	20	19	20	18	32	17	22	23	15	17	39	53	112	3166
30	15	18	18	17	19	16	15	27	17	23	21	12	15	36	51	103	3019
Total	20	20	20	20	20	20	18	36	18	24	24	18	18	40	60		

0.0 0.5 1.0
F1 score

Figure S53: Summary of biclusters recovered by FABIA in the Benaroya sorted blood cell dataset after **size factor** normalisation using DESeq's median of ratios method. Each row corresponds to a bicluster and each column corresponds to a sample trait. Each cell shows the number of samples contained in the bicluster which have the trait, and is coloured by the F1 score so that darker colours indicate that the bicluster is specific for that sample trait. *Myeloid* is the sum of the *Monocytes* and *Neutrophils* columns and *Lymphoid* is the sum of *B-Cells*, *CD4* and *CD8* columns. The *Samples* column shows the number of samples in the bicluster and *Genes* shows the number of genes contained in the bicluster.

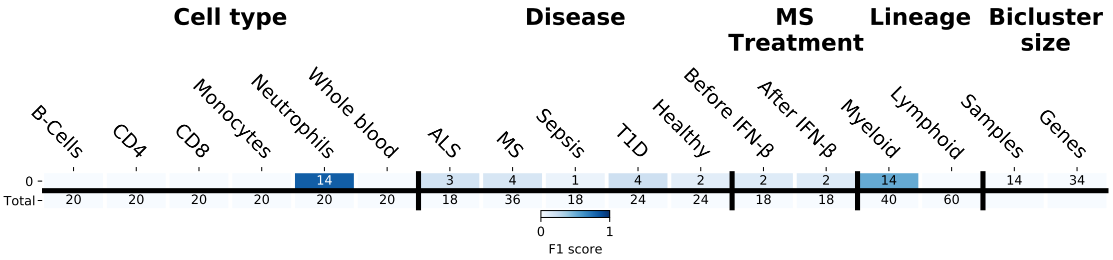

Figure S54: Summary of biclusters recovered by Plaid in the Benaroya sorted blood cell dataset after **size factor** normalisation using DESeq's median of ratios method. Each row corresponds to a bicluster and each column corresponds to a sample trait. Each cell shows the number of samples contained in the bicluster which have the trait, and is coloured by the F1 score so that darker colours indicate that the bicluster is specific for that sample trait. *Myeloid* is the sum of the *Monocytes* and *Neutrophils* columns and *Lymphoid* is the sum of *B-Cells*, *CD4* and *CD8* columns. The *Samples* column shows the number of samples in the bicluster and *Genes* shows the number of genes contained in the bicluster.

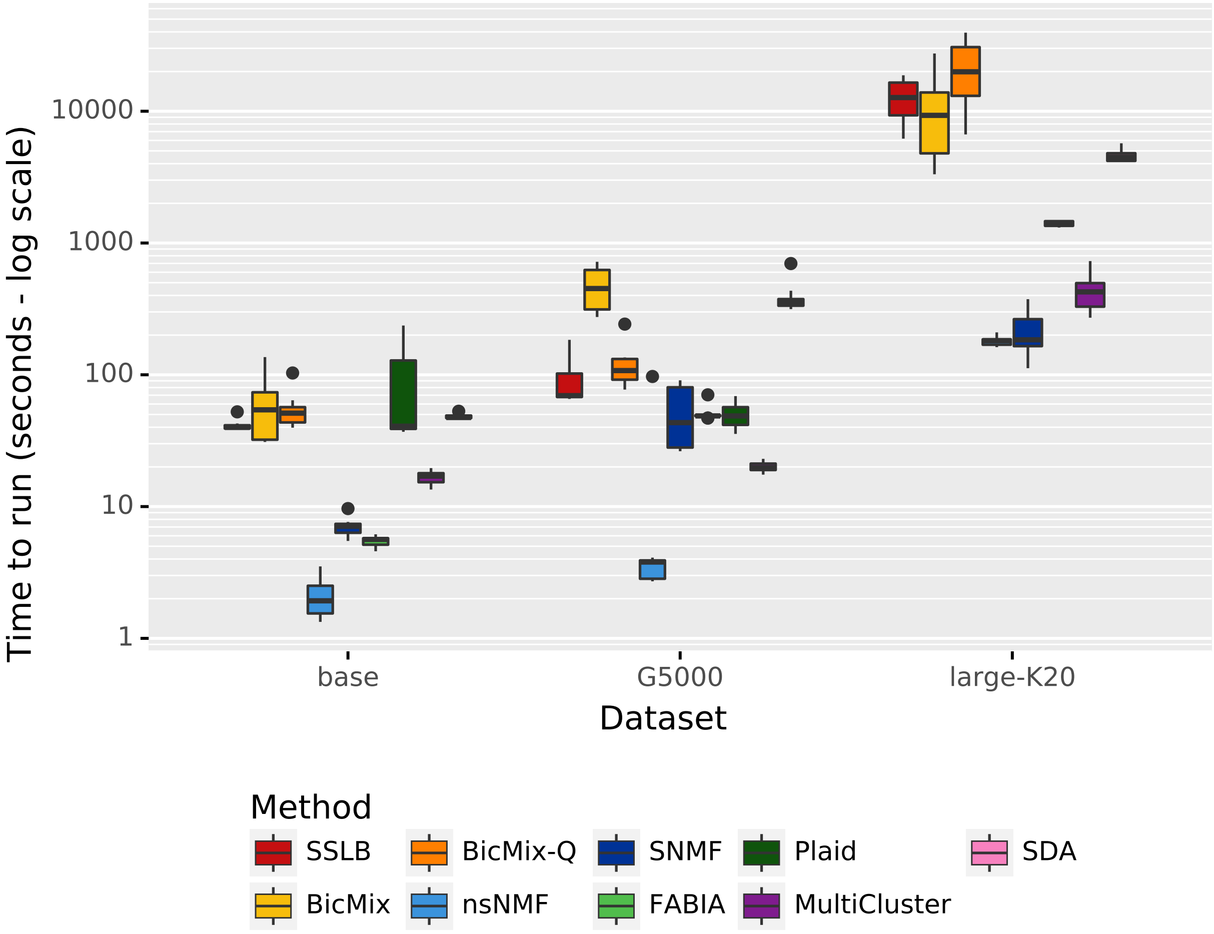

Figure S55: Mean run time across large datasets (log scale). All algorithms were run with $K_{init} = 20$, except for *Adaptive* algorithms which used $K_{init} = 25$. The *base* dataset has 1000 genes and 100 samples, the *G5000* dataset has 5000 genes and 100 samples and the *large-K20* dataset has 10000 genes and 6000 samples. All have 20 latent biclusters. Note that Plaid failed to run on the *large-K20* dataset due to memory constraints.

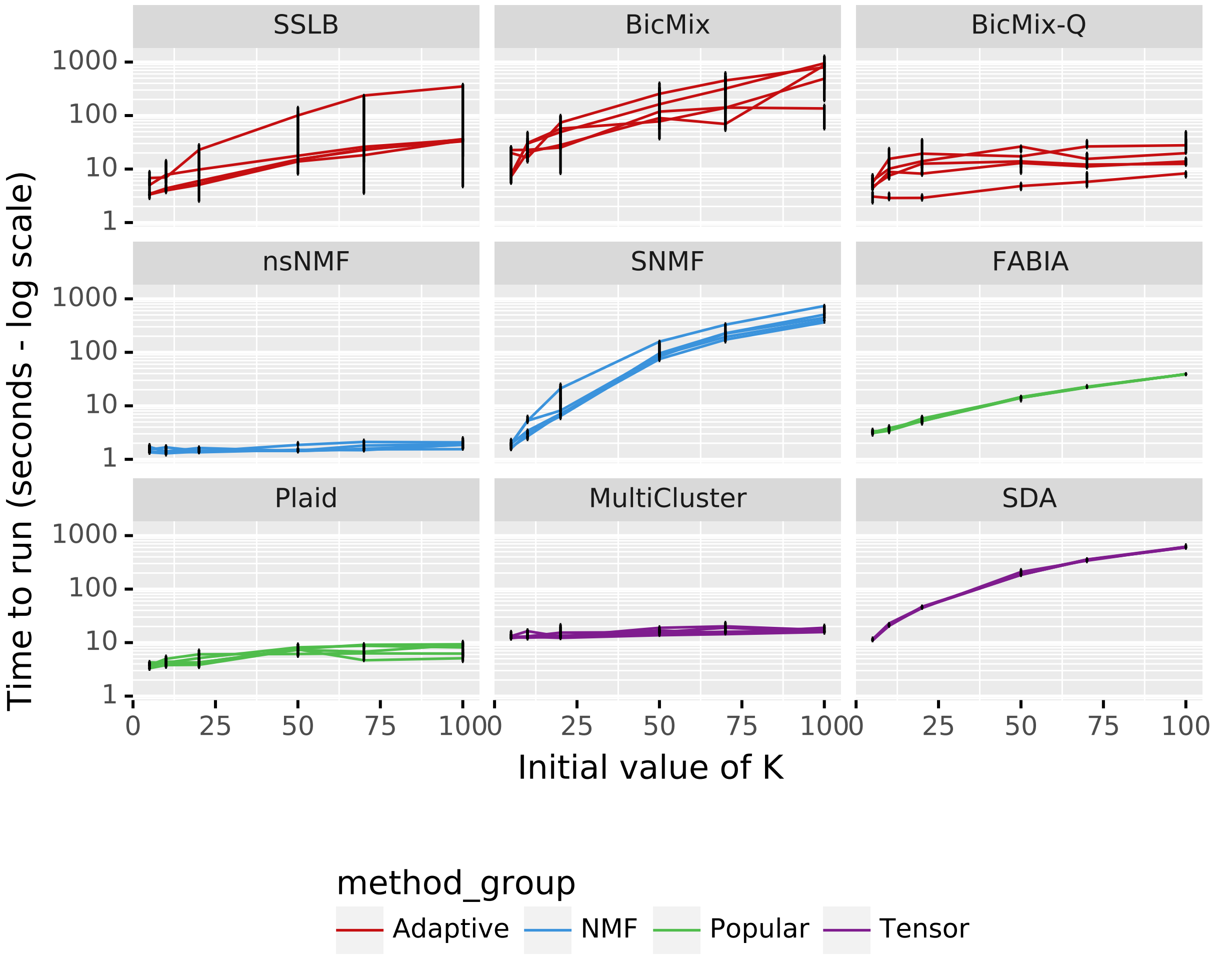

Figure S56: Effect of K_{init} on run time. Each line shows median run time (log scale) for a different dataset type, which differ only in number of latent biclusters (*K5*, *K10*, *base*, *K50* and *K70*). The flatter the line, the less sensitive the runtime is to the choice of K_{init}.

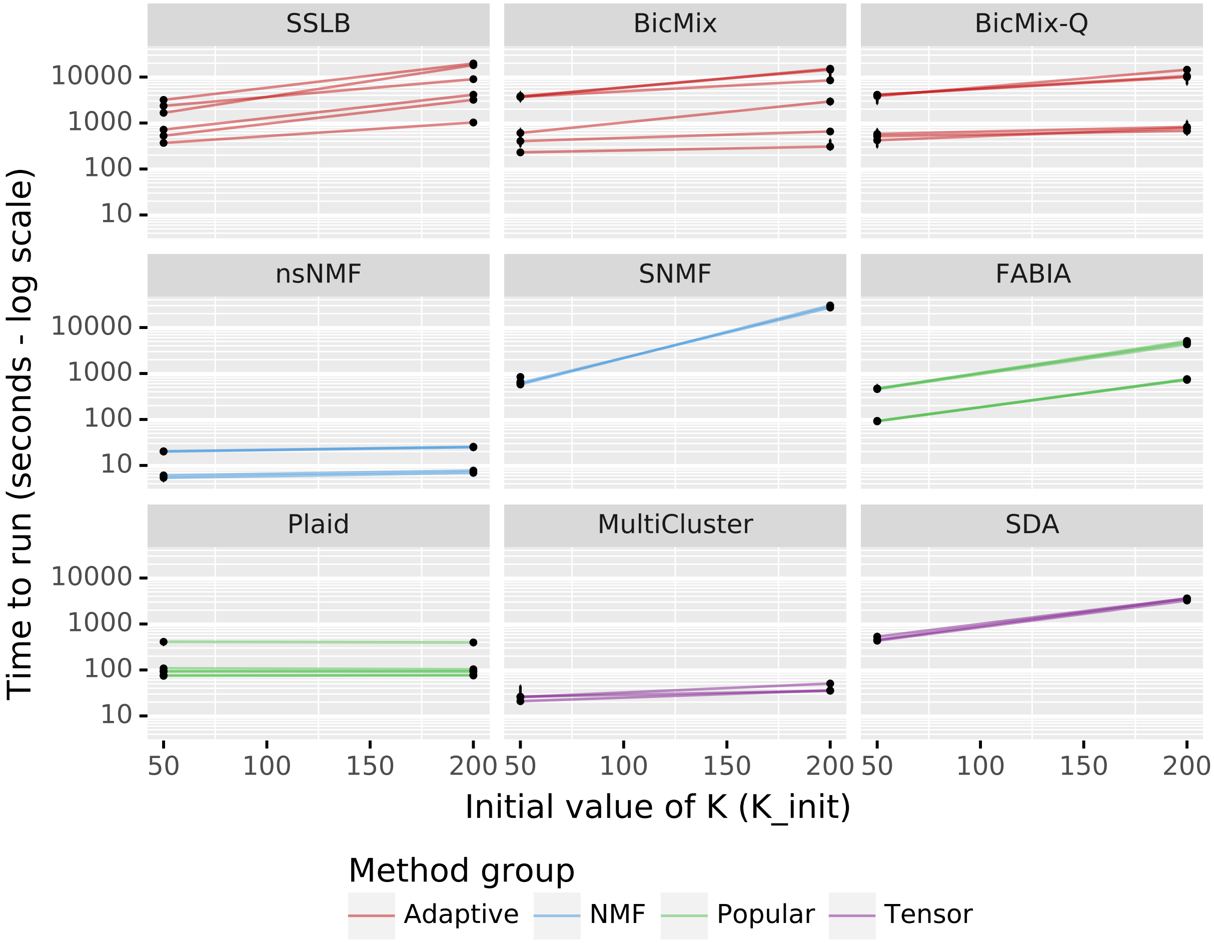

Figure S57: Effect of K_{init} on run time. Each line shows median run time (log scale) for a different IMPC dataset. The flatter the line, the less sensitive the runtime is to the choice of K_{init}. Note that MultiCluster and SDA couldn't be run on the non-tensor datasets.

References

[1] Sepp Hochreiter, Ulrich Bodenhofer, Martin Heusel, Andreas Mayr, Andreas Mitterecker, Adetayo Kasim, Tatsiana Khamiakova, Suzy Van Sanden, Dan Lin, Willem Talloen, Luc Bijnens, Hinrich W. H. Göhlmann, Ziv Shkedy, and Djork-Arné Clevert. FABIA: Factor analysis for bicluster acquisition. *Bioinformatics*, 26(12):1520–1527, June 2010. ISSN 1460-2059, 1367-4803. doi: 10.1093/bioinformatics/btq227.

[2] Chuan Gao, Ian C. McDowell, Shiwen Zhao, Christopher D. Brown, and Barbara E. Engelhardt. Context Specific and Differential Gene Co-expression Networks via Bayesian Biclustering. *PLOS Computational Biology*, 12(7):e1004791, July 2016. ISSN 1553-7358. doi: 10.1371/journal.pcbi.1004791.

[3] Artin Armagan, David B. Dunson, and Merlise Clyde. Generalized Beta Mixtures of Gaussians. *Advances in neural information processing systems*, 24:523–531, 2011. ISSN 1049-5258.

[4] Carlos M. Carvalho, Nicholas G. Polson, and James G. Scott. The horseshoe estimator for sparse signals. *Biometrika*, 97(2):465–480, 2010. ISSN 0006-3444.

[5] Gemma E Moran, Veronika Ročková, and Edward I George. Spike-and-Slab Lasso Biclustering. *Annals of Applied Statistics*, 2021.

[6] Veronika Ročková and Edward I. George. The Spike-and-Slab LASSO. *Journal of the American Statistical Association*, 113(521):431–444, January 2018. ISSN 0162-1459. doi: 10.1080/01621459.2016.1260469.

[7] Yee Whye Teh, Dilan Grür, and Zoubin Ghahramani. Stick-breaking Construction for the Indian Buffet Process. In *Artificial Intelligence and Statistics*, pages 556–563, March 2007.

[8] Thomas L. Griffiths and Zoubin Ghahramani. The Indian Buffet Process: An Introduction and Review. *Journal of Machine Learning Research*, 12(Apr):1185–1224, 2011. ISSN ISSN 1533-7928.

[9] Victoria Hore, Ana Viñuela, Alfonso Buil, Julian Knight, Mark I McCarthy, Kerrin Small, and Jonathan Marchini. Tensor decomposition for multi-tissue gene expression experiments. *Nature genetics*, 48(9):1094–1100, September 2016. ISSN 1061-4036. doi: 10.1038/ng.3624.

[10] Joseph Lucas, Carlos Carvalho, Quanli Wang, Andrea Bild, Joseph R. Nevins, Mike West, and Marina Vannucci. *Sparse Statistical Modelling in Gene Expression Genomics*, pages 155–176. Cambridge University Press, Cambridge, 2006. ISBN 978-0-511-58458-9. doi: 10.1017/CBO9780511584589.009.

[11] Hyunsoo Kim and Haesun Park. Sparse non-negative matrix factorizations via alternating non-negativity-constrained least squares for microarray data analysis. *Bioinformatics*, 23(12):1495–1502, June 2007. ISSN 1367-4803. doi: 10.1093/bioinformatics/btm134.

[12] A. Pascual-Montano, J.M. Carazo, K. Kochi, D. Lehmann, and R.D. Pascual-Marqui. Nonsmooth nonnegative matrix factorization (nsNMF). *IEEE Transactions on Pattern Analysis and Machine Intelligence*, 28(3):403–415, March 2006. ISSN 0162-8828. doi: 10.1109/TPAMI.2006.60.

[13] Miaoyan Wang, Jonathan Fischer, and Yun S. Song. Three-way clustering of multi-tissue multi-individual gene expression data using semi-nonnegative tensor decomposition. *The Annals of Applied Statistics*, 13(2):1103–1127, June 2019. ISSN 1932-6157. doi: 10.1214/18-AOAS1228.

[14] Amela Prelić, Stefan Bleuler, Philip Zimmermann, Anja Wille, Peter Bühlmann, Wilhelm Gruissem, Lars Hennig, Lothar Thiele, and Eckart Zitzler. A systematic comparison and evaluation of biclustering methods for gene expression data. *Bioinformatics*, 22(9):1122–1129, May 2006. ISSN 1367-4803. doi: 10.1093/bioinformatics/btl060.

[15] Doruk Bozdağ, Ashwin S. Kumar, and Umit V. Catalyurek. Comparative Analysis of Biclustering Algorithms. In *Proceedings of the First ACM International Conference on Bioinformatics and Computational Biology*, BCB '10, pages 265–274, New York, NY, USA, 2010. ACM. ISBN 978-1-4503-0438-2. doi: 10.1145/1854776.1854814.

[16] Wouter Saelens, Robrecht Cannoodt, and Yvan Saeys. A comprehensive evaluation of module detection methods for gene expression data. *Nature Communications*, 9(1): 1–12, March 2018. ISSN 2041-1723. doi: 10.1038/s41467-018-03424-4.

[17] George Pfundstein. *Ensemble Methods for Plaid Bicluster Algorithm.* Bachelor Thesis, University of München, 2010.

[18] Danilo Horta and Ricardo J.G.B. Campello. Similarity Measures for Comparing Biclusterings. *IEEE/ACM Transactions on Computational Biology and Bioinformatics*, 11(5):942–954, September 2014. ISSN 1545-5963. doi: 10.1109/TCBB.2014.2325016.

[19] Anne Patrikainen and Marina Meila. Comparing Subspace Clusterings. *IEEE Transactions on Knowledge and Data Engineering*, 18(7):902–916, July 2006. ISSN 1041-4347. doi: 10.1109/TKDE.2006.106.

[20] Paul Jaccard. The Distribution of the Flora in the Alpine Zone. *New Phytologist*, 11 (2):37–50, 1912. ISSN 1469-8137. doi: 10.1111/j.1469-8137.1912.tb05611.x.

[21] James Munkres. On the assignment and transportation problems (abstract). *Naval Research Logistics Quarterly*, 4(1):77–78, 1957. ISSN 1931-9193. doi: 10.1002/nav.3800040112.

[22] Peter S. Linsley, Cate Speake, Elizabeth Whalen, and Damien Chaussabel. Copy Number Loss of the Interferon Gene Cluster in Melanomas Is Linked to Reduced T Cell Infiltrate and Poor Patient Prognosis. *PLOS ONE*, 9(10):e109760, October 2014. ISSN 1932-6203. doi: 10.1371/journal.pone.0109760.

[23] Jesús S. Aguilar-Ruiz. Shifting and scaling patterns from gene expression data. *Bioinformatics*, 21(20):3840–3845, October 2005. ISSN 1367-4803. doi: 10.1093/bioinformatics/bti641.
